# From Neglecting to Including Cultivar-Specific Per Se Temperature Responses: Extending the Concept of Thermal Time in Field Crops

**DOI:** 10.34133/plantphenomics.0185

**Published:** 2024-06-01

**Authors:** Lukas Roth, Martina Binder, Norbert Kirchgessner, Flavian Tschurr, Steven Yates, Andreas Hund, Lukas Kronenberg, Achim Walter

**Affiliations:** ETH Zurich, Institute of Agricultural Sciences, Universitätstrasse 2, 8092 Zürich, Switzerland.

## Abstract

Predicting plant development, a longstanding goal in plant physiology, involves 2 interwoven components: continuous growth and the progression of growth stages (phenology). Current models for winter wheat and soybean assume species-level growth responses to temperature. We challenge this assumption, suggesting that cultivar-specific temperature responses substantially affect phenology. To investigate, we collected field-based growth and phenology data in winter wheat and soybean over multiple years. We used diverse models, from linear to neural networks, to assess growth responses to temperature at various trait and covariate levels. Cultivar-specific nonlinear models best explained phenology-related cultivar–environment interactions. With cultivar-specific models, additional relations to other stressors than temperature were found. The availability of the presented field phenotyping tools allows incorporating cultivar-specific temperature response functions in future plant physiology studies, which will deepen our understanding of key factors that influence plant development. Consequently, this work has implications for crop breeding and cultivation under adverse climatic conditions.

## Introduction

To mitigate the effects of global environmental change on crop production, a profound understanding of its influence on plant growth is required [1]. Crop models promise to be a versatile tool in analyzing and predicting plant growth [2], in particular for future climate scenarios [3,4]. However, the choice of models represents a challenging trade-off between biological realism and the principle of parsimony [5].

**Fig. 1.**
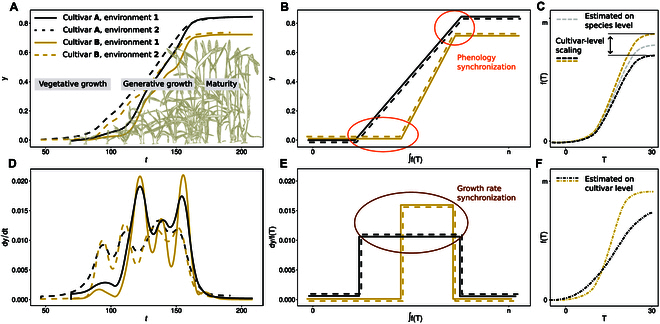
Schematic visualization of strategies in crop modeling to compensate for fluctuating temperatures on the example of generative growth in winter wheat. Plant growth over time appears nonlinear (A) and reveals irregular, potentially cultivar, and environment specific growth rate patterns (D). By replacing time *t* with the area under the curve of a temperature-compensation function, ∫ *f*(*T_t_*), phenology stages (B) and growth rates (E) may be synchronized in respect to the independent variable. Temperature-compensation functions can be based on species-level dose-response curves scaled to cultivar-level intrinsic growth rates (C), or on cultivar-specific dose-response curves (F).

From a temporal (i.e., growth-process-based) perspective, plant growth appears nonlinear (Fig. 1A). Rapid short-term changes in environmental conditions result in related short-term growth patterns [6]. These patterns are superimposed on seasonal changes of environmental conditions. On top of these relations, stress conditions may result in yet another superimposed (and potentially negative) growth pattern [7].

Finally, temporal patterns are also caused by advancing plant development, known as phenology. Fundamental influencing factors in cultivar-specific phenology include photoperiod sensitivity [8], and, in the case of winter cereals, vernalization requirements [9]. If modeling phenology on a rather small scale in environments with neglectable differences in photoperiod and vernalization, temperature remains as a dominant driver of phenology [10,11].

Consequently, a common modeling approach is to temperature-compensate time, thus “linearize” growth (Fig. 1E) and phenology (Fig. 1B) using a species-specific per se temperature growth response (Fig. 1C) [12]. Differences in growth rates and phenology between cultivars are then modeled using cultivar-specific factors that scale the predicted growth rate to measured (i.e., cultivar “intrinsic”) growth rates [13]. While it was shown that extending linear temperature responses (i.e., thermal time) to nonlinear functions can further improve predictions [14], Parent and Tardieu [13] provided evidence that modeling a per se temperature response at species level is sufficient. They speculated that evolutionary processes may have fixed the response for lower plant systematic levels. Hence, using linear and nonlinear temperature compensation functions with fixed, literature-based parameters seems justified.

Nevertheless, there is evidence that phenology is related to cultivar-specific temperature responses [15]. Consequently, one may assume that selecting for phenology traits in breeding—such as earlier flowering in winter wheat—co-selected for temperature response [16]. Indeed, we have repeatedly observed cultivar-specific temperature responses in our outdoor, high-throughput phenotyping site at ETH Zurich. We found cultivar-specific differences in the temperature response in the early canopy development of winter wheat [6,17], as well as in the stem elongation phase of winter wheat ([15]; [18]) and soybean [19]. Furthermore, we found that the differences in the stem elongation phase of winter wheat were related to the breeding origin of cultivars [16] and allow a “phenomic prediction/selection” for yield [20].

Similar observations have been made in crop modeling for other crops than wheat and soybean. Wallach et al. [21] could demonstrate that it is feasible to include a cultivar-specific temperature response parameter (*T*_opt_) for flowering time predictions in common bean. Viswanathan et al. [22] were able to optimize 2 temperature response parameters (*T*_min_ and *T*_opt_) for 2 growth stages in maize.

Given these evidences, it is striking how rarely temperature response parameters are included in the optimization process in crop growth and phenology models. Reasons may be found in the state-of-the-art use of so-called multienvironmental trials where the phenology of cultivars is measured in different environments. The heterogeneity of the environments often requires including other factors such as photoperiod sensitivity and vernalization requirements [23]. The additional inclusion of temperature response parameters will bring the number of parameters that require optimization close to the degrees of freedom of the data. One suggestion to overcome this limitation is to incorporate the genetic relatedness of cultivars, e.g, using loci associated with quantitative traits (QTLs) [21] or whole-genome predictions [24].

Another way to address the problem is to massively increase the number of data points per cultivar and environment. As phenology consists of single events, this can only be achieved by measuring continuous growth instead. Field-based plant organ tracking devices [6,25] and field-phenotyping platforms [26] can provide such dense time series with tens to thousands of growth rate / temperature value pairs. Simulation data [18] and real-world data analysis [16,27] have shown that, provided the temporal density of the time series is high enough, a few environments are sufficient to reliably determine cultivar-specific responses.

The question arises, whether transferring such precalibrated cultivar-specific temperature responses—determined on dense time series in few environments—to crop models may improve phenology predictions. Studies reporting phenology stages in species-specific thermal time often found severe genotype-by-environment (G×E) interactions in their data [28–30]. We suspect that large portions of the reported G×E interactions in phenology are artifacts of overgeneralizing the per se temperature response on species level. In other words, when explaining the observed performance of plants in different environments, thermal time is reificated—its abstract concept is by mistake treated as being a real, interpretable object.

**Fig. 2.**
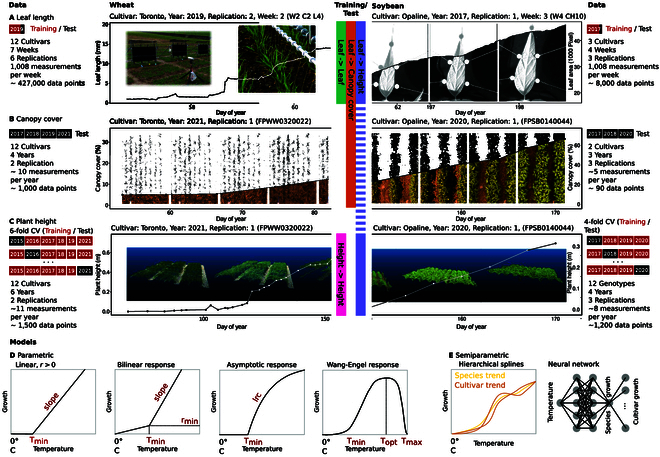
Evaluated dataset for winter wheat and soybean. Leaf elongation and growth was measured using a leaf length tracker device for wheat and a leaf growth tracker device for soybean (A). Canopy cover observations were taken using a field phenotyping platform to collect RGB images, followed by segmenting them in pixels showing plants and soil (B). Plant height measurements were performed using a phenotyping platform based terrestrial laser scanner and drone-based Structure-from-Motion (SfM) techniques (C). Four parametric dose-response models (D) and 2 semiparametric dose-response models (E) were evaluated. Model testing was performed on unseen data in test-train splits (A and B) and CVs (C).

If our assumption holds, using cultivar-specific linear or nonlinear temperature responses will improve the estimation of growth rates (Fig. 1E) and of phenology stages (Fig. 1B). To test this hypothesis, we evaluated a unique, temporally very dense multienvironment (i.e., one site, multiple years) outdoor winter wheat and soybean dataset. The setup corresponds to the specific situation where the prediction is to be improved for a clearly defined environment (in our case, Switzerland). Measurements and predictions are made under near-constant photoperiod and vernalization conditions, allowing to focus on temperature response only. The data were collected with temporally resolved leaf growth tracking devices as well as high-throughput field phenotyping devices (Fig. 2). We evaluated the response of traits to temperature using models of increasing complexity.

Examples for temperature response models with low complexity are the thermal time model [31] and the bilinear model. Both models assume a threshold temperature below which growth is zero, and a linear or bilinear increase of growth with temperature afterward. These models are easy to fit and robust, requiring only 1 to 3 parameters to estimate. However, a drawback of such models is that they assume infinite growth at infinitely high temperatures. The asymptotic model [18] and Wang–Engel model [14] correct for this flaw by assuming an intrinsic growth rate at the optimum temperature that is never exceeded. The Wang–Engel model also assumes decreasing growth rates after reaching the optimum temperature. Fitting such nonlinear models with 4 or more parameters can be challenging.

Splines represent a semiparametric alternative to nonlinear parametric models and have showed great potential to model plant growth data [32]. Still, spline models assume a certain smoothness and consequently shape of the response in relation to a covariate. In contrast, neural network regressors can be trained on datasets without assuming a specific function to fit the data. Instead, they learn the shape of the response directly from data. This overcomes the potential bias of selecting one specific response function. However, neural networks are prone to overfitting and require regularization techniques and large amounts of data to generalize well.

In addition to the model, the trait level (leaf growth, canopy development, and stem elongation), the covariate level (soil temperature and air temperature) and the covariate measurement level (below/inside canopies and at a reference weather station) were varied. All models were tested on unseen data in cross-validation (CV) runs (leaf growth: 15% unseen, plant height: 20% to 30% unseen, canopy cover: 100% unseen). Finally, phenology period estimations for the 3 main growth phases of wheat (vegetative growth, generative growth, and maturity) were performed using the preparameterized temperature response models.

## Materials and Methods

The increase of a trait *y* related to genotype *i* with time *t* in a steady-state growth phase can be modeled using a dose-response function *r* of covariates $\vec{x}$ and cultivar-specific crop growth parameters ${\vec{\theta }}_{i}$ [33],

$$y_{\mathrm{it}}=\int_{t_{0}}^{t}‍r\left({\vec{\theta }}_{i};{\vec{x}}_{t^{\prime }}\right)\;{\mathrm{dt}}^{\prime }.$$ (1)

In this framework, complexity may be varied at 4 levels:

• The trait *y* can be measured at different scales, e.g., at plant organ level or plant stand (canopy) level.

• The dose-response function *r* can vary in complexity, e.g., using linear regressions, nonlinear regressions, semiparametric splines [32], or neural network regressions.

• The covariates $\vec{x}$ can be measured at a different scale, e.g., close to the growing meristem, at the experimental unit (plot) level, or at a reference station.

• The crop growth parameters ${\vec{\theta }}_{i}$ can be estimated at a different scale, e.g., at variety/genotype level *i*, or at species level.

In the following, we pursue this structure, describing how traits were measured, complexity varied, and growth and phenology modeled.

Note that in Eq. 1, ${\vec{\theta }}_{i}$ was defined as cultivar-specific parameter set. As such, the dose-response function *r* will both model response per se (e.g., the base temperature below growth stops) and intrinsic growth rate differences between cultivars (e.g., absolute growth at optimum temperature) (Fig. 1F). When replacing ${\vec{\theta }}_{i}$ with a species-level parameter set $\vec{\theta }$, *r* reduces to a function that models relative growth rates. Thermal time is one example of such a function. To scale these relative growth rates to cultivar-specific intrinsic growth rates, one has to scale *r* to *y_it_* using a cultivar-specific factor *g_i_* (Fig. 1C),

$$y_{\mathrm{it}}=\int_{t_{0}}^{t}‍g_{i}\cdot r\left(\vec{\theta };{\vec{x}}_{t^{\prime }}\right)\;{\mathrm{dt}}^{\prime }.$$ (2)

To allow a comparison of species-level and cultivar-level models, Eq. 1 was used for cultivar-level models, and Eq. 2 with the cultivar-level parameter *g_i_* for species-level models. For further details, please see Eqs. 8, 9, and 12.

### Materials

All experiments were performed at the ETH research station for plant sciences Lindau-Eschikon, Switzerland (“Eschikon”; 47.449 °N, 8.682 °E, 520 m a.s.l.) on the field of the field phenotyping platform “FIP” [26]. The wheat experiment comprised a set of 12 varieties (CH Claro, CH Nara, Fastnet, Marksman, Ostka Strzelecka, Romanus, Runal, Rywalka, Semafor, Tamaro, Toronto, and Winnetou), replicated 2 times per year, and cultivated in 2015 to 2021 as subset of a larger experiment [15,34,35]. For leaf elongation tracking, the 12 varieties were additionally grown in 4 small plots beside the main experiment in 2019. These plots (0.9 m × 1 m) contained 3 cultivars each and were sown by hand in stripes of 0.3 m × 1 m.

The soybean experiment comprised a set of 3 varieties (Castetis, Gallec, and Opaline), replicated 3 times per year, cultivated in 2017 to 2020 as subset of a larger experiment with 36 genotypes [36]. Leaf growth tracking was performed directly in the main experiment in 2017.

Field management for both winter wheat and soybean was performed according to conventional farming practice in Swiss agriculture (see Supplementary Materials, Site FIP). Treatments included fertilizer, herbicide, insecticide, and fungicide treatments, but no irrigation.

### Trait measurements

#### Leaf length tracking in winter wheat

A common way to measure leaf growth in grasses is to track the linear extension of single leaves. Such monitoring can for example be done by attaching a clip to the leaf tip and monitor its movement [6]. Leaf elongation rates of 12 wheat cultivars were measured in the field from mid-February to beginning of April 2019 using the leaf length tracker system described by Nagelmüller et al. [6]. The installation followed the principle of an auxanometer. Briefly, the youngest leaf was attached to a hairpin to which a thread was attached. The thread was guided over several rollers along the panel and held taut with a counter weight (20 g). At the other end, the thread was attached to a white bead that moved over the panel in accordance with the elongation of the leaf. A waterproof CCTV camera (Lupusnet HD - LE934, CMOS sensor, maximal resolution of 1,920 × 1,080 pixels, Lupus-Electronics GmbH, Germany) took images of the panel every 2 min. A custom software (https://sourceforge.net/projects/leaf-length-tracker/) evaluated the position of the beads from the pictures, as they were used as indirect artificial landmarks to measure leaf elongation rate. Measurements were performed on in average 6 replications over 7 weeks (Fig. 2A).

Measured growth rates were corrected for weight-temperature interaction effects based on a calibration performed in a climate chamber. In this calibration setup, the growth of undisturbed leaves was compared with the growth of leaves where a force-equivalent of 20 g was applied. The differences in measured growth rates suggested a cultivar-unspecific correction of 0.004 mm/h per °C.

#### Leaf growth tracking in soybean

In contrast to the leaves of monocotyledons, the leaves of dicotyledons grow 2-dimensional. A common approach to measure such leaf growth is to fix single leaves in a 2-dimensional plane with small weights and monitor the leaf area. Leaf growth rates of 3 soybean cultivars were measured in the field from the beginning of June to mid-July 2017 using the leaf growth tracker (MARTRACK) system described by Mielewczik et al. [25]. Briefly, beads connected to threads were glued to the emerging leaves and fixed in front of a camera using a wire frame. The same cameras as above were used to record images every 2 min. A custom software (https://sourceforge.net/projects/martrackleaf/) evaluated the position of the beads from the pictures. Leaf area was then calculated based on the convex hull of bead positions in the planar image space. Relative growth rates were calculated based on differences of logarithmic leaf areas of 2 successive time points, divided by the time difference. Measurements were performed on in average 3 replications over 4 weeks (Fig. 2A).

#### Canopy cover monitoring based on RGB imaging in winter wheat and soybean

Canopy cover increase was monitored using the high-throughput field phenotyping platform “FIP”. The FIP platform is–among other sensors—equipped with an RGB camera (EOS 5D Mark II, 35-mm lens, Canon Inc., Tokyo, Japan). Plots were monitored with this sensor from a distance of 3 m to the ground. This setting results in a ground sampling distance of 0.3 mm/pixel. In the early canopy growth phase, in average 10 measurements per year (2017 to 2019, 2021) were taken for winter wheat, and in average 5 measurements per year (2017 to 2018, 2020) for soybean. RGB images were segmented pixel-wise into a plant and a soil fraction using a deep convolutional neural network [37].

Camera exposure positions and plant heights may change in the season due to inaccuracies of the camera carrier system and plant growth. As a consequence, the position of plots in images can vary from image to image. To enable a pixel-precise extraction of plot canopy cover values, image time series were first aligned using planar homography. Then, plot-specific shapes were projected to image time points. As feature detection algorithm, SIFT [38] and ORB [39] were used. Feature matching was performed using RANSAC [40]. Subsequently, the segmented and cutout image parts showing individual plots were further rectified by rotating them step-wise (-1.5° to 1.5° in steps of 0.2°) to maximize the distance between the minimum and maximum of plant pixels in image columns. For canopy cover extraction in winter wheat, only the inner 7 rows (of 9 rows per meter) were considered. For soybean with larger row spacing (3 rows per meter), only the inner row and half of both outer rows were used for further processing.

Canopy cover was then calculated as plant pixel ratio per plot. For winter wheat, measurements between approximately the beginning of the year to mid-April were considered, and for soybean, measurements between approximately mid-May and end of June. Only positive values, i.e., only canopy increase, were used for further processing. All processing was performed in Python using OpenCV and scipy [41].

#### Plant height monitoring in winter wheat and soybean

Plant height increase was monitored using the high-throughput field phenotyping platform “FIP” as well as drones. The FIP platform is—among other sensors—equipped with an terrestrial laser scanning device. The first 3 years of plant height measurements in winter wheat were collected with this device [15,19,34]. From the resulting point clouds, the percentile best matching manual measurements (97th percentile, [34]), was extracted per plot as plant height estimation per time point. For the subsequent years of winter wheat experiments and for all soybean experiments, drone-based Structure-from-Motion was used [16,42]. From the resulting point clouds, the percentile best matching manual measurements (90th percentile, [42]) per plot was extracted as plant height estimation per time point.

For wheat, measurements were performed on 2 replications on in average 11 time points per year (2015 to 2019, 2021) that fell into the stem elongation phase (Fig. 2C). For soybean, measurements were performed on 3 replications on in average 8 time points per year (2017 to 2020) that fell into the stem elongation phase (Fig. 2C). This included measurements between approximately mid-April and end of May for wheat and mid-June to mid-July for soybean.

Plot time series were smoothed using P-splines with the R package scam [43] before further processing to reduce prediction errors origin from autocorrelations of measurement errors.

#### Phenology measurements and estimations in winter wheat

Heading and senescence measurements were performed manually by trained persons. Heading was defined as the time point when the inflorescence was fully emerged for ≥50% of all shoots (GS 59) [44]. Heading was measured for all years (2015 to 2019 and 2021) on 1 to 2 replications.

Senescence was defined as the time point where the senescence of the central plot area has reached its midpoint [45]. Senescence was assessed on 2 replicates in 2016, 2017, and 2018.

The start of the stem elongation was estimated for genotypes in 2 replications based on plant height data using the quarter-of-maximum-elongation rate method described in [33] and [16] for the years 2017 to 2019 and 2021. For 2015 and 2016, no detailed plant height data for the early season were available, wherefore the start was approximated for all genotypes alike (2015 April 28 and 2016 April 15).

### Covariate measurements

Reference air temperature ($T_{\mathrm{air}}^{\mathrm{ref}}$) at the local weather station (in close proximity to the experimental field) was measured above a grass strip at 0.1 m above ground using Campbell CS215 sensors (Campbell Scientific Inc., U.S.A.). Air temperature inside the experiment ($T_{\mathrm{air}}^{\text{plot}}$) was measured in 2 to 4 wheat plots at 0.1 m above the ground, therefore above the plants before the start of the stem elongation, and inside the canopy for later growth stages, using Campbell CS215 sensors. Reference soil temperature ($T_{\text{soil}}^{\mathrm{ref}}$) was measured 0.05 m below ground at 3 reference positions below grass strips using Sentek/Hydrolina soil sensors (Sentek Sensor Technologies, Australia). Soil temperature inside the experiment ($T_{\text{soil}}^{\text{plot}}$) was measured 0.05 m below ground in 2 to 4 wheat plots using Sentek/Hydrolina soil sensors. Values of measurements performed at multiple locations (plots or reference positions) were averaged.

### Growth modeling

#### Dose-response models

As baseline model, thermal time based on hourly temperature recordings was used,

$$r_{\text{linear}}\left(T\right)=\max\left(\left(T-T_{\min}\right)\cdot a,0\right),$$ (3)

where *T*_min_ is the base temperature of growth, *a* the slope, and max(, 0) prevents negative growth rate predictions by replacing values lower than zero with zero. This model was called “thermal time” if *a* = 1 and *T*_min_ was set to the literature based threshold temperature of 0 °C for winter wheat [46] and 5 °C for soybean [47], and “linear model” if *T*_min_ and *a* were estimated based on own data.

To account for lower growth rates at temperatures close to zero that were observed in leaf length tracker data, the linear model was extended to a bilinear model,

$$\begin{array}{c} r_{\mathrm{bi}-\text{linear}}\left(T\right)=\max\left(T/T_{\min}\cdot r_{\min},r_{\min}+\left(T-T_{\min}\right)\cdot a,0\right), \end{array}$$ (4)

where *r*_min_ is a growth rate ≥0 at *T*_min_.

Plant height growth rate modeling has shown that an asymptotic model can approximate a Wang–Engel model given that temperatures do not exceed to supra-optimal growth ranges [18]. The asymptotic models is defined as

$$\begin{array}{c} r_{\text{asym}}=\max\left(r_{\max}\cdot \left(1-\text{exp}\left(-\exp\left(s\right)\cdot \left(T-T_{\min}\right)\right)\right),0\right), \end{array}$$ (5)

where *r*_max_ is the maximum absolute growth rate (and therefore the asymptote of the curve), *T*_min_ the base temperature where the growth rate is zero, and *s* characterizes the steepness of the response (natural logarithm of the rate constant, thus “*lrc*”) [48].

Finally, the original Wang–Engel model [14] is defined as

$$\begin{array}{c} \begin{array}{c} r_{\text{Wang}-\text{Engel}}\left(T\right)\;=\;r_{\max}\cdot \frac{2{\left(T\;-\;T_{\min}\right)}^{\alpha }{\left(T_{\mathrm{opt}}\;-\;T_{\min}\right)}^{\alpha }-{\left(T\;-\;T_{\min}\right)}^{2\alpha }}{{\left(T_{\mathrm{opt}}\;-\;T_{\min}\right)}^{2\alpha }}\\ \;\alpha \;=\;\frac{\text{ln}\left(2\right)}{\text{ln}\left(\left(T_{\max}-T_{\min}\right)/\left(T_{\mathrm{opt}}-T_{\min}\right)\right)}, \end{array} \end{array}$$ (6)

where *r*_max_ is the maximum absolute growth rate at the temperature optimum *T*_opt_, *T*_min_ the lower base temperature and *T*_max_ the upper base temperature of growth.

#### Model fitting to temporally resolved leaf growth tracking device data

Before training models, the data were split in training and test sets using a ratio of 6.37:1 for wheat and 6.31:1 for soybean, taking care that time series of replications/leaves stayed together in either the training or test set. The linear and nonlinear models defined above were then fitted to training data using maximum likelihood optimization. In contrast to previous attempts to process leaf growth tracking data [6,25], the raw measurement data were not smoothed. Instead, the measurement error was estimated using nested models with residual autocorrelation of order 1 to 3. The best fitting model was selected based on the Bayesian Information Criterion. Models were fitted using the base R [49] function mle.

In addition to the parametric linear and nonlinear models, 2 so called “semiparametric” models were trained. The first one was based on a hierarchical spline approach for longitudinal data [50] that models a general population trend, a genotype trend, and a replication trend ([32], R-code herein provided). We modified this approach in the way that time *t* was replaced by temperature *T* as “longitudinal dimension”, thus fitting hierarchical splines that represent dose-response curves.

The second “semiparametric” model approach was based on a multi-output neural network: A small network with 2 hidden layers of size 5 and sigmoid activation functions was trained to regress temperature on growth rates. An additional layer was then added to the network that transformed the single output in a multi-output of size 12 (one for each genotype). This layer was not activated, thus representing a linear transformation only. Training and validation sets were split with a 9:1 ratio, loss was calculated as mean squared error (MSE). L1 regularization was applied to the last layer. Optimization was done using the Adam optimizer in Pytorch Lightning with 1,500 epochs in pretraining and 800 epochs for fine-tuning with early stopping if the MSE did not improve by more than 0.0001 for 40 epochs. Initial learning rate was 0.05 with exponential decay with gamma 0.996, precision was 16 bit (half-precision), and batch size 2000. Early stopping was always reached.

#### Model fitting to canopy cover and height data

As for the leaf growth tracking data, canopy cover and height data were split in a training and test set. Here, the split was performed based on whole years, and repeated in a CV scheme. For wheat, this resulted in a 5:1 split ratio for plan height data in a 6-fold CV. For soybean, this resulted in a 3:1 split ratio for plan height data in a 4-fold CV.

The parametric models were then fit to the training set using a maximum likelihood approach that can fit high-resolution (hours) temperature courses and low-resolution (days) trait measurements [18].

An attempt to use canopy cover data to train models resulted in very poor estimates or failed convergence. Reasons for the lack of fit may be due to the plot time series being too short, incomplete, and noisy (Supplementary Materials Figs. S.1 and S.3). As a consequence, canopy cover data were only used for model testing and not for fitting.

### Model testing for growth predictions

Leaf growth tracking data originated from the same year. Consequently, the train/test split was used to calculate performance values by pooling all measurement values of the test set per cultivar. All other data were collected in differing years. Therefore, a random regression model was used for model testing. This approach was chosen based on the longitudinal character of plot-based time series, where one has to expect temporally and spatially correlated measurement errors [33].

For such measurements, a trait *y* is measured at repeated times *t* for genotypes *i* in the year *j* at the replication *k*. Time *t* is “linearized” using the different dose-response models, $r_{\mathrm{it}}=r\left({\vec{\theta }}_{i};T_{t}\right)$. Consequently, the difference between 2 consecutive measurements can be expressed as

$$\Delta y_{\text{ijkt}}=y_{\text{ijkt}}-y_{\text{ijkt}-1}=r_{\mathrm{it}}\times \left(\mu +g_{i}+v_{j}\right)+b_{j},$$ (7)

where *μ* is a fixed overall coefficient, *g_i_* and *v_j_* random coefficients related to genotypes and years, and *b_j_* a year-specific offset. The random coefficient structure was estimated using a variance-covariance structure among genotype replications *g_i_* and years *v_j_*. The model was fitted in R using ASReml-R [51].

The reported coefficients of determination of the predictions (*R*^2^ score) and root-mean-squared errors (RMSE) were based on the fixed overall coefficient *μ* for cultivar-level models,

$$\hat{\Delta }y_{\text{ijkt}}=r_{it}\times \mu ,$$ (8)

and on the fixed overall coefficient *μ* and random genotype coefficient *g_i_* for species-level models and thermal time,

$$\hat{\Delta }y_{\text{ijkt}}=r_{it}\times \left(\mu +g_{i}\right).$$ (9)

The *R*^2^ score was defined as

$$R^{2}=1-\frac{SS_{\mathrm{res}}}{SS_{\mathrm{tot}}}$$ (10)

where *SS*_tot_ the total sum of squared and *SS*_res_ is the residual sum of squares in relation to the 1:1 line.

### Model testing for phenology predictions

To test the prediction ability of phenology period duration, a linear mixed model was used. Such an approach can account for random sources of variation such as genotype effects and G×E interactions [52]. For phenology timing periods, 2 time points *t*_1_ and *t*_2_ are measured per genotype *i* in the year *j* at the replication *k*. Then, the time period in between is “linearized” using the different dose-response models, resulting in a new trait *y*,

$$y_{\mathrm{ijk}}=\int_{t_{\mathrm{ijk},1}}^{t_{\mathrm{ijk},2}}‍r\left({\vec{\theta }}_{i};T_{t},\right)\;{\mathrm{dt}}^{\prime },$$ (11)

where *t*_*ijk*,1_ is the start of the period and *t*_*ijk*,2_ the end of the period. Note that the new trait *y_ijk_* is on a genotype-specific scale. To allow comparison and variance decomposition, *y_ijk_* values were scaled to one per genotype. After this time period transformation, overall best linear unbiased predictions (BLUPs) were estimated using the model

$$y_{\mathrm{ijk}}=\mu +v_{j}+g_{i}+{\left(\mathrm{vg}\right)}_{\mathrm{ij}}+e_{\mathrm{ijk}},$$ (12)

where *μ* is a global intercept, *v_j_* a fixed year effect, *g_i_* a random genotype effect, and (*vg*)*_ij_* random genotype-environment effects modeled using a diagonal variance structure, allowing for differing genotypic variances for years. The residual variance structure *e_ijk_* was set to account for random row and range effects and random interactions of row and range effects, thus accounting for differing spatial gradients for years. The model was fitted in R using ASReml-R [51].

### Residual analysis based on environmental indices

Major developmental responses to environmental factors in winter wheat include growth responses to temperature, photoperiod, and vernalization [30]. Stress factors in European, and more specifically, Swiss environments include frost [53], heat [54], drought [55], and biotic factors such as diseases [56]. Consequently, if relying on data from a local weather station to characterize environments, temperature, precipitation, and global radiation measurements are among the most promising covariates.

The hereafter used environmental indices were calculated based on such data, i.e., daily mean, minimum, and maximum temperature, precipitation sum, and global radiation (Supplementary Materials, Table S1). Precipitation values were further transformed into the Standardised Precipitation Index (SPI) [57] and the Standardised Precipitation and Evapotranspiration Index (SPEI) [58] using the Thornthwaite transformation [59] to estimate evapotranspiration. SPI and SPEI were calculated using the R package SPEI [60]. Both indices were calculated with a 30-day smoothing to account for effects within each phenological period rather than long-term effects. For each index, the minimum, maximum and cumulative values per phenological period were calculated.

Furthermore, a cold stress index considering the temperature sum of minimum daily temperatures below 0 °C was added. Drought and moisture extreme indices were calculated using the sum of SPI and SPEI values above and below a threshold of 1 and 1.75, respectively -1 and -1.75. Negative values represent very moist periods and can therefore define wet seasons. Positive values indicate dry periods and can therefore correlate with periods of drought stress.

In addition to the environmental indices, the mean growth period duration in days per cultivar was added to the list of features. A lasso regression was then applied to the residuals of the phenology prediction models, using a lasso and elastic-net regularized generalized linear model from the R package glmnet [61]. The model was fitted using the R package caret [62] with a search grid for *λ* = 10^−8^ to 5 and *α* = 1 in a repeated CV with 10 repeats and 5 folds. Features were centered and scaled before fitting.

## Results

### Growth rate prediction

A first aim of the study was to identify suitable temperature response models to predict continuous growth, and to test for the transfer ability of these trained models from one growth stage to others. This step will provide insight into the importance of model choice, explaining covariates, and variety versus species-level.

Parametric models (Eqs. 3 to 6), hierarchical splines, and a neural network model were trained on leaf and plant height growth datasets (Fig. 2A and C). For all models, cultivar-specific (Eq. 1) and species-level (Eq. 2) variations were considered. Trained models were tested on unseen leaf, canopy, and plant height growth datasets (Fig. 2A to C). Tests were based on measured and predicted differences between consecutive measurements using random regressions (Eq. 7) that account for year effects (Eq. 8) and, in case species-level models were fitted, for cultivar-specific scaling (Eq. 9).

**Fig. 3.**
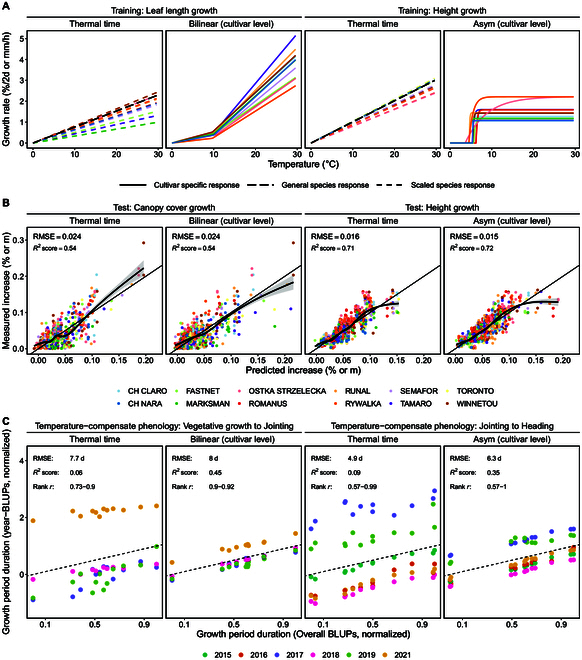
Overview of performance of the best species-level and cultivar-level models for winter wheat. While species-specific thermal time models (Eq. 2) where scaled to cultivar-specific intrinsic growth rates (Eq. 3), cultivar-level bilinear (Eq. 4) and asymptotic models (Eq. 5) were fitted for each cultivar separately (Eq. 1) (A). Models were tested on unseen growth datasets (B) and unseen phenology datasets (C) to indicate their potential to predict growth and to reduce estimated G×E in phenology. For a full comparison of model performance for both wheat and soybean, see Fig. 4 for growth predictions and Fig. 5 for phenology.

In summary, cultivar-level models and species-level models were equally well suited to model growth (Fig. 3). Scaling species-level models such as thermal time to cultivar-specific intrinsic growth rates successfully predicted growth for unseen test sets (Fig. 3B). However, fitted cultivar-level models showed clear per se temperature response characteristics, as indicated for example by cross-overs between cultivar-level response curves for the bilinear model (Fig. 3A). Using such cultivar-specific models to predict unseen growth data test sets resulted in similar performance as for species-level models (Fig. 3B). More important than the model choice was the choice of covariate (soil or air temperature) in relation to the growth phases. In the following, detailed results will be reported for wheat and soybean.

#### Wheat growth rate predictions

**Fig. 4.**
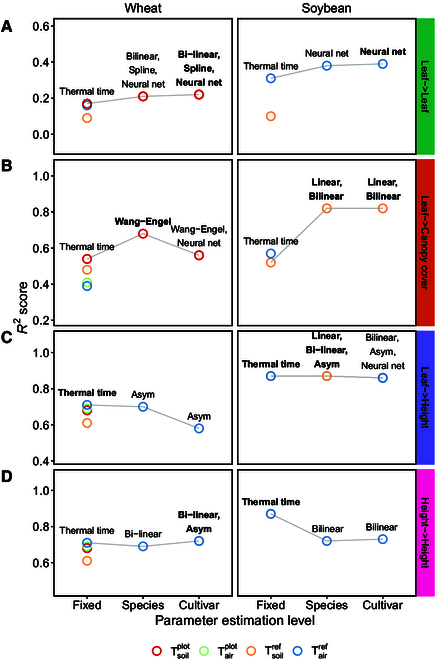
Performance of linear (Eq. 3) and nonlinear (Eqs. 4 to 6) models for growth rate predictions (Eq. 7) in winter wheat and soybean. Models were trained on leaf length data (A to C, Leaf→) and plant height data (D, Height→). Predictions were tested on unseen leaf length data (A, →Leaf), canopy cover data (B, →Canopy cover), and plant height data (C and D, →Height). The covariate temperature was measured in air (*T*_air_) and soil (*T*_soil_) at plot level (*T*^plot^) and at a reference station (*T*^ref^). Model parameters were either estimated on species (Eq. 9) or cultivar-level (Eq. 8) or based on literature and therefore fixed. Indicated are the coefficients of determination (*R*^2^ score) of predictions of the best performing temperature response model per training/test combination and parameter estimation level. Overall best performing models per training/test combination are indicated in bold.

**Fig. A.1.**
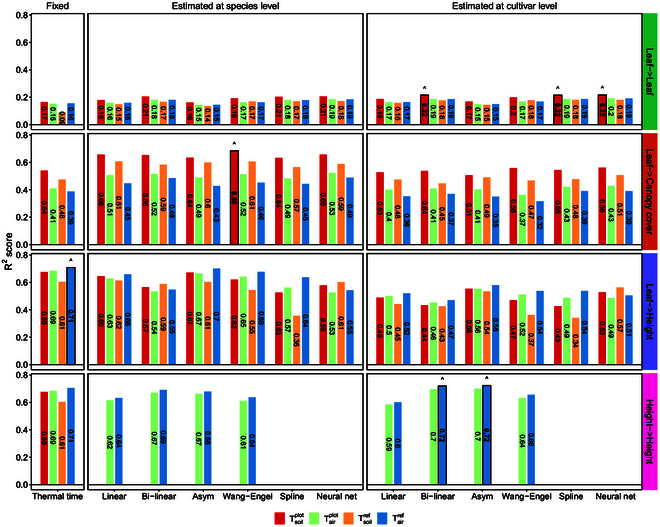
Performance of growth rate predictions for winter wheat. Models were trained on leaf length data (Leaf→) and plant height data (Height→). Predictions were tested on unseen leaf length data (→Leaf), canopy cover data (→Canopy cover), and plant height data (→Height). The covariate temperature was measured in air (*T*_air_) and soil (*T*_soil_) at plot level (*T*_plot_) and at a reference station (*T*_ref_). Indicated are the coefficients of determination (*R*^2^ score) of predictions based on the corresponding temperature response model. At the species-level, predictions were based on an overall coefficient and genotype coefficients (Eq. 9). For cultivar-level models, the genotype specificity is already incorporated in the response model, and predictions therefore based on an overall coefficient only (Eq. 8). Coefficients were fitted using a random regression model with random coefficients for years and plots and the mentioned fixed overall coefficient and genotype coefficients (Eq. 7).

Cultivar-specific response models outperformed species-level models if training and test sets were closely related (Fig. 4A, Appendix Fig. A.1). If training and test sets originated from the leaf elongation measurements, the highest growth prediction accuracy was reached by 3 cultivar-specific models: The bilinear model, the hierarchical splines, and the neural network (*R*^2^ = 0.22, RMSE = 0.39 mm/h). Relying on plot-based temperature measurements outperformed reference station measurements (Δ*R*^2^ = 0.08). Using plot-based soil temperatures slightly outperformed using plot-based air temperatures (Δ*R*^2^ = 0.01).

If training and test sets differed, species-level response models generalized better (Fig. 4B and C, Appendix Fig. A.1). When applying models trained on leaf elongation measurements to whole-canopy measurements made in the same growth phase, the species-level Wang–Engel model (*R*^2^ = 0.68, RMSE = 0.44%/day) outperformed the cultivar-level Wang–Engel and neural network models (Δ*R*^2^ =  −0.12) and thermal time (Δ*R*^2^ =  −0.14). Again, using soil temperature at the plot level resulted in a higher accuracy than using soil temperature or air temperature measured at a reference station (Δ*R*^2^ ≥ 0.06).

When further reducing the relatedness of training and test sets by predicting plant height growth with leaf elongation models, thermal time (*R*^2^ = 0.71, RMSE = 0.18 mm/day) performed slightly better than the asymptotic species-level model (Δ*R*^2^ =  −0.01) and the corresponding cultivar-level model (Δ*R*^2^ =  −0.13).

The advantage of cultivar-level models could be restored by training models directly on plant height data, thus using closely related training and test sets (Fig. 4D, Appendix Fig. A.1). Two cultivar-level models were suggested, the asymptotic and bilinear dose-response curves (*R*^2^ = 0.72, RSME: 0.16 mm/day). To predict plant height data, air temperature measured at the reference weather station was better suited than plot-based measurements (Δ*R*^2^ ≥ 0.02).

#### Soybean growth rate predictions

**Fig. A.2.**
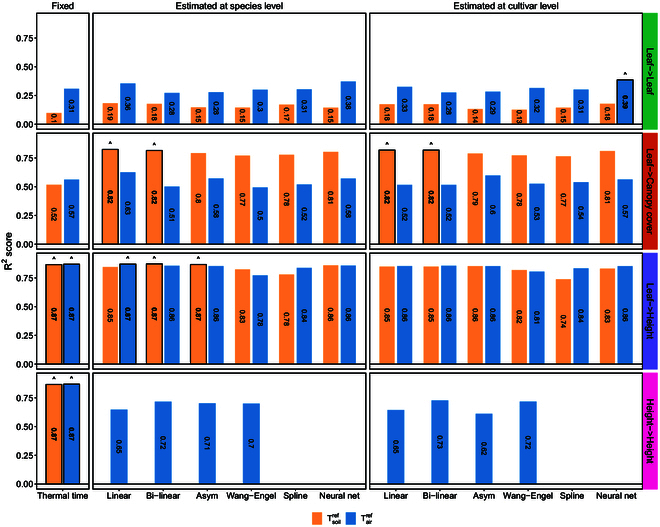
Performance of growth rate predictions for soybean. Models were trained on leaf area data (Leaf→) and plant height data (Height→). Predictions were tested on unseen leaf area data (→Leaf), canopy cover data (→Canopy cover), and plant height data (→Height). The covariate temperature was measured in air (*T*_air_) and soil (*T*_soil_) at a reference station (*T*_ref_). Indicated are the coefficients of determination (*R*^2^ score) of predictions based on the corresponding temperature response model. At the species-level, predictions were based on an overall coefficient and genotype coefficients (Eq. 9). For cultivar-level models, the genotype specificity is already incorporated in the response model, and predictions therefore based on an overall coefficient only (Eq. 8). Coefficients were fitted using a random regression model with random coefficients for years and plots and the mentioned fixed overall coefficient and genotype coefficients (Eq. 7).

As for the wheat dataset, the model performance in soybean was dependent on the relatedness of training and test sets. If the training and test set both originated from the leaf growth measurements (Fig. 4A, Appendix Fig. A.2), the cultivar-level neural network performed best (*R*^2^ = 0.39, RMSE = 0.46%/h). Using air temperature clearly outperformed soil temperature (Δ*R*^2^ = 0.21).

If training and test sets differed, species-level response models performed as good as cultivar-level response models (Fig. 4B and C, Appendix Fig. A.2). For canopy cover growth predictions, simple cultivar or species-specific models (linear and bilinear) performed best (*R*^2^ = 0.82, RMSE = 1.0%/day). Strikingly, while air temperature performed better than soil temperature for thermal time (Δ*R*^2^ = 0.05), for species and cultivar-specific models, growth was best predicted by soil temperature (Δ*R*^2^ = 0.30).

If further reducing the relatedness of training and test sets by predicting plant height with leaf growth models, species-level models were more accurate than cultivar-level models. Literature based, linear thermal time performed equally well as the 3 best species-level models; i.e., the linear, bilinear, and asymptotic model (*R*^2^ = 0.87, RMSE = 3.8 to 4.4 mm/day). Nevertheless, differences to the best cultivar-level model were very small (Δ*R*^2^ = 0.01).

In contrast to winter wheat, training models directly on soybean plant height data could not restore the advantage of cultivar-level models (Fig. 4D, Appendix Fig. A.2). While the bilinear model performed best on the species and cultivar-level (*R*^2^ = 0.73, RMSE = 5.1 mm/day), its performance was still worse than that of the thermal time model (*R*^2^ = 0.87).

### Phenology prediction

A second aim of the study was to test the hypothesis whether phenology is driven by the previously extracted cultivar-specific temperature responses or not. Time periods between successive growth stages (e.g., jointing to heading) per cultivar and year were either expressed in thermal time, using species-level nonlinear temperature response models, or using cultivar-level models (Eq. 11). Then, G×E interactions were estimated using a linear mixed model (Eq. 12).

Cultivar-level models showed a clear advantage over species-level models (Fig. 3C). While the prediction error between models was comparable, using cultivar-level models resulted in higher cultivar rank correlations between environments and better correspondences between overall BLUPs and year BLUPs. The findings indicate that temperature-compensating with cultivar-level models decreases the estimated G×E effects for phenology, while using thermal time inflates these effects. In the following, detailed results and consequences for G×E analysis are provided.

#### Severity of the estimated G×E interaction for different models

**Fig. 5.**
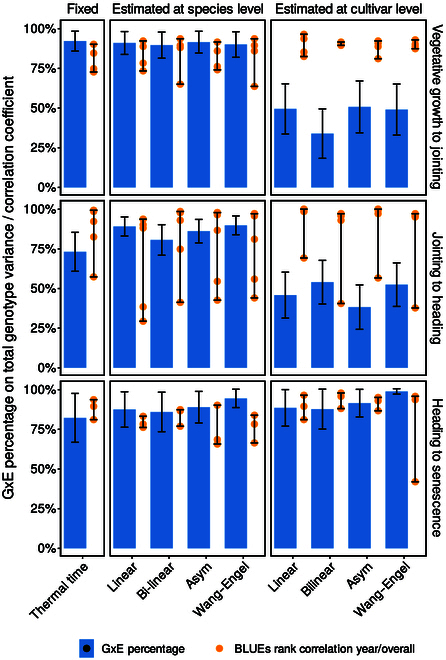
Performance of linear and nonlinear models for temperature compensated phenology period duration predictions (Eq. 11) in winter wheat. Predictions were based on cultivar-level best linear unbiased estimations (overall BLUPs) of a linear mixed model (Eq. 12) that included effects for cultivars (*g_i_*), years (*v_j_*), and year-cultivar interactions ((*vg*)*_ij_*). Indicated are the percentage of estimated G×E variance ($\sigma_{\left(\mathrm{vg}\right)}^{2}$) on the total genotypic variance ($\sigma_{\left(\mathrm{vg}\right)}^{2}+\sigma_{g}^{2}$), and Spearman’s rank correlations of year-specific (*v_j_* + *g_i_* + (*vg*)*_ij_*) versus overall (*g_i_*) phenology duration predictions (Eq. 12).

**Fig. 6.**
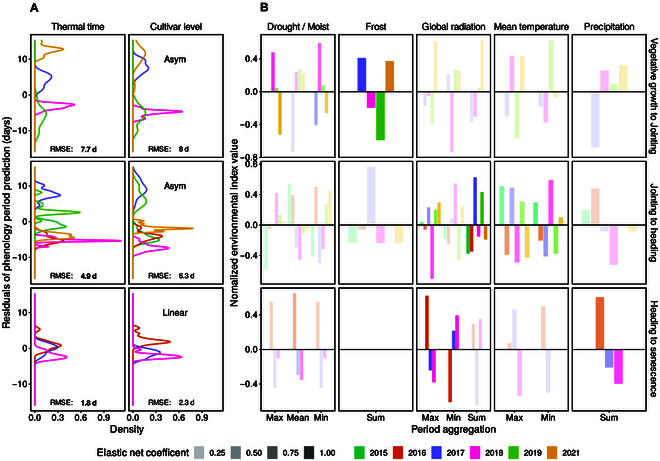
Temperature compensated phenology period duration predictions (Eq. 11) for winter wheat, and corresponding environmental indices. Period predictions are based on genotype effects plus the mean of year effects (*gi* + 1 / *j* ∑*vj*) estimated using a linear mixed model (Eq. 12). Indicated are (A) residuals of predictions for the thermal time model and the best performing cultivar-level model (asymptotic and linear) and related RMSE, and (B) environmental index values per year for indices that were suggested by an elastic net regression to best explain residuals of cultivar-level models. Residuals (A) and indices (B) with the same sign indicate a positive correlation (high index → extended period), differing signs indicate a negative correlation (high index → shortened period).

**Fig. A.3.**
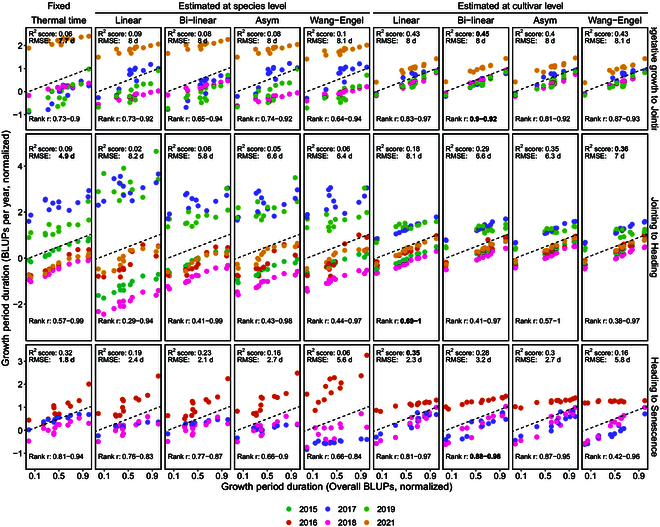
Performance of phenology period duration predictions for winter wheat. Predictions were based on cultivar-level best linear unbiased estimations (overall BLUPs) of a linear mixed model that included effects for cultivars, years, and year-cultivar interactions (Eq. 12). The linear mixed model itself was fitted to thermal time, cultivar-level temperature response model, and species-level temperature response model outputs from growth rate fits.

Using thermal time as temperature response model resulted in large estimated variances of G×E interactions (Fig. 5 and Appendix Fig. A.3). Depending on the growth stage, up to 73% to 82% of the total genotypic variance was related to G×E. Consequently, variety rank changes across years were frequent, and rank correlations between years and overall means varied widely (*r* = 0.57 to 0.99). RMSEs of predictions in calendar days were larger for earlier growth stages than for later growth stages (7.7 days for vegetative growth versus 1.8 days for maturity) (Fig. 6).

Using species-level dose-response curve models further increased estimated G×E variances (81% to 95%) (Fig. 5). Correspondingly, rank correlations between years and overall means did not improve (*r* = 0.29 to 0.97).

In contrast, cultivar-level temperature response models resulted in the lowest G×E estimations for 2 of 3 phenology periods (vegetative growth and generative growth) (Fig. 5A). Highest rank correlations and lowest G×E were found for the bilinear model in the vegetative phase (34%, *r* = 0.90 to 0.92) and the asymptotic model in the generative phase (38%, *r* = 0.57 to 1.00). Differences in RMSEs to thermal time were small (≤1.4 days) (Fig. 6).

#### Explainability of G×E interactions with other environmental factors

To investigate the sources of the estimated G×E interactions after temperature-compensating time, the residuals of the phenology predictions based on genotype effects and the mean of year effects (Eq. 12) were further decomposed in components related to environmental indices. For cultivar-level models, moist conditions and frost best explained differences in vegetative growth period duration values and delayed jointing (Fig. 6). Extended generative growth and hence delayed heading was mainly related to high global radiation values. For maturity, wet conditions and/or extremes in global radiation best explained delayed senescence.

Using thermal time as temperature response model instead of cultivar-level models resulted in weaker relations of residuals to environmental indices. Although predictions were temperature-compensated, remaining relations to temperature indices were indicated. For the last growth period, only temperature related indices were found to be relevant. Links to drought and limited global radiation—as indicated by the cultivar-level models—were entirely missing.

## Discussion

For both soybean and winter wheat, the results indicated an advantage of cultivar-specific nonlinear temperature response models if training and test sets were closely related. Using these cultivar-level models for wheat phenology predictions could substantially reduce the observed G×E interaction.

The nonlinearity of growth responses to temperature has long been suspected and investigated [63]. Conclusively, the herein found best performing response models for winter wheat are well known: The Wang–Engel model [64] that performed best in predicting canopy growth is known for its ability to accurately model winter wheat growth [14]. The asymptotic model that performed best in predicting plant height growth can be seen as simplified Wang–Engel model, given that temperatures do not exceed supra-optimal ranges [18]. In contrast, for soybean leaf growth, a neural network model performed best, indicating high degrees of freedom required to accurately predict responses. For measurements at the coarser canopy level, simpler linear and bilinear models were more accurate.

Nevertheless, as other authors noted before (e.g., [65]), the superiority of cultivar-level and nonlinear models was not given in all situations. In particular the transferability to other trait levels (i.e., plant organ versus canopy level) and to other growth phases (i.e., vegetative versus generative growth) appeared limited. Large confounding effects in the test sets are suspected. Physiological changes that are not directly related to plant organ growth may dilute cultivar-specific response signals (i.e., growth rates) on the canopy level. Examples are tillering/branching in the vegetative phase or lodging in the generative phase. Consequently, simpler models such as species-level thermal time generalize better in such situations.

Not only the model choice, but also the covariate measurement level choice may enhance generalization. Depending on the location of a sensor (e.g., in or above the ground, at a reference station, or within a canopy), the relationship between measured covariate values and actual biological processes may vary. For winter wheat, the shoot apical meristem is located below the soil surface for half of the lifetime. In contrast, the growing tissue in soybean is above-ground. Consequently, vegetative growth for winter wheat was best predicted using soil temperature, confirming the findings of Jamieson et al. [66]. Surprisingly, for soybean, soil temperature could predict canopy growth more accurately than air temperature. We suspect that this is due to the fact that air temperature measured at a reference station is less representative of in-canopy temperature than soil temperature. Soil temperature courses may also better match the diurnal growth patterns commonly found in soybean [67].

Finally, changing cardinal temperatures with time [54] are an additional concern in temperature response modeling. Indeed, for the different growth periods, the choice of model and covariate level changed, and models performed inferior if not trained on the same growth period data. Nevertheless, for predictions, the division of the crop growth cycle into 3 consecutive growth phases—vegetative growth, generative growth, and maturity—was sufficient to accurately predict phenology as well as growth rates.

In this work, 2 approaches to derive cultivar-specific responses were proposes, (a) plant organ tracker devices for the vegetative growth phase, and (b) high-throughput plant height measurements for the generative growth phase. Both methods have been proven to provide reliable estimations for their respective growth phases [6,18,25], and this work could confirm their readiness for application. For plant organ tracker approaches, measurements in a few weeks per cultivar are sufficient, while for height data, measuring in multiple years is inevitable [16]. Unfortunately, no such method is yet available for the maturity phase, indicating that further research is needed to measure temperature responses in the late season.

One aspect not explored in this work is how the genetic relatedness of varieties can be used to “borrow” information in modeling. It has been shown that crop growth modeling can benefit from the identification and integration of genetic QTLs. In this work, the parameters of the models were derived independently for each cultivar, ignoring the relatedness of the cultivars. In a QTL approach, one can avoid the need to estimate parameters for each genotype separately. Instead, model parameters can be expressed as functions of the QTLs (e.g., [68]), which allows the simultaneous estimation of parameters for all varieties [21]. However, such approaches require larger sets of cultivars (typically >150) than those used in this work.

In contrast to such QTL approaches, the high-throughput phenotyping and modeling approach proposed here allowed the extraction of genotype-specific temperature responses even for a small number of cultivars. Such responses can, for example, serve as baselines for stress monitoring. Given that temperature growth responses in unstressed conditions are known, one can use relative growth differences instead of absolute growth values. In such setups, the observed relation of G×E to the stressor will be less biased by temperature.

Indeed, after correcting for cultivar-level temperature responses, our data showed a clear clustering of environments for early or delayed phenology. Such a clustering was not found using species-level thermal time. For example, for the vegetative growth phase, the residuals of phenology period predictions for the years 2017 and 2021 were clearly separated from the years 2018 and 2019 (Fig. 6A). If comparing those 2 clusters regarding their environmental conditions, differences were best explained by frost and moist conditions (Fig. 6B). Such frost events, followed by measurable leaf area reductions, were indeed observed in the field in 2018 and 2021 [7].

In the generative growth phase, the years 2017 and 2019 were clearly separated from other years if using the cultivar-level asymptotic model but not if using thermal time. 2017 and 2019 had higher global radiance values than the other environments. These detected relations between heading date and global radiation are in accordance with Benaouda et al. [69] who found temperature to be the main driver and high global radiation to be the main delayer of heading.

Finally, for maturity, Anderegg et al. [45] reported a delayed senescence in the year 2016 due to the extraordinary wet year with severe Septoria tritici blotch (STB) disease pressure. Such a separation of environmental indices was also visible in our data (Fig. 6B). Still, the phenology prediction residuals for the year 2016 were only separated from the other years if using the cultivar-level linear model but not if using thermal time (Fig. 6A).

The primary research question addressed in this study is whether to ignore or incorporate cultivar-specific per se temperature responses when modeling. Based on the growth rate predictions (Fig. 3B), it appears reasonable to agree with Parent, Millet, and Tardieu [65] that the use of species-level thermal time has a sound theoretical basis. Neglecting cultivar-specific per se temperature responses seems justified. However, further testing the thermal time concept on phenology data substantially weakened the soundness of thermal time—ignoring cultivar-level per se responses inflated the estimated G×E interactions (Fig. 3C).

Based on our results, we have to conclude that ignoring cultivar-specific differences in temperature responses creates bias in follow-up investigations of other G×E interactions, such as those induced by for example frost or drought stress. The tools for assessing cultivar-specific per se temperature responses in real-world field conditions are widely available now. With this study, we give implications on how and why those tools should be applied. Consequently, the theoretical concept of thermal time can be taken to the next level, which is cultivar-specific.

## Data Availability

All data needed to evaluate the conclusions in the paper are present in the paper, the Supplementary Materials, and on https://gitlab.ethz.ch/crop_phenotyping/phenoflow_early_growth.

## Supplementary Materials

Supplementary 1 Site FIP Figs. S1 to S11 Tables S1 to S4
