# Supplementary material for: From Neglecting to Including Cultivar-Specific Per Se Temperature Responses: Extending the Concept of Thermal Time in Field Crops
Source: Plant Phenomics. 2024 Jun 1;6:0185. doi: 10.34133/plantphenomics.0185 (PMC11142864; doi:10.34133/plantphenomics.0185)
Supplement: Supplementary 1 — Site FIP Figs. S1 to S11 Tables S1 to S4 [file plantphenomics.0185.f1.zip › Supplementary_Data.pdf]

Site FIP

The site FIP is located at the ETH research station of agricultural sciences in Lindau Eschikon, Switzerland (47.449 N, 8.682 E, 556 m a.s.l.). The soil type is an eutric cambisol consisting of 21% clay and 21% silt. Organic matter content is 3.5% and pH 6.7. Soil characteristic were determined in 2015 (Eric Schweizer AG, Thun, Switzerland).

Winter wheat:

Preliminary to wheat (*Triticum aestivum L.*), soybeans (*Glycine max* (L.) Merr.) and buckwheat (*Fagopyrum esculentum* Moench) were grown. After preliminary crops were harvested, the soil was plowed and harrowed before wheat was drill-sown.

Wheat was sown in 9 rows per plot with a row length of 0.7 m and a row distance of 0.125 m. Sowing density was 370-400 plants m^{-2}. One day after sowing, herbicide (Herold SC, Bayer AG, Leverkusen, Germany) was applied to ensure weed free plots. Several fungicides and insecticides were applied in spring to ensure healthy plants. Fertilizer was split into 3 doses (~ 1:3:1), one at tillering stage, one at start stem elongation, and one after heading. No irrigation was applied.

	Sowing	Harvest	Fertilizer (N, P_2O_5, K_2O, Mg) (kg/ha)	Comment
2015	2014/10/20	2015/08/03	150, 79, 99, 21	
2016	2015/10/13	2016/07/27	143, 92, 120, 19	
2017	2016/11/01	2017/07/19	148, 92, 120, 20	
2018	2017/11/02	2018/07/14	136, 92, 120, 18	
2019	2018/10/17	2019/07/23	148, 92, 120, 64	1 x 5 m plots
2021	2020/10/21	2021/07/29	122, 0, 0, 100	

Soybean:

Preliminary to soybeans (*Glycine max* (L.) Merr.), winter wheat (*Triticum aestivum L.*) and subsequently a cover crop mixture was grown. After winter, cover crops were mulched. The soil was plowed and harrowed before soybean was drill-sown.

Soybean was sown in 9 or 3 rows per plot with a row length of 5 m and a row distance of 0.125 m respectively 0.5 m. Sowing density was 40-55 plants m^{-2}. One day after sowing, herbicide (Dual Gold & Molipan Pro, Syngenta, Basel, Switzerland) was applied to ensure weed free plots Several fungicides and insecticides were applied in spring to ensure healthy plants. Fertilizer was applied before sowing and harrowing. No irrigation was applied.

	Sowing	Harvest	Fertilizer (N, P_2O_5, K_2O, Mg) (kg/ha)	Comment
2017	2017/04/12	2017/09/27	0, 138, 240, 0	9 rows
2018	2018/04/19	2018/08/28	0, 138, 210, 0	9 rows
2019	2019/04/24	2019/09/24	0, 115, 210, 44	3 rows
2020	2020/04/15	2020/09/21	0, 92, 120, 75	3 rows
