# Supplementary material for: From Neglecting to Including Cultivar-Specific Per Se Temperature Responses: Extending the Concept of Thermal Time in Field Crops
Source: Plant Phenomics. 2024 Jun 1;6:0185. doi: 10.34133/plantphenomics.0185 (PMC11142864; doi:10.34133/plantphenomics.0185)
Supplement: Supplementary 1 — Site FIP Figs. S1 to S11 Tables S1 to S4 [file plantphenomics.0185.f1.zip › Supplementary_Figure_and_Tables.pdf]

B. Supplementary Materials

B.1. Figures

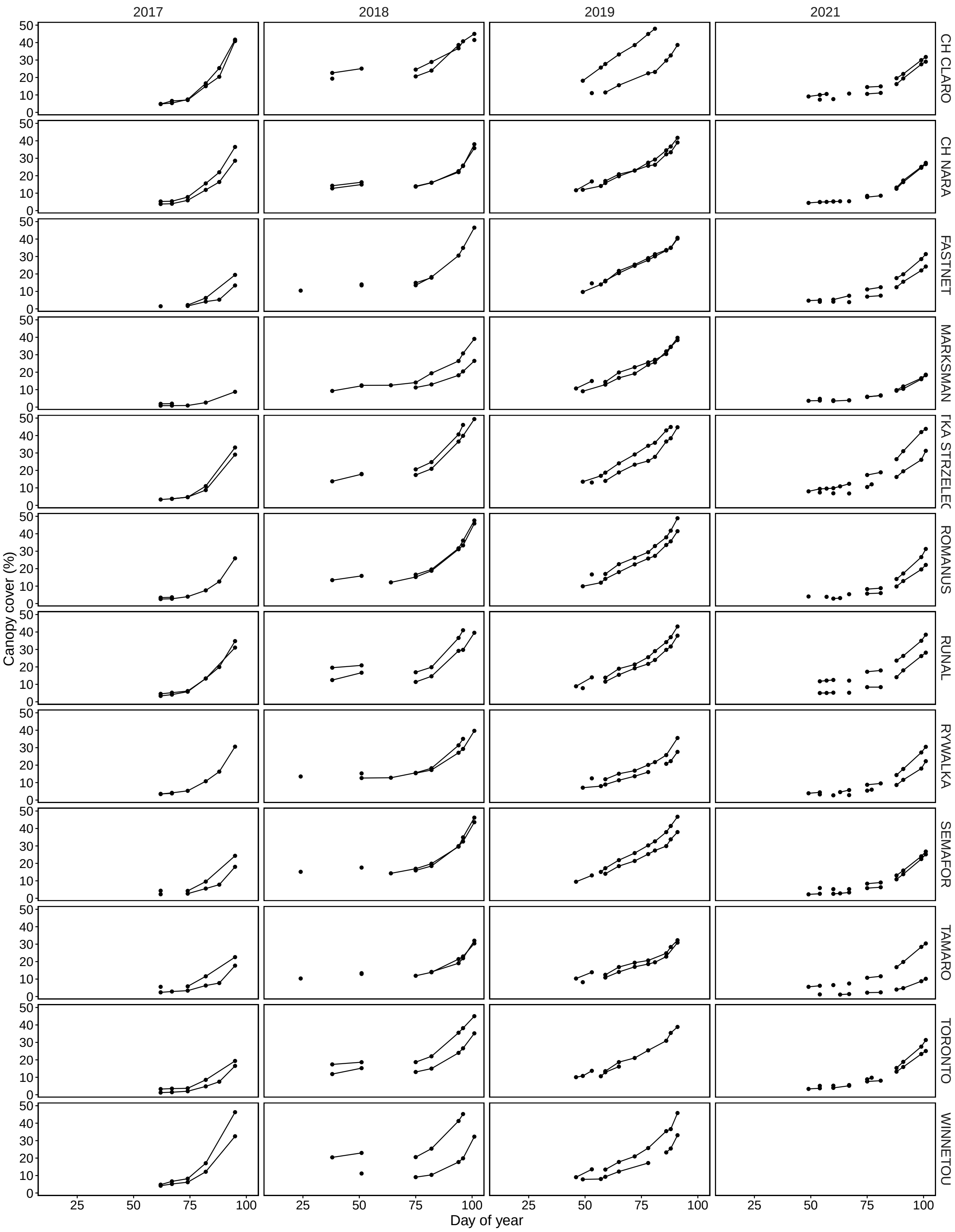

Figure B.1: Canopy cover measurements in wheat with the FIP (FIP RGB)

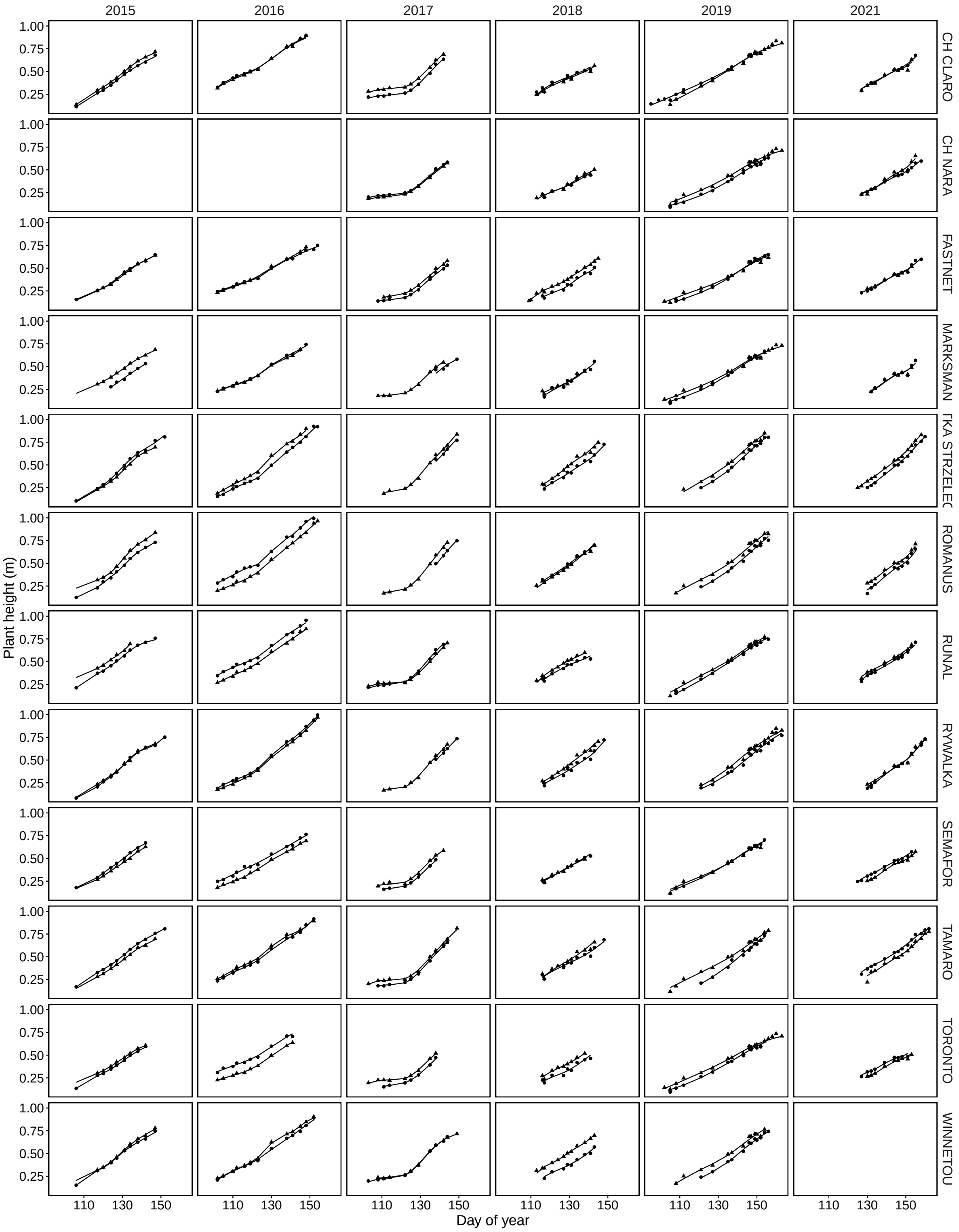

Figure B.2: Plant height measurements in wheat with the FIP (FIP TLS, 2015–2017) and drones (UAV SfM, 2018–2021), smoothed with a P-spline.

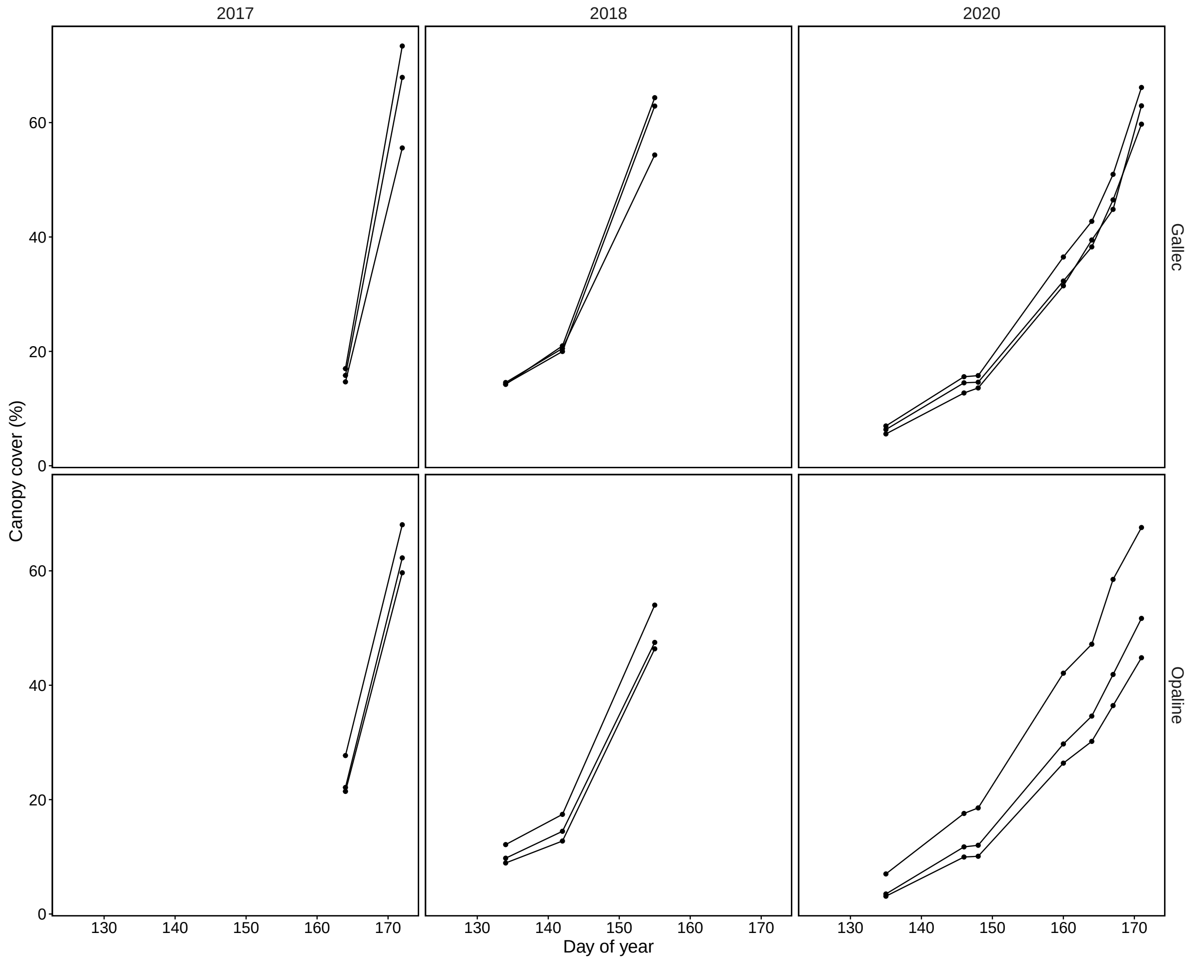

Figure B.3: Canopy cover measurements in soybean with the FIP (FIP RGB)

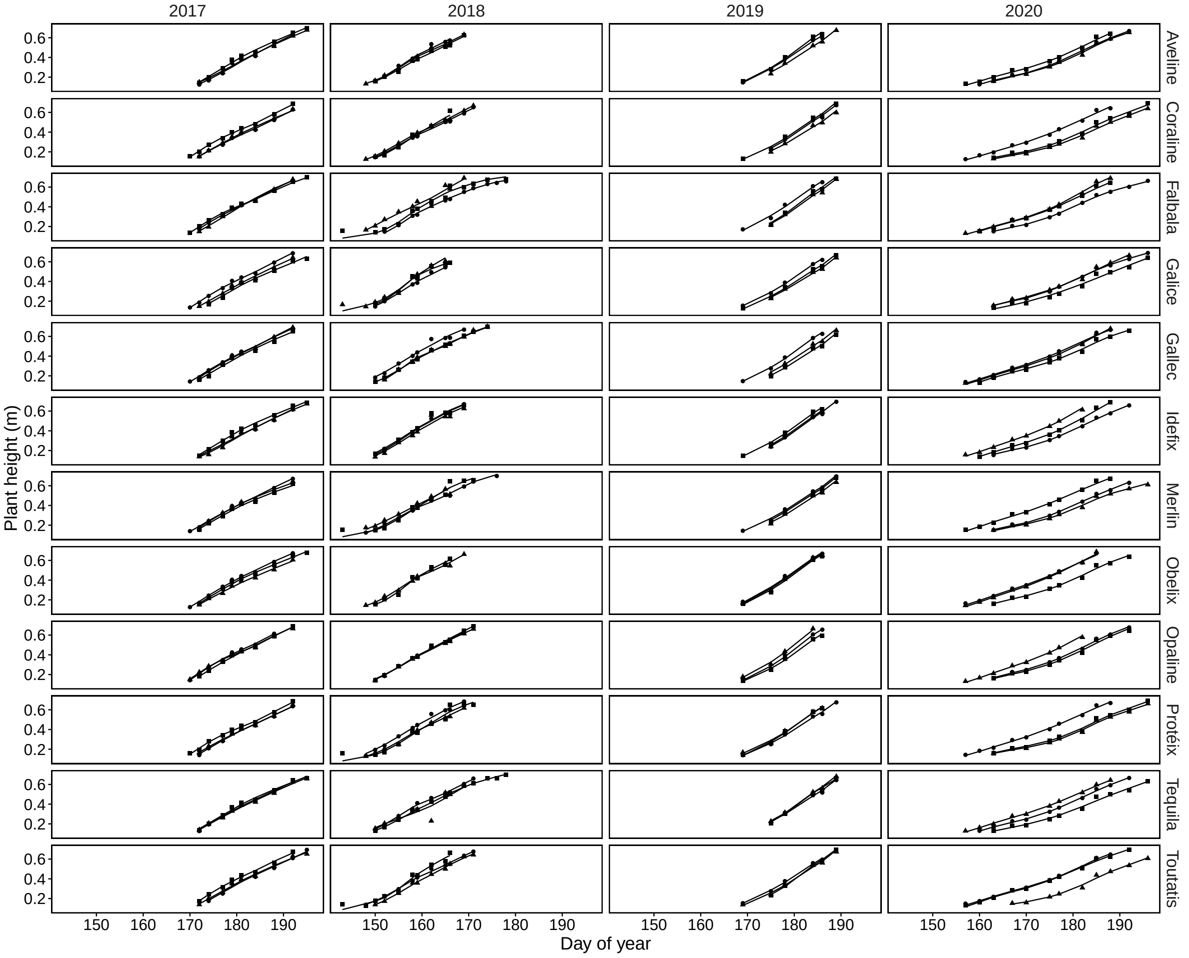

Figure B.4: Plant height measurements in soybean with drones (UAV SfM, 2017–2020), smoothed with a P-spline.

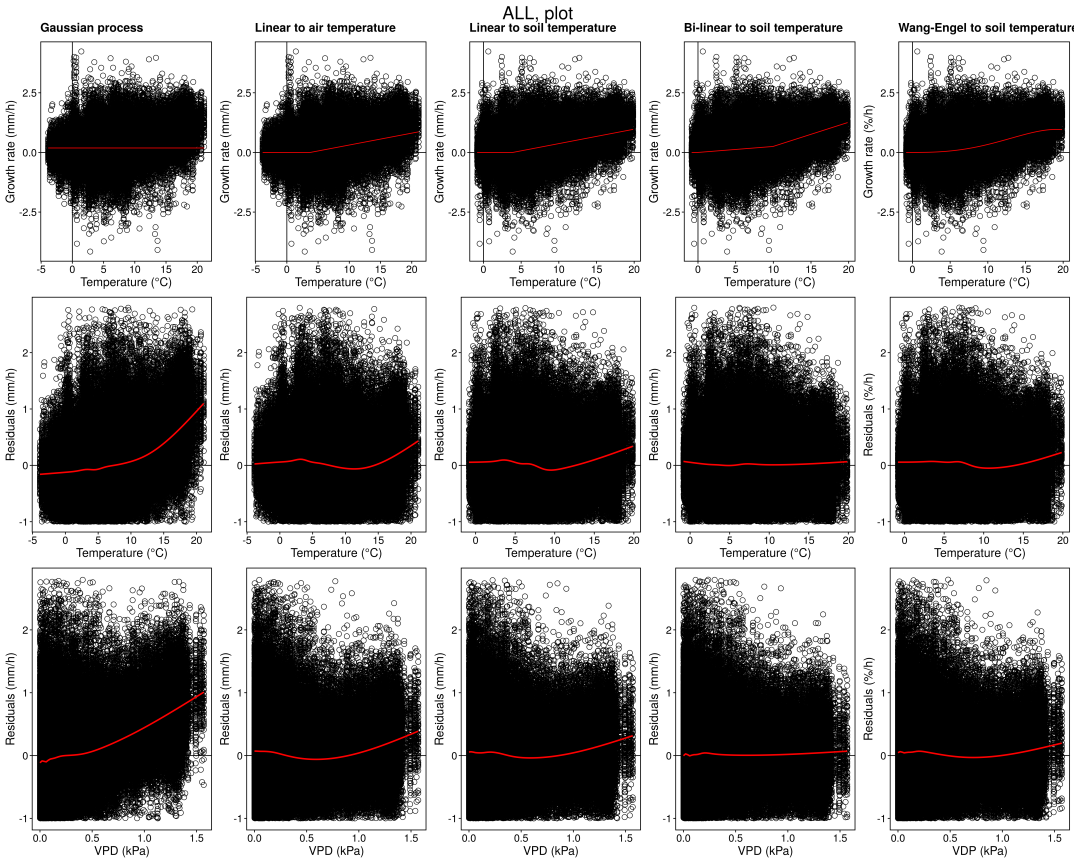

Figure B.5: Leaf length measurements in wheat with the LLT, all 12 genotypes, selected parametric models.

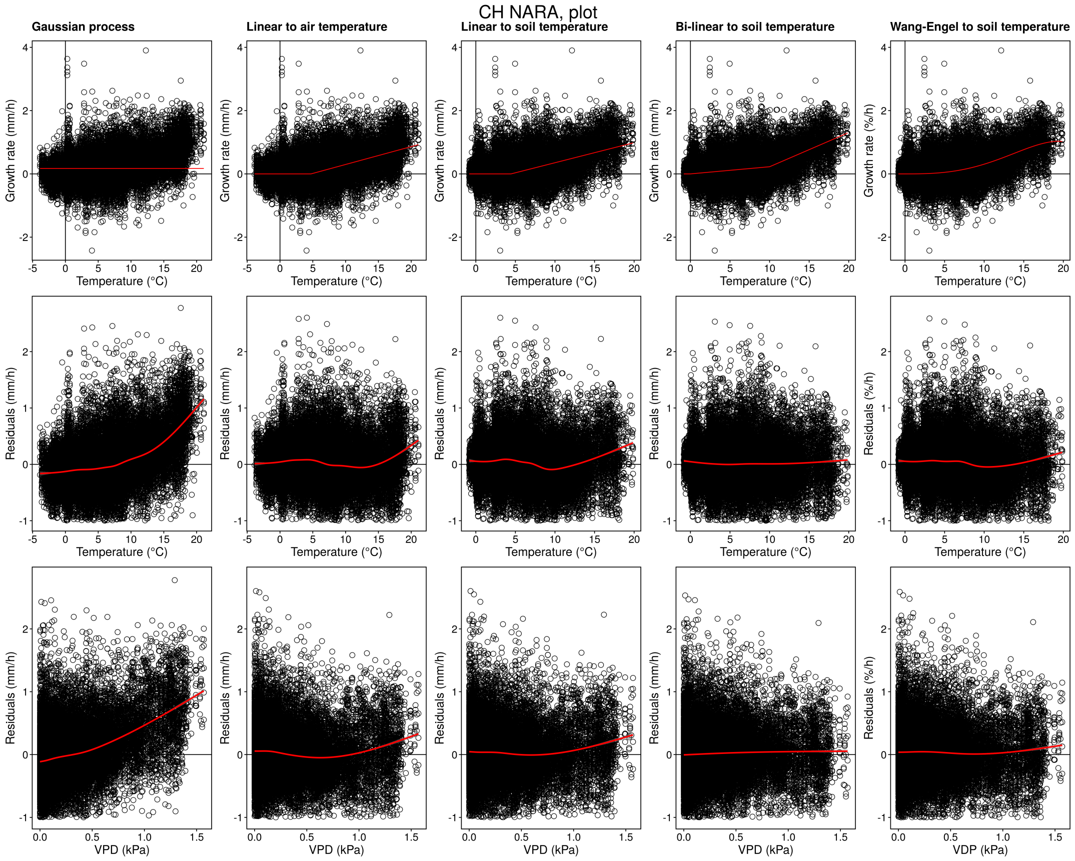

Figure B.6: Leaf length measurements in wheat with the LLT, selected genotype (CH Nara), selected parametric models.

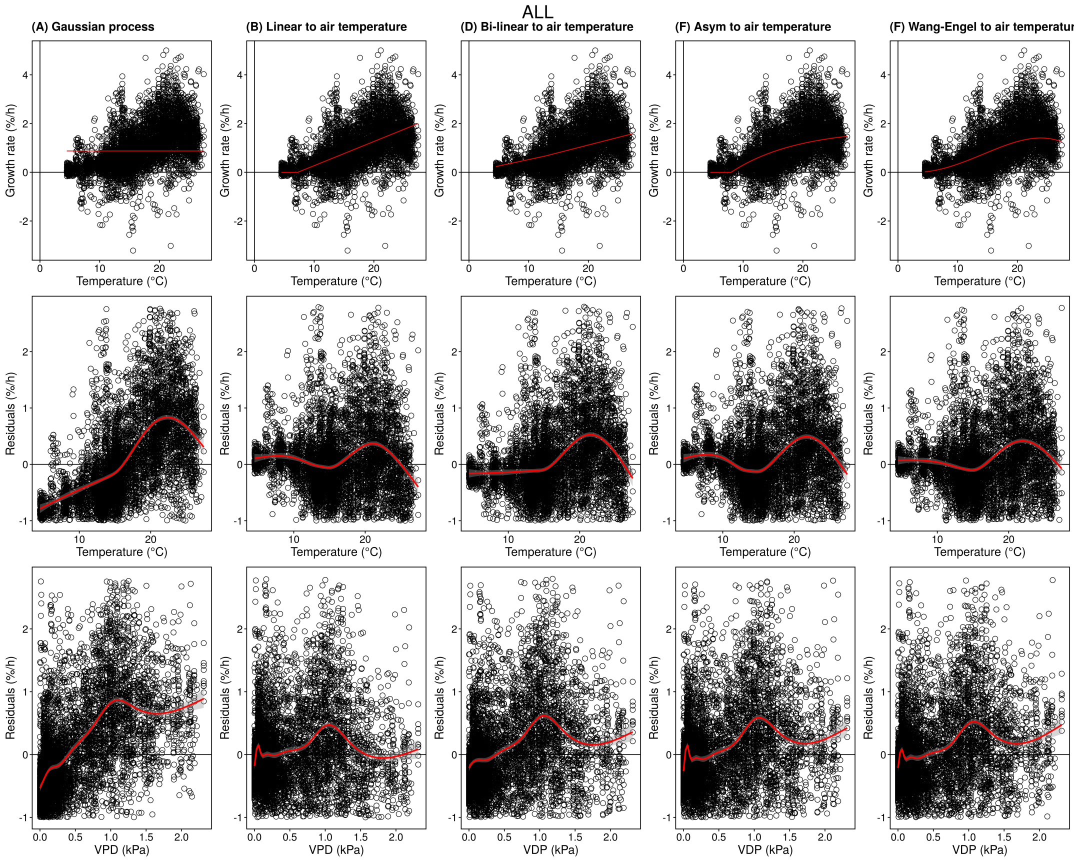

Figure B.7: Leaf growth measurements in soybean with MARTRACK, all 3 genotypes, selected parametric models.

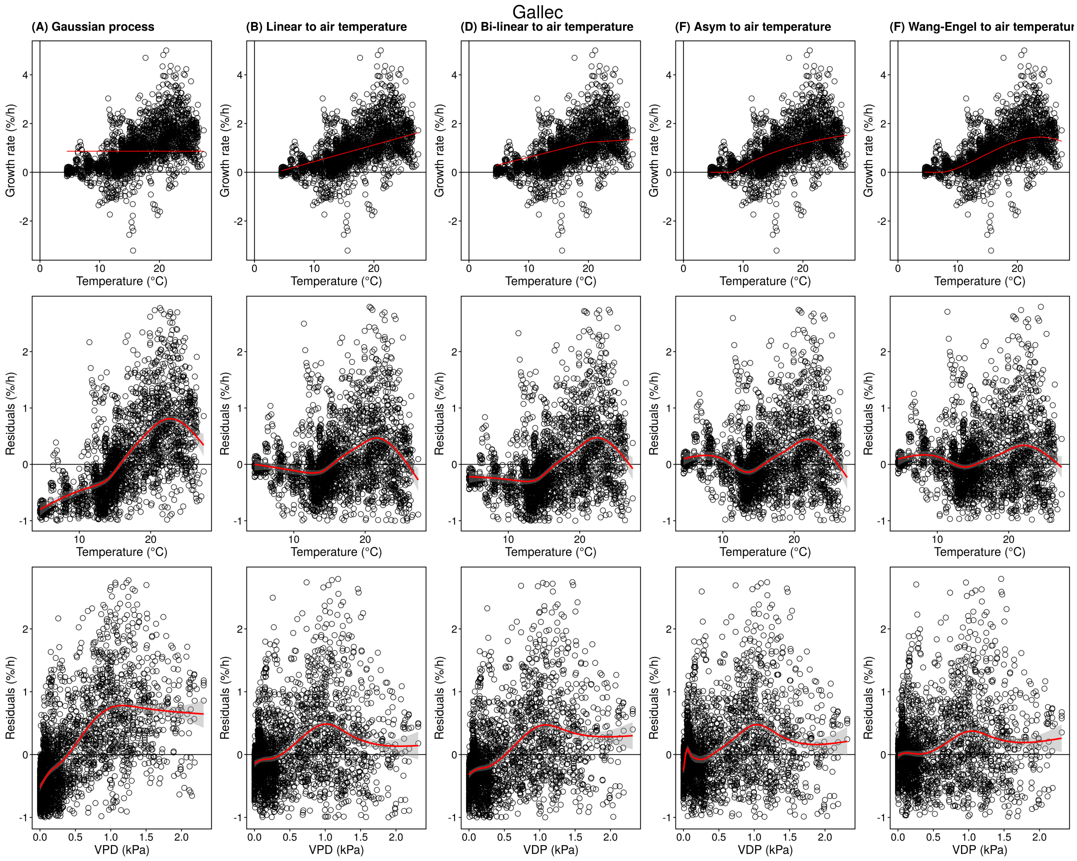

Figure B.8: Leaf growth measurements in soybean with MARTRACK, selected genotype (Gallec), selected parametric models.

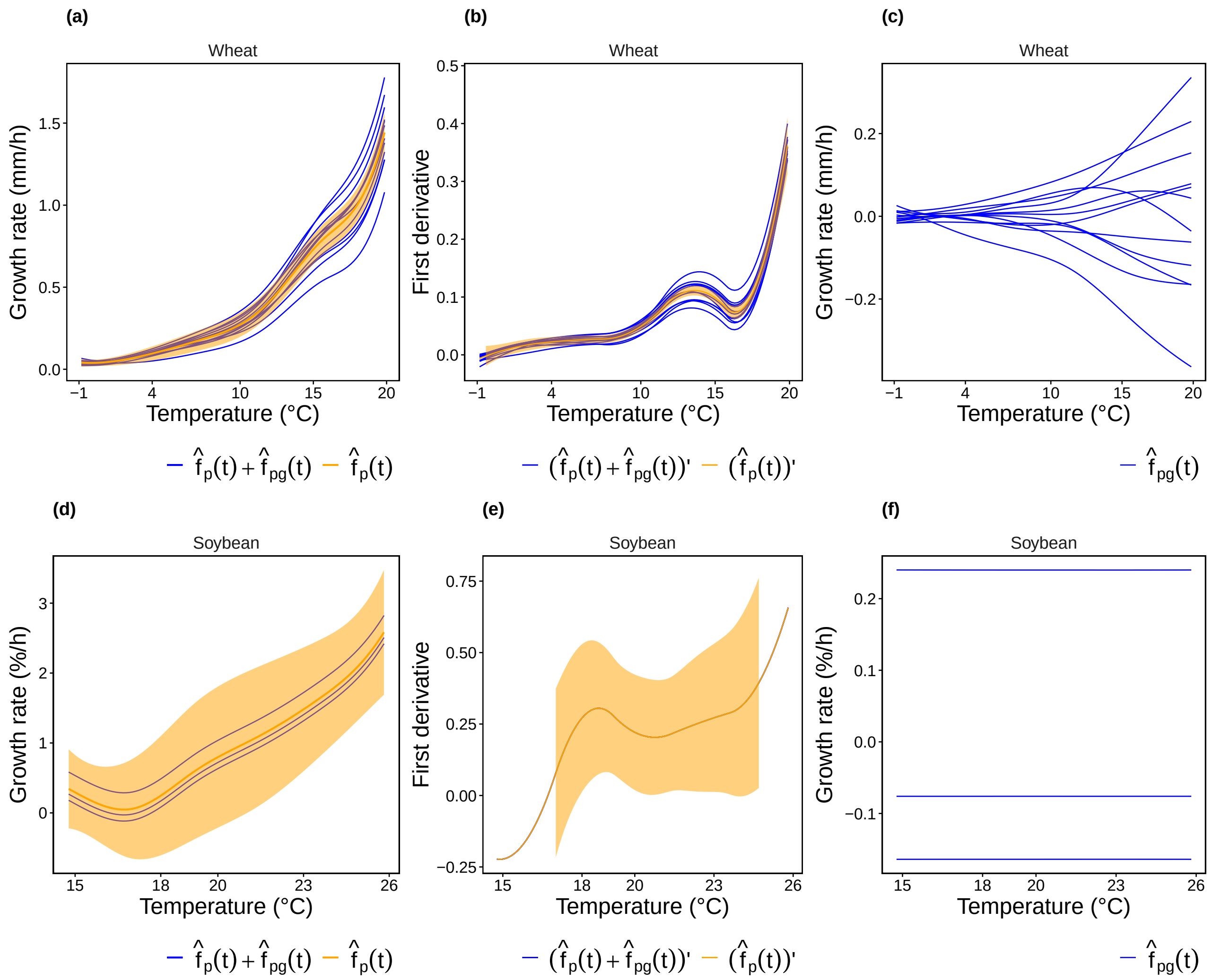

Figure B.9: Fitted hierarchical splines for leaf length tracker (LLT) measurements in wheat and leaf growth tracker (MARTRACK) measurements in soybean.

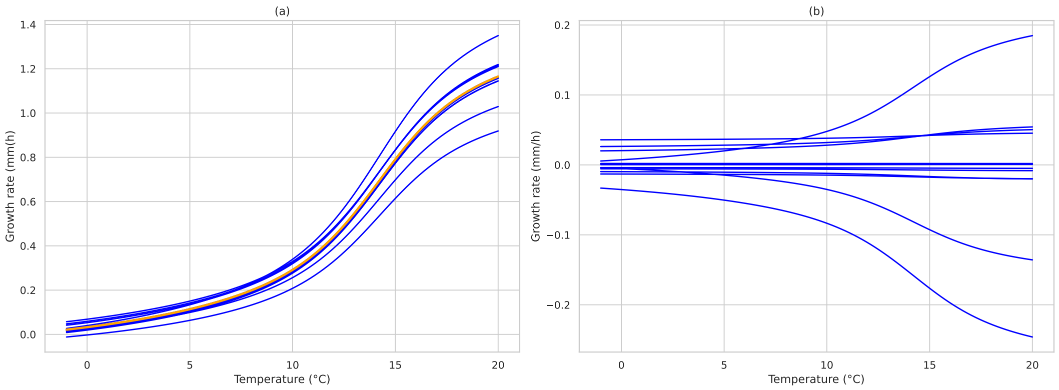

Figure B.10: Fitted neural network model for leaf length tracker (LLT) measurements in wheat, regression to plot soil temperature.

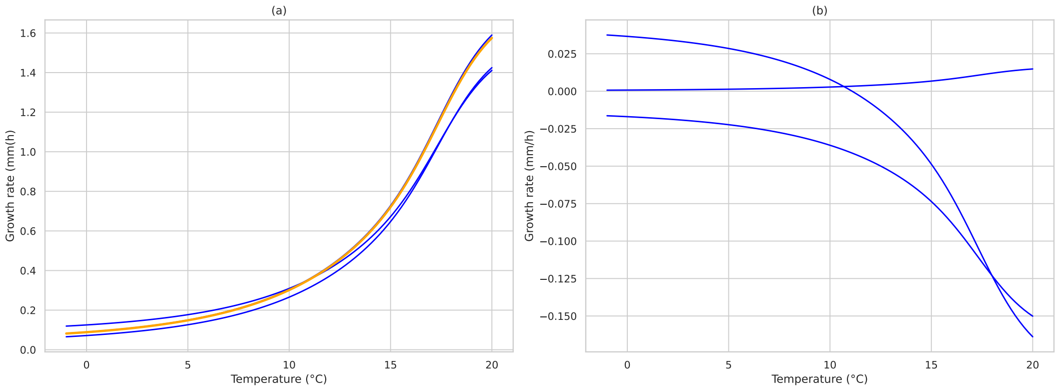

Figure B.11: Fitted neural network model for leaf growth tracker (MARTRACK) measurements in soybean, regression to reference air temperature.

Table B.1: Environmental indices and categories.

	Environmental variable	Daily	Growth period	Category
pr_mean	Precipitation	Sum	Mean	Precipitation
pr_max	Precipitation	Sum	Maximum	
pr_cum	Precipitation	Sum	Sum	
tas_mean	Temp.	Mean	Mean	Mean temp.
tas_min	Temp.	Mean	Minimum	
tas_max	Temp.	Mean	Maximum	
tas_cum	Temp.	Mean	Sum	
tasmin_mean	Temp.	Minumum	Mean	Min temp.
tasmin_min	Temp.	Minumum	Minumum	
tasmin_cum	Temp.	Minumum	Sum	
tasmax_mean	Temp.	Maximum	Mean	Max temp.
tasmax_max	Temp.	Maximum	Max	
tasmax_cum	Temp.	Maximum	Sum	
global_radiation_mean	Global radiation	Sum	Mean	Global radiation
global_radiation_min	Global radiation	Sum	Min	
global_radiation_max	Global radiation	Sum	Maximum	
global_radiation_cum	Global radiation	Sum	Sum	
SPI_mean	Precipitation/Temp.	Sum/Mean	Mean	Drought / Moist
SPI_min	Precipitation/Temp.	Sum/Mean	Min	
SPI_max	Precipitation/Temp.	Sum/Mean	Maximum	
SPI_cum	Precipitation/Temp.	Sum/Mean	Sum	
SPEI_mean	Precipitation/Temp.	Sum/Mean	Mean	
SPEI_min	Precipitation/Temp.	Sum/Mean	Min	
SPEI_max	Precipitation/Temp.	Sum/Mean	Maximum	
SPEI_cum	Precipitation/Temp.	Sum/Mean	Sum	
tasmin_sum_below_0	Temp.	Minimum	Sum(Abs) if < 0 °C	Frost

Table B.2: Fitted non-linear models to leaf length tracker (LLT) wheat data. Genotype=ALL denotes the species-specific models.

genotype_name	model	covariate_suffix	AIC	BIC	RMSE	Tmin	a	rmin	lrc	Asym	Topt	Tmax	rmax	mu	sigma	rho_1	rho_2	rho_3
ALL	Asym response to air temperature	^{plot}	342621.60	342686.35	0.41	4.29			0.71	1.00					0.39	0.11	0.15	0.15
ALL	Asym response to air temperature	^{ref}	342846.90	342911.71	0.41	3.60			0.67	1.00					0.39	0.11	0.15	0.15
ALL	Asym response to soil temperature	^{plot}	339503.20	339567.95	0.40	4.41			0.94	1.00					0.39	0.10	0.14	0.14
ALL	Asym response to soil temperature	^{ref}	343053.00	343117.80	0.41	5.83			1.53	1.00					0.39	0.11	0.16	0.15
ALL	Bi-linear response to air temperature	^{plot}	336613.10	336688.64	0.40	12.57	0.10	0.31							0.39	0.09	0.13	0.13
ALL	Bi-linear response to air temperature	^{ref}	336879.20	336954.71	0.40	12.31	0.11	0.32							0.39	0.09	0.13	0.13
ALL	Bi-linear response to soil temperature	^{plot}	331876.10	331951.69	0.39	9.95	0.10	0.25							0.38	0.07	0.12	0.12
ALL	Bi-linear response to soil temperature	^{ref}	338460.70	338536.25	0.40	7.83	0.17	0.15							0.39	0.10	0.14	0.14
ALL	Gaussian process	^{plot}	358273.10	358327.04	0.44									0.19	0.40	0.16	0.21	0.20
ALL	Gaussian process	^{ref}	358322.90	358376.85	0.44									0.19	0.40	0.16	0.21	0.20
ALL	Linear response to air temperature	^{plot}	340399.10	340463.87	0.40	3.75	0.05								0.39	0.10	0.15	0.14
ALL	Linear response to air temperature	^{ref}	340626.70	340691.42	0.40	3.22	0.05								0.39	0.10	0.15	0.14
ALL	Linear response to soil temperature	^{plot}	336532.40	336597.13	0.40	3.88	0.06								0.39	0.09	0.13	0.13
ALL	Linear response to soil temperature	^{ref}	341125.00	341189.79	0.40	5.53	0.11								0.39	0.10	0.15	0.15
ALL	Thermal time to air temperature	^{plot}	342177.50	342231.49	0.41	0.00	0.03								0.39	0.11	0.15	0.15
ALL	Thermal time to air temperature	^{ref}	341963.90	342017.86	0.40	0.00	0.03								0.39	0.11	0.15	0.15
ALL	Thermal time to soil temperature	^{plot}	340023.80	340077.79	0.40	0.00	0.04								0.39	0.10	0.15	0.14
ALL	Thermal time to soil temperature	^{ref}	350097.90	350151.91	0.42	0.00	0.03								0.39	0.13	0.18	0.17
ALL	Wang-Engel response to air temperature	^{plot}	339730.40	339795.18	0.40	0.00					19.19	25.00	0.81		0.39	0.10	0.14	0.14
ALL	Wang-Engel response to air temperature	^{ref}	340008.70	340073.46	0.40	0.00					18.94	25.00	0.81		0.39	0.10	0.14	0.14
ALL	Wang-Engel response to soil temperature	^{plot}	334542.50	334607.28	0.40	0.00					19.07	25.00	0.96		0.39	0.08	0.13	0.12
ALL	Wang-Engel response to soil temperature	^{ref}	338024.40	338089.21	0.40	0.00					20.40	25.00	2.88		0.39	0.09	0.14	0.14
CH CLARO	Asym response to air temperature	^{plot}	29564.00	29613.63	0.44	3.20			0.78	1.00					0.41	0.18	0.17	0.15
CH CLARO	Asym response to air temperature	^{ref}	29581.70	29631.28	0.44	3.71			0.93	1.00					0.41	0.18	0.17	0.15
CH CLARO	Asym response to soil temperature	^{plot}	29252.00	29301.61	0.43	4.53			1.21	1.00					0.40	0.17	0.16	0.14
CH CLARO	Asym response to soil temperature	^{ref}	29385.80	29435.39	0.43	6.04			1.83	1.00					0.40	0.18	0.17	0.15
CH CLARO	Bi-linear response to air temperature	^{plot}	29128.20	29186.10	0.43	10.65	0.09	0.31							0.40	0.17	0.16	0.14
CH CLARO	Bi-linear response to air temperature	^{ref}	29169.00	29226.88	0.43	10.09	0.09	0.31							0.40	0.17	0.16	0.14
CH CLARO	Bi-linear response to soil temperature	^{plot}	28751.30	28809.18	0.42	9.64	0.11	0.30							0.40	0.15	0.14	0.13
CH CLARO	Bi-linear response to soil temperature	^{ref}	28978.10	29036.01	0.42	7.81	0.20	0.17							0.40	0.16	0.15	0.14
CH CLARO	Gaussian process	^{plot}	30612.30	30653.67	0.48									0.23	0.41	0.23	0.22	0.20
CH CLARO	Gaussian process	^{ref}	30616.60	30657.93	0.48									0.23	0.41	0.23	0.22	0.20
CH CLARO	Linear response to air temperature	^{plot}	29363.80	29413.39	0.43	2.35	0.05								0.40	0.18	0.16	0.15
CH CLARO	Linear response to air temperature	^{ref}	29391.00	29440.58	0.43	1.85	0.05								0.40	0.18	0.16	0.15
CH CLARO	Linear response to soil temperature	^{plot}	29006.40	29055.99	0.42	3.17	0.07								0.40	0.16	0.15	0.13
CH CLARO	Linear response to soil temperature	^{ref}	29186.40	29236.02	0.43	5.85	0.14								0.40	0.17	0.16	0.14
CH CLARO	Thermal time to air temperature	^{plot}	29435.10	29476.42	0.43	0.00	0.04								0.40	0.18	0.17	0.15
CH CLARO	Thermal time to air temperature	^{ref}	29436.40	29477.77	0.43	0.00	0.04								0.40	0.18	0.17	0.15
CH CLARO	Thermal time to soil temperature	^{plot}	29228.80	29270.16	0.43	0.00	0.04								0.40	0.17	0.16	0.14
CH CLARO	Thermal time to soil temperature	^{ref}	29985.00	30026.33	0.45	0.00	0.04								0.41	0.20	0.19	0.17
CH CLARO	Wang-Engel response to air temperature	^{plot}	29340.40	29390.00	0.43	0.00					18.85	25.00	0.91		0.40	0.17	0.16	0.14
CH CLARO	Wang-Engel response to air temperature	^{ref}	29374.10	29423.69	0.43	0.00					18.57	25.00	0.90		0.40	0.17	0.16	0.15
CH CLARO	Wang-Engel response to soil temperature	^{plot}	28900.70	28950.26	0.42	0.00					18.73	25.00	1.06		0.40	0.16	0.15	0.13
CH CLARO	Wang-Engel response to soil temperature	^{ref}	28972.10	29021.71	0.42	0.00					20.44	25.00	3.48		0.40	0.16	0.15	0.14
CH NARA	Asym response to air temperature	^{plot}	27009.60	27060.54	0.36	5.82			0.97	1.00					0.35	0.09	0.15	0.14
CH NARA	Asym response to air temperature	^{ref}	27049.70	27100.65	0.36	5.21			0.93	1.00					0.35	0.09	0.15	0.14
CH NARA	Asym response to soil temperature	^{plot}	27210.30	27261.28	0.37	4.78			0.94	1.00					0.35	0.09	0.16	0.15
CH NARA	Asym response to soil temperature	^{ref}	27924.40	27975.33	0.38	5.28			1.24	1.00					0.36	0.12	0.18	0.18

CH NARA	Bi-linear response to air temperature	^{plot}	26324.70	26384.19	0.36	10.80	0.09	0.23							0.35	0.07	0.13	0.12
CH NARA	Bi-linear response to air temperature	^{ref}	26364.20	26423.66	0.36	10.56	0.09	0.24							0.35	0.06	0.13	0.12
CH NARA	Bi-linear response to soil temperature	^{plot}	26228.20	26287.64	0.36	10.01	0.11	0.23							0.35	0.06	0.12	0.12
CH NARA	Bi-linear response to soil temperature	^{ref}	27516.20	27575.65	0.37	8.73	0.20	0.18							0.35	0.10	0.17	0.16
CH NARA	Gaussian process	^{plot}	29118.60	29161.09	0.40									0.17	0.36	0.16	0.22	0.21
CH NARA	Gaussian process	^{ref}	29126.00	29168.44	0.40									0.17	0.36	0.16	0.22	0.21
CH NARA	Linear response to air temperature	^{plot}	26713.60	26764.59	0.36	4.73	0.06								0.35	0.08	0.14	0.13
CH NARA	Linear response to air temperature	^{ref}	26761.80	26812.78	0.36	4.44	0.06								0.35	0.08	0.14	0.13
CH NARA	Linear response to soil temperature	^{plot}	26858.80	26909.79	0.36	4.45	0.06								0.35	0.08	0.14	0.14
CH NARA	Linear response to soil temperature	^{ref}	27773.80	27824.77	0.38	5.13	0.09								0.36	0.11	0.18	0.17
CH NARA	Thermal time to air temperature	^{plot}	27190.00	27232.50	0.37	0.00	0.03								0.35	0.09	0.16	0.15
CH NARA	Thermal time to air temperature	^{ref}	27167.40	27209.84	0.37	0.00	0.03								0.35	0.09	0.15	0.15
CH NARA	Thermal time to soil temperature	^{plot}	27332.40	27374.84	0.37	0.00	0.03								0.35	0.10	0.16	0.16
CH NARA	Thermal time to soil temperature	^{ref}	28421.80	28464.25	0.39	0.00	0.03								0.36	0.13	0.20	0.19
CH NARA	Wang-Engel response to air temperature	^{plot}	26581.10	26632.10	0.36	0.00					19.48	25.00	0.86		0.35	0.07	0.13	0.13
CH NARA	Wang-Engel response to air temperature	^{ref}	26616.50	26667.48	0.36	0.00					19.28	25.00	0.87		0.35	0.07	0.13	0.13
CH NARA	Wang-Engel response to soil temperature	^{plot}	26538.00	26588.91	0.36	0.00					19.42	25.00	1.03		0.35	0.07	0.13	0.13
CH NARA	Wang-Engel response to soil temperature	^{ref}	27495.30	27546.28	0.37	0.00					20.32	25.00	2.52		0.35	0.10	0.17	0.16
FASTNET	Asym response to air temperature	^{plot}	28372.00	28420.77	0.43	4.98			0.69	1.00					0.43	-0.02	0.12	0.12
FASTNET	Asym response to air temperature	^{ref}	28397.30	28446.11	0.43	4.40			0.67	1.00					0.43	-0.02	0.12	0.12
FASTNET	Asym response to soil temperature	^{plot}	28131.60	28180.45	0.43	4.11			0.76	1.00					0.42	-0.03	0.11	0.11
FASTNET	Asym response to soil temperature	^{ref}	28364.20	28413.01	0.43	5.18			1.28	1.00					0.43	-0.02	0.12	0.12
FASTNET	Bi-linear response to air temperature	^{plot}	27954.10	28011.06	0.43	10.08	0.07	0.20							0.42	-0.04	0.10	0.10
FASTNET	Bi-linear response to air temperature	^{ref}	27974.50	28031.48	0.43	9.59	0.07	0.20							0.42	-0.04	0.10	0.10
FASTNET	Bi-linear response to soil temperature	^{plot}	27676.10	27733.02	0.42	9.65	0.08	0.23							0.42	-0.05	0.08	0.09
FASTNET	Bi-linear response to soil temperature	^{ref}	28172.30	28229.24	0.43	6.36	0.10	0.09							0.42	-0.03	0.11	0.12
FASTNET	Gaussian process	^{plot}	29772.10	29812.81	0.46									0.18	0.44	0.04	0.19	0.19
FASTNET	Gaussian process	^{ref}	29776.80	29817.50	0.46									0.18	0.44	0.04	0.19	0.19
FASTNET	Linear response to air temperature	^{plot}	28232.10	28280.93	0.43	4.35	0.05								0.42	-0.03	0.11	0.12
FASTNET	Linear response to air temperature	^{ref}	28256.00	28304.79	0.43	3.86	0.05								0.42	-0.03	0.11	0.11
FASTNET	Linear response to soil temperature	^{plot}	27949.20	27997.96	0.43	2.90	0.05								0.42	-0.04	0.10	0.11
FASTNET	Linear response to soil temperature	^{ref}	28246.00	28294.86	0.43	4.89	0.09								0.42	-0.03	0.11	0.12
FASTNET	Thermal time to air temperature	^{plot}	28387.60	28428.25	0.43	0.00	0.03								0.43	-0.02	0.12	0.12
FASTNET	Thermal time to air temperature	^{ref}	28369.70	28410.33	0.43	0.00	0.03								0.43	-0.02	0.12	0.12
FASTNET	Thermal time to soil temperature	^{plot}	28172.90	28213.57	0.43	0.00	0.03								0.42	-0.03	0.11	0.12
FASTNET	Thermal time to soil temperature	^{ref}	28977.60	29018.27	0.44	0.00	0.03								0.43	0.01	0.15	0.15
FASTNET	Wang-Engel response to air temperature	^{plot}	28165.70	28214.56	0.43	0.00					19.11	25.00	0.71		0.42	-0.03	0.11	0.11
FASTNET	Wang-Engel response to air temperature	^{ref}	28183.10	28231.88	0.43	0.00					18.90	25.00	0.72		0.42	-0.03	0.11	0.11
FASTNET	Wang-Engel response to soil temperature	^{plot}	27869.30	27918.12	0.43	0.00					18.75	25.00	0.80		0.42	-0.04	0.09	0.10
FASTNET	Wang-Engel response to soil temperature	^{ref}	28111.00	28159.82	0.43	0.00					19.84	25.00	1.90		0.42	-0.03	0.11	0.11
MARKSMAN	Asym response to air temperature	^{plot}	27898.50	27949.39	0.37	4.76			0.82	1.00					0.36	0.03	0.18	0.19
MARKSMAN	Asym response to air temperature	^{ref}	27940.40	27991.27	0.37	3.59			0.70	1.00					0.36	0.03	0.18	0.19
MARKSMAN	Asym response to soil temperature	^{plot}	27340.40	27391.31	0.37	4.38			0.98	1.00					0.36	0.01	0.17	0.18
MARKSMAN	Asym response to soil temperature	^{ref}	27822.90	27873.75	0.37	5.78			1.57	1.00					0.36	0.03	0.19	0.19
MARKSMAN	Bi-linear response to air temperature	^{plot}	26961.60	27020.99	0.36	10.98	0.09	0.26							0.35	-0.00	0.15	0.16
MARKSMAN	Bi-linear response to air temperature	^{ref}	27030.70	27090.11	0.36	10.57	0.09	0.26							0.35	-0.00	0.15	0.16
MARKSMAN	Bi-linear response to soil temperature	^{plot}	26188.00	26247.40	0.36	9.93	0.11	0.26							0.35	-0.02	0.13	0.14
MARKSMAN	Bi-linear response to soil temperature	^{ref}	27276.00	27335.32	0.37	7.67	0.17	0.15							0.36	0.01	0.17	0.17
MARKSMAN	Gaussian process	^{plot}	29711.80	29754.26	0.41									0.19	0.37	0.09	0.26	0.25
MARKSMAN	Gaussian process	^{ref}	29718.30	29760.74	0.41									0.19	0.37	0.09	0.26	0.25
MARKSMAN	Linear response to air temperature	^{plot}	27573.90	27624.79	0.37	3.55	0.05								0.36	0.02	0.17	0.18
MARKSMAN	Linear response to air temperature	^{ref}	27611.60	27662.46	0.37	3.28	0.05								0.36	0.02	0.17	0.18

MARKSMAN	Linear response to soil temperature	^{plot}	26896.10	26946.96	0.36	4.01	0.06								0.35	-0.00	0.15	0.16
MARKSMAN	Linear response to soil temperature	^{ref}	27550.10	27600.98	0.37	5.45	0.11								0.36	0.02	0.18	0.18
MARKSMAN	Thermal time to air temperature	^{plot}	27816.10	27858.55	0.37	0.00	0.03								0.36	0.03	0.18	0.19
MARKSMAN	Thermal time to air temperature	^{ref}	27797.10	27839.48	0.37	0.00	0.03								0.36	0.03	0.18	0.18
MARKSMAN	Thermal time to soil temperature	^{plot}	27405.50	27447.89	0.37	0.00	0.04								0.36	0.02	0.17	0.18
MARKSMAN	Thermal time to soil temperature	^{ref}	28753.60	28796.01	0.39	0.00	0.03								0.36	0.06	0.22	0.22
MARKSMAN	Wang-Engel response to air temperature	^{plot}	27417.90	27468.78	0.37	0.00					19.34	25.00	0.86		0.36	0.01	0.16	0.17
MARKSMAN	Wang-Engel response to air temperature	^{ref}	27459.90	27510.80	0.37	0.00					19.09	25.00	0.86		0.36	0.01	0.17	0.17
MARKSMAN	Wang-Engel response to soil temperature	^{plot}	26586.30	26637.21	0.36	0.00					19.06	25.00	1.00		0.35	-0.01	0.14	0.15
MARKSMAN	Wang-Engel response to soil temperature	^{ref}	27166.90	27217.80	0.37	0.00					20.37	25.00	2.94		0.35	0.01	0.16	0.17
OSTKA STRZELECKA	Asym response to air temperature	^{plot}	36334.70	36385.16	0.42	2.21			0.14	1.00					0.42	-0.01	0.08	0.10
OSTKA STRZELECKA	Asym response to air temperature	^{ref}	36327.50	36377.96	0.42	1.37			0.09	1.00					0.42	-0.01	0.08	0.10
OSTKA STRZELECKA	Asym response to soil temperature	^{plot}	35746.60	35797.14	0.42	2.63			0.39	1.00					0.41	-0.03	0.06	0.08
OSTKA STRZELECKA	Asym response to soil temperature	^{ref}	35741.80	35792.35	0.42	5.10			1.10	1.00					0.41	-0.03	0.06	0.08
OSTKA STRZELECKA	Bi-linear response to air temperature	^{plot}	35715.20	35774.10	0.42	13.60	0.10	0.28							0.41	-0.03	0.06	0.08
OSTKA STRZELECKA	Bi-linear response to air temperature	^{ref}	35725.80	35784.68	0.42	13.22	0.09	0.29							0.41	-0.03	0.06	0.08
OSTKA STRZELECKA	Bi-linear response to soil temperature	^{plot}	34994.00	35052.89	0.41	10.65	0.08	0.24							0.41	-0.06	0.04	0.06
OSTKA STRZELECKA	Bi-linear response to soil temperature	^{ref}	35325.60	35384.53	0.41	7.42	0.12	0.12							0.41	-0.05	0.05	0.07
OSTKA STRZELECKA	Gaussian process	^{plot}	37807.40	37849.54	0.44									0.16	0.43	0.04	0.14	0.15
OSTKA STRZELECKA	Gaussian process	^{ref}	37811.80	37853.85	0.44									0.16	0.43	0.04	0.14	0.15
OSTKA STRZELECKA	Linear response to air temperature	^{plot}	36173.30	36223.77	0.42	1.72	0.03								0.42	-0.02	0.08	0.10
OSTKA STRZELECKA	Linear response to air temperature	^{ref}	36166.40	36216.89	0.42	1.12	0.03								0.42	-0.02	0.08	0.10
OSTKA STRZELECKA	Linear response to soil temperature	^{plot}	35502.40	35552.94	0.41	2.23	0.04								0.41	-0.04	0.05	0.08
OSTKA STRZELECKA	Linear response to soil temperature	^{ref}	35538.70	35589.24	0.41	4.92	0.08								0.41	-0.04	0.06	0.08
OSTKA STRZELECKA	Thermal time to air temperature	^{plot}	36209.90	36251.97	0.42	0.00	0.03								0.42	-0.02	0.08	0.10
OSTKA STRZELECKA	Thermal time to air temperature	^{ref}	36180.40	36222.45	0.42	0.00	0.03								0.42	-0.02	0.08	0.10
OSTKA STRZELECKA	Thermal time to soil temperature	^{plot}	35697.50	35739.56	0.42	0.00	0.03								0.41	-0.03	0.06	0.08
OSTKA STRZELECKA	Thermal time to soil temperature	^{ref}	36650.60	36692.73	0.42	0.00	0.03								0.42	-0.00	0.10	0.11
OSTKA STRZELECKA	Wang-Engel response to air temperature	^{plot}	36199.20	36249.66	0.42	0.00					18.95	25.00	0.60		0.42	-0.02	0.08	0.10
OSTKA STRZELECKA	Wang-Engel response to air temperature	^{ref}	36216.50	36266.97	0.42	0.00					18.68	25.00	0.60		0.42	-0.02	0.08	0.10
OSTKA STRZELECKA	Wang-Engel response to soil temperature	^{plot}	35420.90	35471.42	0.41	0.00					18.77	25.00	0.73		0.41	-0.04	0.05	0.07
OSTKA STRZELECKA	Wang-Engel response to soil temperature	^{ref}	35197.80	35248.31	0.41	0.00					20.13	25.00	2.04		0.41	-0.05	0.04	0.07
ROMANUS	Asym response to air temperature	^{plot}	13478.10	13527.41	0.33	2.55			0.33	1.00					0.31	0.20	0.15	0.15
ROMANUS	Asym response to air temperature	^{ref}	13484.40	13533.74	0.33	2.53			0.39	1.00					0.31	0.20	0.15	0.15
ROMANUS	Asym response to soil temperature	^{plot}	13058.20	13107.58	0.32	4.39			0.82	1.00					0.31	0.19	0.13	0.13
ROMANUS	Asym response to soil temperature	^{ref}	13318.50	13367.81	0.33	6.33			1.58	1.00					0.31	0.20	0.14	0.14
ROMANUS	Bi-linear response to air temperature	^{plot}	13136.30	13193.92	0.32	10.80	0.07	0.23							0.31	0.19	0.13	0.13
ROMANUS	Bi-linear response to air temperature	^{ref}	13146.10	13203.67	0.33	14.24	0.12	0.36							0.31	0.19	0.13	0.13
ROMANUS	Bi-linear response to soil temperature	^{plot}	12667.60	12725.22	0.32	9.35	0.08	0.21							0.30	0.17	0.12	0.11
ROMANUS	Bi-linear response to soil temperature	^{ref}	12929.40	12986.95	0.32	7.67	0.15	0.11							0.31	0.18	0.13	0.13
ROMANUS	Gaussian process	^{plot}	14595.20	14636.34	0.36									0.17	0.32	0.25	0.19	0.19
ROMANUS	Gaussian process	^{ref}	14599.90	14640.99	0.36									0.17	0.32	0.25	0.19	0.19
ROMANUS	Linear response to air temperature	^{plot}	13347.40	13396.76	0.33	2.43	0.04								0.31	0.20	0.14	0.14
ROMANUS	Linear response to air temperature	^{ref}	13362.00	13411.37	0.33	2.11	0.04								0.31	0.20	0.14	0.14
ROMANUS	Linear response to soil temperature	^{plot}	12883.30	12932.66	0.32	3.99	0.06								0.31	0.18	0.13	0.12
ROMANUS	Linear response to soil temperature	^{ref}	13172.50	13221.84	0.33	6.01	0.11								0.31	0.19	0.14	0.14
ROMANUS	Thermal time to air temperature	^{plot}	13431.80	13472.89	0.33	0.00	0.03								0.31	0.20	0.15	0.15
ROMANUS	Thermal time to air temperature	^{ref}	13424.90	13466.05	0.33	0.00	0.03								0.31	0.20	0.15	0.15
ROMANUS	Thermal time to soil temperature	^{plot}	13196.20	13237.29	0.33	0.00	0.03								0.31	0.19	0.14	0.14
ROMANUS	Thermal time to soil temperature	^{ref}	13992.50	14033.66	0.34	0.00	0.03								0.31	0.23	0.17	0.17
ROMANUS	Wang-Engel response to air temperature	^{plot}	13371.80	13421.17	0.33	0.00					18.87	25.00	0.67		0.31	0.20	0.14	0.14
ROMANUS	Wang-Engel response to air temperature	^{ref}	13399.60	13448.95	0.33	0.00					18.56	25.00	0.66		0.31	0.20	0.14	0.14

ROMANUS	Wang-Engel response to soil temperature	^{plot}	12773.10	12822.48	0.32	0.00					18.86	25.00	0.82		0.31	0.18	0.12	0.12
ROMANUS	Wang-Engel response to soil temperature	^{ref}	12904.90	12954.30	0.32	0.00					20.67	25.00	3.16		0.31	0.18	0.13	0.13
RUNAL	Asym response to air temperature	^{plot}	16955.10	17004.05	0.37	3.18			0.65	1.00					0.34	0.25	0.14	0.14
RUNAL	Asym response to air temperature	^{ref}	16961.80	17010.75	0.37	2.31			0.59	1.00					0.34	0.25	0.14	0.14
RUNAL	Asym response to soil temperature	^{plot}	16669.30	16718.34	0.36	3.02			0.85	1.00					0.33	0.23	0.13	0.13
RUNAL	Asym response to soil temperature	^{ref}	16919.00	16968.02	0.36	5.66			1.64	1.00					0.34	0.24	0.14	0.14
RUNAL	Bi-linear response to air temperature	^{plot}	16477.60	16534.71	0.35	13.37	0.13	0.38							0.33	0.23	0.12	0.12
RUNAL	Bi-linear response to air temperature	^{ref}	16499.30	16556.50	0.35	13.19	0.14	0.40							0.33	0.23	0.12	0.12
RUNAL	Bi-linear response to soil temperature	^{plot}	15943.40	16000.58	0.34	10.04	0.12	0.30							0.33	0.20	0.10	0.10
RUNAL	Bi-linear response to soil temperature	^{ref}	16352.50	16409.68	0.35	8.06	0.22	0.18							0.33	0.22	0.12	0.12
RUNAL	Gaussian process	^{plot}	17967.30	18008.16	0.40									0.20	0.34	0.29	0.18	0.19
RUNAL	Gaussian process	^{ref}	17970.80	18011.67	0.40									0.21	0.34	0.29	0.18	0.19
RUNAL	Linear response to air temperature	^{plot}	16774.30	16823.31	0.36	2.51	0.05								0.33	0.24	0.13	0.13
RUNAL	Linear response to air temperature	^{ref}	16788.20	16837.24	0.36	2.12	0.05								0.33	0.24	0.13	0.14
RUNAL	Linear response to soil temperature	^{plot}	16372.10	16421.05	0.35	2.90	0.06								0.33	0.22	0.12	0.12
RUNAL	Linear response to soil temperature	^{ref}	16706.40	16755.40	0.36	5.36	0.12								0.33	0.24	0.13	0.13
RUNAL	Thermal time to air temperature	^{plot}	16861.90	16902.75	0.36	0.00	0.03								0.34	0.24	0.14	0.14
RUNAL	Thermal time to air temperature	^{ref}	16851.60	16892.38	0.36	0.00	0.04								0.33	0.24	0.14	0.14
RUNAL	Thermal time to soil temperature	^{plot}	16600.10	16640.94	0.36	0.00	0.04								0.33	0.23	0.13	0.13
RUNAL	Thermal time to soil temperature	^{ref}	17382.90	17423.70	0.38	0.00	0.04								0.34	0.27	0.16	0.16
RUNAL	Wang-Engel response to air temperature	^{plot}	16773.90	16822.91	0.36	0.00					18.91	25.00	0.85		0.33	0.24	0.13	0.13
RUNAL	Wang-Engel response to air temperature	^{ref}	16807.00	16856.02	0.36	0.00					18.61	25.00	0.84		0.33	0.24	0.13	0.13
RUNAL	Wang-Engel response to soil temperature	^{plot}	16222.10	16271.12	0.35	0.00					18.93	25.00	1.09		0.33	0.22	0.11	0.11
RUNAL	Wang-Engel response to soil temperature	^{ref}	16360.10	16409.07	0.35	0.00					20.48	25.00	3.62		0.33	0.22	0.12	0.12
RYWALKA	Asym response to air temperature	^{plot}	29473.10	29523.82	0.38	5.51			0.40	1.00					0.37	0.02	0.10	0.10
RYWALKA	Asym response to air temperature	^{ref}	29499.90	29550.59	0.38	5.22			0.43	1.00					0.37	0.02	0.10	0.10
RYWALKA	Asym response to soil temperature	^{plot}	29289.10	29339.81	0.37	5.71			0.66	1.00					0.37	0.01	0.10	0.10
RYWALKA	Asym response to soil temperature	^{ref}	29909.40	29960.14	0.38	6.11			1.06	1.00					0.37	0.03	0.12	0.12
RYWALKA	Bi-linear response to air temperature	^{plot}	29213.50	29272.73	0.37	12.49	0.08	0.19							0.37	0.01	0.09	0.10
RYWALKA	Bi-linear response to air temperature	^{ref}	29216.30	29275.47	0.37	12.16	0.08	0.20							0.37	0.01	0.09	0.10
RYWALKA	Bi-linear response to soil temperature	^{plot}	28850.90	28910.07	0.37	9.69	0.07	0.13							0.37	-0.00	0.08	0.09
RYWALKA	Bi-linear response to soil temperature	^{ref}	29623.20	29682.42	0.38	8.04	0.12	0.09							0.37	0.02	0.11	0.11
RYWALKA	Gaussian process	^{plot}	31064.10	31106.34	0.39									0.11	0.38	0.07	0.16	0.16
RYWALKA	Gaussian process	^{ref}	31068.30	31110.56	0.39									0.11	0.38	0.07	0.16	0.16
RYWALKA	Linear response to air temperature	^{plot}	29374.20	29424.95	0.37	5.18	0.04								0.37	0.01	0.10	0.10
RYWALKA	Linear response to air temperature	^{ref}	29401.40	29452.09	0.37	4.62	0.04								0.37	0.01	0.10	0.10
RYWALKA	Linear response to soil temperature	^{plot}	29149.80	29200.49	0.37	5.25	0.05								0.37	0.01	0.09	0.10
RYWALKA	Linear response to soil temperature	^{ref}	29836.10	29886.80	0.38	6.00	0.08								0.37	0.03	0.12	0.12
RYWALKA	Thermal time to air temperature	^{plot}	29727.50	29769.79	0.38	0.00	0.02								0.37	0.03	0.11	0.11
RYWALKA	Thermal time to air temperature	^{ref}	29687.60	29729.84	0.38	0.00	0.02								0.37	0.02	0.11	0.11
RYWALKA	Thermal time to soil temperature	^{plot}	29668.40	29710.72	0.38	0.00	0.02								0.37	0.02	0.11	0.11
RYWALKA	Thermal time to soil temperature	^{ref}	30469.70	30511.96	0.39	0.00	0.02								0.38	0.05	0.14	0.14
RYWALKA	Wang-Engel response to air temperature	^{plot}	29288.90	29339.61	0.37	0.00					19.62	25.00	0.59		0.37	0.01	0.10	0.10
RYWALKA	Wang-Engel response to air temperature	^{ref}	29309.10	29359.85	0.37	0.00					19.38	25.00	0.59		0.37	0.01	0.10	0.10
RYWALKA	Wang-Engel response to soil temperature	^{plot}	28961.90	29012.66	0.37	0.00					19.62	25.00	0.74		0.37	0.00	0.09	0.09
RYWALKA	Wang-Engel response to soil temperature	^{ref}	29614.80	29665.57	0.38	0.00					20.66	25.00	2.21		0.37	0.02	0.11	0.11
SEMAFOR	Asym response to air temperature	^{plot}	21929.10	21977.89	0.40	2.44			0.36	1.00					0.38	0.26	0.09	0.09
SEMAFOR	Asym response to air temperature	^{ref}	21929.00	21977.72	0.40	2.28			0.40	1.00					0.38	0.26	0.09	0.09
SEMAFOR	Asym response to soil temperature	^{plot}	22008.00	22056.79	0.40	3.03			0.55	1.00					0.38	0.27	0.10	0.09
SEMAFOR	Asym response to soil temperature	^{ref}	22297.30	22346.03	0.41	4.91			1.02	1.00					0.38	0.28	0.11	0.11
SEMAFOR	Bi-linear response to air temperature	^{plot}	21691.40	21748.28	0.39	13.68	0.11	0.34							0.37	0.25	0.08	0.08
SEMAFOR	Bi-linear response to air temperature	^{ref}	21691.30	21748.20	0.39	14.07	0.12	0.38							0.37	0.25	0.08	0.08

SEMAFOR	Bi-linear response to soil temperature	^{plot}	21637.70	21694.58	0.39	10.31	0.10	0.24							0.37	0.25	0.08	0.08
SEMAFOR	Bi-linear response to soil temperature	^{ref}	22046.20	22103.07	0.40	8.55	0.17	0.17							0.38	0.27	0.10	0.10
SEMAFOR	Gaussian process	^{plot}	22834.70	22875.32	0.43									0.17	0.38	0.30	0.13	0.13
SEMAFOR	Gaussian process	^{ref}	22836.70	22877.32	0.43									0.17	0.38	0.30	0.13	0.13
SEMAFOR	Linear response to air temperature	^{plot}	21846.40	21895.15	0.40	1.91	0.04								0.37	0.26	0.09	0.08
SEMAFOR	Linear response to air temperature	^{ref}	21847.60	21896.39	0.40	1.40	0.04								0.37	0.26	0.09	0.08
SEMAFOR	Linear response to soil temperature	^{plot}	21893.30	21942.04	0.40	2.92	0.05								0.38	0.26	0.09	0.09
SEMAFOR	Linear response to soil temperature	^{ref}	22235.00	22283.75	0.41	4.74	0.08								0.38	0.28	0.11	0.10
SEMAFOR	Thermal time to air temperature	^{plot}	21888.50	21929.11	0.40	0.00	0.03								0.38	0.26	0.09	0.09
SEMAFOR	Thermal time to air temperature	^{ref}	21874.70	21915.29	0.40	0.00	0.03								0.38	0.26	0.09	0.09
SEMAFOR	Thermal time to soil temperature	^{plot}	21976.10	22016.71	0.40	0.00	0.03								0.38	0.27	0.10	0.09
SEMAFOR	Thermal time to soil temperature	^{ref}	22463.60	22504.25	0.41	0.00	0.03								0.38	0.29	0.11	0.11
SEMAFOR	Wang-Engel response to air temperature	^{plot}	21887.20	21936.01	0.40	0.00					18.49	25.00	0.65		0.38	0.26	0.09	0.08
SEMAFOR	Wang-Engel response to air temperature	^{ref}	21887.20	21935.95	0.40	0.00					18.25	25.00	0.65		0.38	0.26	0.09	0.09
SEMAFOR	Wang-Engel response to soil temperature	^{plot}	21806.50	21855.23	0.40	0.00					19.06	25.00	0.87		0.37	0.26	0.09	0.08
SEMAFOR	Wang-Engel response to soil temperature	^{ref}	22084.30	22133.06	0.40	0.00					20.20	25.00	2.16		0.38	0.27	0.10	0.10
TAMARO	Asym response to air temperature	^{plot}	30921.10	30971.30	0.43	4.83			1.04	1.00					0.39	0.15	0.20	0.18
TAMARO	Asym response to air temperature	^{ref}	30928.80	30979.04	0.43	4.38			1.03	1.00					0.39	0.15	0.20	0.18
TAMARO	Asym response to soil temperature	^{plot}	30568.90	30619.14	0.42	4.99			1.30	1.00					0.39	0.14	0.19	0.17
TAMARO	Asym response to soil temperature	^{ref}	30696.60	30746.85	0.42	6.26			1.95	1.00					0.39	0.14	0.20	0.17
TAMARO	Bi-linear response to air temperature	^{plot}	30101.20	30159.79	0.41	13.00	0.16	0.37							0.39	0.12	0.17	0.15
TAMARO	Bi-linear response to air temperature	^{ref}	30139.50	30198.07	0.41	12.27	0.15	0.37							0.39	0.12	0.17	0.15
TAMARO	Bi-linear response to soil temperature	^{plot}	29513.40	29571.99	0.40	9.99	0.14	0.28							0.38	0.10	0.15	0.13
TAMARO	Bi-linear response to soil temperature	^{ref}	30093.30	30151.86	0.41	8.04	0.24	0.16							0.39	0.12	0.18	0.15
TAMARO	Gaussian process	^{plot}	32152.60	32194.47	0.47									0.22	0.40	0.20	0.25	0.23
TAMARO	Gaussian process	^{ref}	32155.30	32197.16	0.47									0.22	0.40	0.20	0.25	0.23
TAMARO	Linear response to air temperature	^{plot}	30590.50	30640.73	0.42	4.16	0.06								0.39	0.14	0.19	0.17
TAMARO	Linear response to air temperature	^{ref}	30604.40	30654.66	0.42	4.11	0.07								0.39	0.14	0.19	0.17
TAMARO	Linear response to soil temperature	^{plot}	30073.20	30123.45	0.41	4.76	0.09								0.39	0.12	0.17	0.15
TAMARO	Linear response to soil temperature	^{ref}	30358.80	30409.01	0.41	6.10	0.16								0.39	0.13	0.19	0.16
TAMARO	Thermal time to air temperature	^{plot}	30862.60	30904.42	0.43	0.00	0.04								0.39	0.15	0.20	0.18
TAMARO	Thermal time to air temperature	^{ref}	30841.80	30883.60	0.42	0.00	0.04								0.39	0.15	0.20	0.18
TAMARO	Thermal time to soil temperature	^{plot}	30628.70	30670.56	0.42	0.00	0.04								0.39	0.14	0.20	0.17
TAMARO	Thermal time to soil temperature	^{ref}	31511.20	31553.02	0.45	0.00	0.04								0.40	0.18	0.23	0.20
TAMARO	Wang-Engel response to air temperature	^{plot}	30463.10	30513.32	0.41	0.00					19.71	25.00	1.12		0.39	0.13	0.18	0.16
TAMARO	Wang-Engel response to air temperature	^{ref}	30472.60	30522.82	0.42	0.00					19.50	25.00	1.14		0.39	0.13	0.19	0.16
TAMARO	Wang-Engel response to soil temperature	^{plot}	29776.90	29827.08	0.40	0.00					19.54	25.00	1.36		0.39	0.11	0.16	0.14
TAMARO	Wang-Engel response to soil temperature	^{ref}	30013.90	30064.12	0.41	0.00					20.91	25.00	5.36		0.39	0.12	0.17	0.15
TORONTO	Asym response to air temperature	^{plot}	24084.30	24132.42	0.43	5.36			1.01	1.00					0.41	0.17	0.12	0.12
TORONTO	Asym response to air temperature	^{ref}	24092.20	24140.29	0.43	5.02			1.03	1.00					0.41	0.17	0.12	0.13
TORONTO	Asym response to soil temperature	^{plot}	23858.60	23906.74	0.43	4.53			1.04	1.00					0.41	0.16	0.11	0.11
TORONTO	Asym response to soil temperature	^{ref}	23948.90	23997.04	0.43	6.38			1.77	1.00					0.41	0.16	0.11	0.12
TORONTO	Bi-linear response to air temperature	^{plot}	23639.10	23695.23	0.42	10.97	0.10	0.26							0.41	0.15	0.10	0.10
TORONTO	Bi-linear response to air temperature	^{ref}	23655.50	23711.59	0.42	10.59	0.10	0.27							0.41	0.15	0.10	0.10
TORONTO	Bi-linear response to soil temperature	^{plot}	23335.00	23391.15	0.42	9.98	0.11	0.26							0.41	0.13	0.08	0.09
TORONTO	Bi-linear response to soil temperature	^{ref}	23654.20	23710.35	0.42	7.65	0.17	0.13							0.41	0.15	0.10	0.11
TORONTO	Gaussian process	^{plot}	25115.60	25155.68	0.47									0.20	0.42	0.22	0.17	0.18
TORONTO	Gaussian process	^{ref}	25119.00	25159.07	0.47									0.20	0.42	0.22	0.17	0.18
TORONTO	Linear response to air temperature	^{plot}	23916.10	23964.23	0.43	4.02	0.06								0.41	0.16	0.11	0.12
TORONTO	Linear response to air temperature	^{ref}	23920.20	23968.32	0.43	3.99	0.06								0.41	0.16	0.11	0.12
TORONTO	Linear response to soil temperature	^{plot}	23629.20	23677.26	0.42	4.28	0.07								0.41	0.15	0.10	0.10
TORONTO	Linear response to soil temperature	^{ref}	23802.80	23850.87	0.43	6.01	0.13								0.41	0.16	0.11	0.11

TORONTO	Thermal time to air temperature	^{plot}	24084.70	24124.80	0.43	0.00	0.03								0.41	0.17	0.12	0.13
TORONTO	Thermal time to air temperature	^{ref}	24069.20	24109.26	0.43	0.00	0.04								0.41	0.17	0.12	0.13
TORONTO	Thermal time to soil temperature	^{plot}	23897.60	23937.70	0.43	0.00	0.04								0.41	0.16	0.11	0.12
TORONTO	Thermal time to soil temperature	^{ref}	24569.10	24609.16	0.45	0.00	0.03								0.42	0.20	0.14	0.15
TORONTO	Wang-Engel response to air temperature	^{plot}	23822.90	23870.97	0.43	0.00					19.40	25.00	0.93		0.41	0.16	0.10	0.11
TORONTO	Wang-Engel response to air temperature	^{ref}	23831.90	23880.00	0.43	0.00					19.17	25.00	0.94		0.41	0.16	0.11	0.11
TORONTO	Wang-Engel response to soil temperature	^{plot}	23504.60	23552.66	0.42	0.00					19.10	25.00	1.02		0.41	0.14	0.09	0.10
TORONTO	Wang-Engel response to soil temperature	^{ref}	23617.40	23665.52	0.42	0.00					20.65	25.00	3.52		0.41	0.15	0.10	0.10
WINNETOU	Asym response to air temperature	^{plot}	42769.90	42820.42	0.47	2.70			0.73	1.00					0.46	0.06	0.13	0.14
WINNETOU	Asym response to air temperature	^{ref}	42766.50	42817.01	0.47	2.45			0.76	1.00					0.46	0.05	0.12	0.13
WINNETOU	Asym response to soil temperature	^{plot}	42476.80	42527.30	0.47	3.45			1.02	1.00					0.46	0.05	0.12	0.13
WINNETOU	Asym response to soil temperature	^{ref}	42952.60	43003.13	0.47	4.98			1.51	1.00					0.46	0.06	0.13	0.14
WINNETOU	Bi-linear response to air temperature	^{plot}	41890.20	41949.08	0.46	10.74	0.10	0.31							0.45	0.03	0.09	0.11
WINNETOU	Bi-linear response to air temperature	^{ref}	41908.40	41967.34	0.46	11.63	0.11	0.38							0.45	0.03	0.09	0.11
WINNETOU	Bi-linear response to soil temperature	^{plot}	41423.30	41482.22	0.45	9.66	0.11	0.31							0.45	0.01	0.08	0.09
WINNETOU	Bi-linear response to soil temperature	^{ref}	42399.40	42458.31	0.47	7.33	0.17	0.17							0.46	0.04	0.11	0.13
WINNETOU	Gaussian process	^{plot}	44666.20	44708.23	0.51									0.24	0.47	0.12	0.20	0.20
WINNETOU	Gaussian process	^{ref}	44670.20	44712.24	0.51									0.24	0.47	0.12	0.20	0.20
WINNETOU	Linear response to air temperature	^{plot}	42373.50	42424.03	0.47	2.57	0.05								0.46	0.04	0.11	0.12
WINNETOU	Linear response to air temperature	^{ref}	42374.90	42425.43	0.46	2.08	0.05								0.46	0.04	0.11	0.12
WINNETOU	Linear response to soil temperature	^{plot}	41977.70	42028.18	0.46	2.72	0.06								0.45	0.03	0.10	0.11
WINNETOU	Linear response to soil temperature	^{ref}	42648.40	42698.86	0.47	4.83	0.11								0.46	0.05	0.12	0.13
WINNETOU	Thermal time to air temperature	^{plot}	42554.70	42596.76	0.47	0.00	0.04								0.46	0.05	0.12	0.13
WINNETOU	Thermal time to air temperature	^{ref}	42501.60	42543.70	0.47	0.00	0.04								0.46	0.05	0.12	0.13
WINNETOU	Thermal time to soil temperature	^{plot}	42305.30	42347.34	0.46	0.00	0.04								0.46	0.04	0.11	0.12
WINNETOU	Thermal time to soil temperature	^{ref}	43634.50	43676.58	0.49	0.00	0.04								0.47	0.09	0.16	0.17
WINNETOU	Wang-Engel response to air temperature	^{plot}	42353.70	42404.23	0.46	0.00					18.95	25.00	0.93		0.46	0.04	0.11	0.12
WINNETOU	Wang-Engel response to air temperature	^{ref}	42368.00	42418.48	0.46	0.00					18.67	25.00	0.93		0.46	0.04	0.11	0.12
WINNETOU	Wang-Engel response to soil temperature	^{plot}	41802.00	41852.51	0.46	0.00					18.72	25.00	1.07		0.45	0.02	0.09	0.10
WINNETOU	Wang-Engel response to soil temperature	^{ref}	42332.60	42383.11	0.46	0.00					20.00	25.00	2.69		0.46	0.04	0.11	0.12

Table B.3: Fitted non-linear models to leaf growth tracker (MARTRACK) soybean data. Genotype=ALL denotes the species-specific models.

genotype_name	model	covariate_	AIC	BIC	RMSE	Tmin	a	rmin	lrc	Asym	Topt	Tmax	rmax	mu	sigma	rho_1	rho_2	rho_3
ALL	Asym response to air temperature	T[air]^{ref}	2422.50	2463.24	0.70	7.97			1.04	1.74					0.29	1.00	-0.11	0.00
ALL	Asym response to soil temperature	T[soil]^{ref}	2450.90	2491.55	0.73	15.20			1.44	1.88					0.29	1.01	-0.11	0.00
ALL	Bi-linear response to air temperature	T[air]^{ref}	2441.60	2482.29	0.70	6.79	0.06	0.29							0.29	1.00	-0.11	0.00
ALL	Bi-linear response to soil temperature	T[soil]^{ref}	2467.00	2514.42	0.73	9.39	0.14	-0.62							0.29	1.01	-0.11	0.00
ALL	Gaussian process		2575.90	2602.99	0.85									0.87	0.30	1.02	-0.10	0.00
ALL	Linear response to air temperature	T[air]^{ref}	13203.60	13223.97	0.69	6.84	0.10								0.67	0.00	0.00	0.00
ALL	Linear response to soil temperature	T[soil]^{ref}	13952.40	13972.73	0.72	14.76	0.17								0.71	0.00	0.00	0.00
ALL	Thermal time to air temperature	T[air]^{ref}	2446.20	2473.34	0.70	5.00	0.07								0.29	1.00	-0.11	0.00
ALL	Thermal time to soil temperature	T[soil]^{ref}	2502.80	2529.97	0.77	5.00	0.06								0.29	1.01	-0.10	0.00
ALL	Wang-Engel response to air temperature	T[air]^{ref}	2398.30	2445.77	0.68	2.65					23.72	32.83	1.40		0.29	1.00	-0.11	0.00
ALL	Wang-Engel response to soil temperature	T[soil]^{ref}	2448.50	2495.99	0.72	15.41					24.18	30.91	1.33		0.29	1.01	-0.11	0.00
Castetis	Asym response to air temperature	T[air]^{ref}	457.40	494.19	0.74	7.41			1.30	1.67					0.28	1.09	-0.20	0.01
Castetis	Asym response to soil temperature	T[soil]^{ref}	481.30	512.84	0.81	14.65			1.92	1.48					0.29	1.09	-0.17	0.00
Castetis	Bi-linear response to air temperature	T[air]^{ref}	448.90	480.43	0.74	22.28	-0.05	1.51							0.28	1.08	-0.18	0.00
Castetis	Bi-linear response to soil temperature	T[soil]^{ref}	486.80	523.60	0.81	11.09	0.09	0.14							0.29	1.09	-0.19	0.01
Castetis	Gaussian process		491.60	512.60	0.91									0.99	0.29	1.09	-0.16	0.00
Castetis	Linear response to air temperature	T[air]^{ref}	2967.50	2983.30	0.72	5.80	0.10								0.70	0.00	0.00	0.00
Castetis	Linear response to soil temperature	T[soil]^{ref}	483.20	509.41	0.81	9.67	0.09								0.29	1.08	-0.17	0.00
Castetis	Thermal time to air temperature	T[air]^{ref}	465.60	486.63	0.74	5.00	0.08								0.29	1.09	-0.19	0.00
Castetis	Thermal time to soil temperature	T[soil]^{ref}	482.20	503.18	0.83	5.00	0.07								0.29	1.09	-0.17	0.00
Castetis	Wang-Engel response to air temperature	T[air]^{ref}	449.90	486.66	0.71	-0.54					23.03	31.29	1.53		0.28	1.08	-0.19	0.00
Castetis	Wang-Engel response to soil temperature	T[soil]^{ref}	477.20	514.00	0.80	-29.92					23.36	26.51	1.39		0.29	1.09	-0.18	0.00
Gallec	Asym response to air temperature	T[air]^{ref}	280.20	315.57	0.65	8.32			0.90	1.87					0.25	1.06	-0.17	0.00
Gallec	Asym response to soil temperature	T[soil]^{ref}	290.40	325.78	0.69	15.42			1.85	1.52					0.26	1.07	-0.16	0.00
Gallec	Bi-linear response to air temperature	T[air]^{ref}	288.20	323.56	0.66	11.25	0.07	0.48							0.25	1.06	-0.16	0.00
Gallec	Bi-linear response to soil temperature	T[soil]^{ref}	303.70	339.07	0.68	11.65	0.14	-0.38							0.26	1.07	-0.16	0.00
Gallec	Gaussian process		356.70	380.31	0.80									0.85	0.26	1.09	-0.15	0.00
Gallec	Linear response to air temperature	T[air]^{ref}	287.80	317.21	0.66	3.39	0.07								0.25	1.06	-0.16	0.00
Gallec	Linear response to soil temperature	T[soil]^{ref}	303.60	338.95	0.68	14.31	0.14								0.26	1.07	-0.16	-0.01
Gallec	Thermal time to air temperature	T[air]^{ref}	287.20	310.79	0.65	5.00	0.07								0.26	1.06	-0.16	0.00
Gallec	Thermal time to soil temperature	T[soil]^{ref}	322.20	345.80	0.74	5.00	0.06								0.26	1.08	-0.16	0.00
Gallec	Wang-Engel response to air temperature	T[air]^{ref}	263.40	304.65	0.63	6.93					23.64	32.75	1.42		0.25	1.06	-0.17	0.00
Gallec	Wang-Engel response to soil temperature	T[soil]^{ref}	291.10	332.35	0.69	15.86					25.10	36.37	1.27		0.25	1.07	-0.16	0.00
Opaline	Asym response to air temperature	T[air]^{ref}	1498.40	1527.37	0.71	7.97			1.01	1.71					0.33	0.88	0.00	0.00
Opaline	Asym response to soil temperature	T[soil]^{ref}	1492.30	1521.28	0.72	15.73			1.04	2.60					0.33	0.88	0.00	0.00
Opaline	Bi-linear response to air temperature	T[air]^{ref}	1502.60	1531.57	0.72	10.09	0.06	0.45							0.33	0.88	0.00	0.00
Opaline	Bi-linear response to soil temperature	T[soil]^{ref}	1494.70	1523.65	0.72	13.27	0.16	-0.23							0.33	0.88	0.00	0.00
Opaline	Gaussian process		1544.00	1561.34	0.84									0.83	0.33	0.91	0.00	0.00
Opaline	Linear response to air temperature	T[air]^{ref}	1501.20	1524.34	0.72	2.29	0.06								0.33	0.88	0.00	0.00
Opaline	Linear response to soil temperature	T[soil]^{ref}	1492.70	1515.85	0.72	14.76	0.16								0.33	0.88	0.00	0.00
Opaline	Thermal time to air temperature	T[air]^{ref}	1501.70	1519.06	0.71	5.00	0.07								0.33	0.88	0.00	0.00
Opaline	Thermal time to soil temperature	T[soil]^{ref}	1514.30	1531.71	0.77	5.00	0.06								0.33	0.90	0.00	0.00
Opaline	Wang-Engel response to air temperature	T[air]^{ref}	1497.30	1532.04	0.71	2.80					24.86	35.86	1.34		0.33	0.87	0.00	0.00
Opaline	Wang-Engel response to soil temperature	T[soil]^{ref}	1493.50	1528.26	0.72	13.22					24.43	28.95	1.42		0.33	0.88	0.00	0.00

Table B.4: Fitted non-linear models to plant height data (SfM, TLS) wheat and soybean data Genotype=ALL denotes the species-specific models.

crop	genotype.name	model	covariate_	model_	skipped_year	AIC	BIC	RMSE	Tmin	a	rmin	lrc	Asym	Topt	rmax	mu	sigma_error	rho_error
Soybean	ALL	asym	T[air]^{plot}	Asymptotic	2017.00	6665.57	6689.43		6.37			0.00	2.84				13.21	0.51
Soybean	ALL	asym	T[air]^{plot}	Asymptotic	2018.00	6294.67	6318.30		7.99			0.86	1.69				12.79	0.49
Soybean	ALL	asym	T[air]^{plot}	Asymptotic	2019.00	7357.61	7382.12	12.98	9.67			2.67	0.96				9.75	0.66
Soybean	ALL	asym	T[air]^{plot}	Asymptotic	2020.00	6287.73	6311.35		0.00			0.12	2.02				12.73	0.47
Soybean	ALL	asym	T[air]^{ref}	Asymptotic	2017.00	6665.57	6689.43		6.37			0.00	2.84				13.21	0.51
Soybean	ALL	asym	T[air]^{ref}	Asymptotic	2018.00	6294.67	6318.30		7.99			0.86	1.69				12.79	0.49
Soybean	ALL	asym	T[air]^{ref}	Asymptotic	2019.00	7357.61	7382.12	12.98	9.67			2.67	0.96				9.75	0.66
Soybean	ALL	asym	T[air]^{ref}	Asymptotic	2020.00	6287.73	6311.35		0.00			0.12	2.02				12.73	0.47
Soybean	ALL	bilinear	T[air]^{plot}	Bi-linear	2017.00	6668.06	6691.92		11.46	0.06	0.49						13.23	0.53
Soybean	ALL	bilinear	T[air]^{plot}	Bi-linear	2018.00	6314.86	6338.48		29.91	-0.02	1.46						12.95	0.57
Soybean	ALL	bilinear	T[air]^{plot}	Bi-linear	2019.00	7400.20	7424.72	13.70	17.87	-0.00	0.98						9.96	0.69
Soybean	ALL	bilinear	T[air]^{plot}	Bi-linear	2020.00	6296.65	6320.27		29.87	-0.24	1.61						12.80	0.45
Soybean	ALL	bilinear	T[air]^{ref}	Bi-linear	2017.00	6668.06	6691.92		11.46	0.06	0.49						13.23	0.53
Soybean	ALL	bilinear	T[air]^{ref}	Bi-linear	2018.00	6314.86	6338.48		29.91	-0.02	1.46						12.95	0.57
Soybean	ALL	bilinear	T[air]^{ref}	Bi-linear	2019.00	7400.20	7424.72	13.70	17.87	-0.00	0.98						9.96	0.69
Soybean	ALL	bilinear	T[air]^{ref}	Bi-linear	2020.00	6296.65	6320.27		29.87	-0.24	1.61						12.80	0.45
Soybean	ALL	gauss	^{plot}	Gaussian	2017.00	8836.45	8850.77	38.56								68.34	38.26	0.13
Soybean	ALL	gauss	^{plot}	Gaussian	2018.00	8330.93	8345.11	36.18								73.48	36.02	0.10
Soybean	ALL	gauss	^{plot}	Gaussian	2019.00	9002.85	9017.56	22.34								59.52	22.35	0.02
Soybean	ALL	gauss	^{plot}	Gaussian	2020.00	8376.36	8390.53	37.37								71.89	37.02	0.14
Soybean	ALL	gauss	^{ref}	Gaussian	2017.00	8836.45	8850.77	38.56								68.34	38.26	0.13
Soybean	ALL	gauss	^{ref}	Gaussian	2018.00	8330.93	8345.11	36.18								73.48	36.02	0.10
Soybean	ALL	gauss	^{ref}	Gaussian	2019.00	9002.85	9017.56	22.34								59.52	22.35	0.02
Soybean	ALL	gauss	^{ref}	Gaussian	2020.00	8376.36	8390.53	37.37								71.89	37.02	0.14
Soybean	ALL	linear	T[air]^{plot}	Linear	2017.00	6964.41	6983.49		-45.89	0.01							15.96	0.69
Soybean	ALL	linear	T[air]^{plot}	Linear	2018.00	6572.55	6591.45		-46.28	0.01							15.39	0.69
Soybean	ALL	linear	T[air]^{plot}	Linear	2019.00	7465.38	7485.00	14.06	-17.95	0.02							10.30	0.68
Soybean	ALL	linear	T[air]^{plot}	Linear	2020.00	6334.16	6353.06		-47.80	0.02							13.19	0.49
Soybean	ALL	linear	T[air]^{ref}	Linear	2017.00	6964.41	6983.49		-45.89	0.01							15.96	0.69
Soybean	ALL	linear	T[air]^{ref}	Linear	2018.00	6572.55	6591.45		-46.28	0.01							15.39	0.69
Soybean	ALL	linear	T[air]^{ref}	Linear	2019.00	7465.38	7485.00	14.06	-17.95	0.02							10.30	0.68
Soybean	ALL	linear	T[air]^{ref}	Linear	2020.00	6334.16	6353.06		-47.80	0.02							13.19	0.49
Soybean	ALL	thermal	T[air]^{plot}	Thermal time	2017.00	6680.51	6694.83											
Soybean	ALL	thermal	T[air]^{plot}	Thermal time	2018.00	6312.92	6327.10											
Soybean	ALL	thermal	T[air]^{plot}	Thermal time	2019.00	7557.34	7572.04	12.99										
Soybean	ALL	thermal	T[air]^{plot}	Thermal time	2020.00	6330.86	6345.04											
Soybean	ALL	thermal	T[air]^{ref}	Thermal time	2017.00	6680.51	6694.83											
Soybean	ALL	thermal	T[air]^{ref}	Thermal time	2018.00	6312.92	6327.10											
Soybean	ALL	thermal	T[air]^{ref}	Thermal time	2019.00	7557.34	7572.04	12.99										
Soybean	ALL	thermal	T[air]^{ref}	Thermal time	2020.00	6330.86	6345.04											
Soybean	ALL	wang	T[air]^{plot}	Wang-Engel	2017.00	6729.78	6748.87							26.39	1.49		13.74	0.45
Soybean	ALL	wang	T[air]^{plot}	Wang-Engel	2018.00	6319.43	6338.33							26.24	1.44		13.01	0.47
Soybean	ALL	wang	T[air]^{plot}	Wang-Engel	2019.00	7394.56	7414.17	13.31						22.54	1.06		9.94	0.67
Soybean	ALL	wang	T[air]^{plot}	Wang-Engel	2020.00	6369.39	6388.29							25.08	1.40		13.42	0.40
Soybean	ALL	wang	T[air]^{ref}	Wang-Engel	2017.00	6729.78	6748.87							26.39	1.49		13.74	0.45
Soybean	ALL	wang	T[air]^{ref}	Wang-Engel	2018.00	6319.43	6338.33							26.24	1.44		13.01	0.47
Soybean	ALL	wang	T[air]^{ref}	Wang-Engel	2019.00	7394.56	7414.17	13.31						22.54	1.06		9.94	0.67

Soybean	ALL	wang	T[air]^{ref}	Wang-Engel	2020.00	6369.39	6388.29							25.08	1.40		13.42	0.40
Soybean	Aveline	asym	T[air]^{plot}	Asymptotic	2017.00	523.26	534.57		7.85			0.68	1.94				11.82	0.47
Soybean	Aveline	asym	T[air]^{plot}	Asymptotic	2018.00	513.63	524.87		9.24			1.55	1.32				11.64	0.59
Soybean	Aveline	asym	T[air]^{plot}	Asymptotic	2019.00	595.86	607.89	12.10	9.72			2.86	0.94				9.00	0.67
Soybean	Aveline	asym	T[air]^{plot}	Asymptotic	2020.00	503.28	514.38		4.01			3.90	1.09				12.13	0.53
Soybean	Aveline	asym	T[air]^{ref}	Asymptotic	2017.00	523.26	534.57		7.85			0.68	1.94				11.82	0.47
Soybean	Aveline	asym	T[air]^{ref}	Asymptotic	2018.00	513.63	524.87		9.24			1.55	1.32				11.64	0.59
Soybean	Aveline	asym	T[air]^{ref}	Asymptotic	2019.00	595.86	607.89	12.10	9.72			2.86	0.94				9.00	0.67
Soybean	Aveline	asym	T[air]^{ref}	Asymptotic	2020.00	503.28	514.38		4.01			3.90	1.09				12.13	0.53
Soybean	Aveline	bilinear	T[air]^{plot}	Bi-linear	2017.00	524.51	535.82		8.20	0.06	0.30						11.95	0.51
Soybean	Aveline	bilinear	T[air]^{plot}	Bi-linear	2018.00	518.41	529.65		15.21	0.04	0.78						12.08	0.64
Soybean	Aveline	bilinear	T[air]^{plot}	Bi-linear	2019.00	599.13	611.17	13.13	17.93	-0.02	1.01						9.19	0.72
Soybean	Aveline	bilinear	T[air]^{plot}	Bi-linear	2020.00	492.25	503.35		5.00	0.03	0.69						11.00	0.55
Soybean	Aveline	bilinear	T[air]^{ref}	Bi-linear	2017.00	524.51	535.82		8.20	0.06	0.30						11.95	0.51
Soybean	Aveline	bilinear	T[air]^{ref}	Bi-linear	2018.00	518.41	529.65		15.21	0.04	0.78						12.08	0.64
Soybean	Aveline	bilinear	T[air]^{ref}	Bi-linear	2019.00	599.13	611.17	13.13	17.93	-0.02	1.01						9.19	0.72
Soybean	Aveline	bilinear	T[air]^{ref}	Bi-linear	2020.00	492.25	503.35		5.00	0.03	0.69						11.00	0.55
Soybean	Aveline	gauss	^{plot}	Gaussian	2017.00	715.31	722.10	38.54								69.43	38.38	0.11
Soybean	Aveline	gauss	^{plot}	Gaussian	2018.00	697.48	704.22	36.47								73.46	36.30	0.11
Soybean	Aveline	gauss	^{plot}	Gaussian	2019.00	735.30	742.52	21.80								59.84	21.82	-0.01
Soybean	Aveline	gauss	^{plot}	Gaussian	2020.00	680.34	687.00	37.30								72.86	37.10	0.12
Soybean	Aveline	gauss	^{ref}	Gaussian	2017.00	715.31	722.10	38.54								69.43	38.38	0.11
Soybean	Aveline	gauss	^{ref}	Gaussian	2018.00	697.48	704.22	36.47								73.46	36.30	0.11
Soybean	Aveline	gauss	^{ref}	Gaussian	2019.00	735.30	742.52	21.80								59.84	21.82	-0.01
Soybean	Aveline	gauss	^{ref}	Gaussian	2020.00	680.34	687.00	37.30								72.86	37.10	0.12
Soybean	Aveline	linear	T[air]^{plot}	Linear	2017.00	550.09	559.14		-46.66	0.01							14.83	0.72
Soybean	Aveline	linear	T[air]^{plot}	Linear	2018.00	536.41	545.40		-47.09	0.01							14.21	0.74
Soybean	Aveline	linear	T[air]^{plot}	Linear	2019.00	606.75	616.38	14.24	-50.16	0.01							9.76	0.73
Soybean	Aveline	linear	T[air]^{plot}	Linear	2020.00	492.01	500.89		-49.31	0.02							11.29	0.55
Soybean	Aveline	linear	T[air]^{ref}	Linear	2017.00	550.09	559.14		-46.66	0.01							14.83	0.72
Soybean	Aveline	linear	T[air]^{ref}	Linear	2018.00	536.41	545.40		-47.09	0.01							14.21	0.74
Soybean	Aveline	linear	T[air]^{ref}	Linear	2019.00	606.75	616.38	14.24	-50.16	0.01							9.76	0.73
Soybean	Aveline	linear	T[air]^{ref}	Linear	2020.00	492.01	500.89		-49.31	0.02							11.29	0.55
Soybean	Aveline	thermal	T[air]^{plot}	Thermal time	2017.00	521.85	528.64											
Soybean	Aveline	thermal	T[air]^{plot}	Thermal time	2018.00	514.87	521.62											
Soybean	Aveline	thermal	T[air]^{plot}	Thermal time	2019.00	617.03	624.25	12.43										
Soybean	Aveline	thermal	T[air]^{plot}	Thermal time	2020.00	500.12	506.78											
Soybean	Aveline	thermal	T[air]^{ref}	Thermal time	2017.00	521.85	528.64											
Soybean	Aveline	thermal	T[air]^{ref}	Thermal time	2018.00	514.87	521.62											
Soybean	Aveline	thermal	T[air]^{ref}	Thermal time	2019.00	617.03	624.25	12.43										
Soybean	Aveline	thermal	T[air]^{ref}	Thermal time	2020.00	500.12	506.78											
Soybean	Aveline	wang	T[air]^{plot}	Wang-Engel	2017.00	526.87	535.92							26.32	1.49		12.33	0.39
Soybean	Aveline	wang	T[air]^{plot}	Wang-Engel	2018.00	515.62	524.61							25.69	1.38		12.01	0.57
Soybean	Aveline	wang	T[air]^{plot}	Wang-Engel	2019.00	596.62	606.24	12.68						21.78	1.02		9.16	0.70
Soybean	Aveline	wang	T[air]^{plot}	Wang-Engel	2020.00	502.61	511.49							24.29	1.35		12.26	0.44
Soybean	Aveline	wang	T[air]^{ref}	Wang-Engel	2017.00	526.87	535.92							26.32	1.49		12.33	0.39
Soybean	Aveline	wang	T[air]^{ref}	Wang-Engel	2018.00	515.62	524.61							25.69	1.38		12.01	0.57
Soybean	Aveline	wang	T[air]^{ref}	Wang-Engel	2019.00	596.62	606.24	12.68						21.78	1.02		9.16	0.70
Soybean	Aveline	wang	T[air]^{ref}	Wang-Engel	2020.00	502.61	511.49							24.29	1.35		12.26	0.44
Soybean	Coraline	asym	T[air]^{plot}	Asymptotic	2017.00	592.38	603.97		8.96			0.87	1.77				13.81	0.45
Soybean	Coraline	asym	T[air]^{plot}	Asymptotic	2018.00	553.54	564.85		9.65			1.21	1.51				13.17	0.42
Soybean	Coraline	asym	T[air]^{plot}	Asymptotic	2019.00	615.76	627.91	12.10	9.91			2.49	0.98				9.30	0.64

Soybean	Coraline	asym	T[air]^{plot}	Asymptotic	2020.00	551.14	562.38		4.01			3.89	1.04				13.73	0.38
Soybean	Coraline	asym	T[air]^{ref}	Asymptotic	2017.00	592.38	603.97		8.96			0.87	1.77				13.81	0.45
Soybean	Coraline	asym	T[air]^{ref}	Asymptotic	2018.00	553.54	564.85		9.65			1.21	1.51				13.17	0.42
Soybean	Coraline	asym	T[air]^{ref}	Asymptotic	2019.00	615.76	627.91	12.10	9.91			2.49	0.98				9.30	0.64
Soybean	Coraline	asym	T[air]^{ref}	Asymptotic	2020.00	551.14	562.38		4.01			3.89	1.04				13.73	0.38
Soybean	Coraline	bilinear	T[air]^{plot}	Bi-linear	2017.00	595.46	607.05		16.19	0.06	0.71						14.11	0.50
Soybean	Coraline	bilinear	T[air]^{plot}	Bi-linear	2018.00	557.70	569.01		9.33	0.06	0.30						13.57	0.42
Soybean	Coraline	bilinear	T[air]^{plot}	Bi-linear	2019.00	620.97	633.13	12.94	19.89	-0.00	1.00						9.60	0.67
Soybean	Coraline	bilinear	T[air]^{plot}	Bi-linear	2020.00	539.86	551.10		29.81	-0.25	1.57						12.62	0.36
Soybean	Coraline	bilinear	T[air]^{ref}	Bi-linear	2017.00	595.46	607.05		16.19	0.06	0.71						14.11	0.50
Soybean	Coraline	bilinear	T[air]^{ref}	Bi-linear	2018.00	557.70	569.01		9.33	0.06	0.30						13.57	0.42
Soybean	Coraline	bilinear	T[air]^{ref}	Bi-linear	2019.00	620.97	633.13	12.94	19.89	-0.00	1.00						9.60	0.67
Soybean	Coraline	bilinear	T[air]^{ref}	Bi-linear	2020.00	539.86	551.10		29.81	-0.25	1.57						12.62	0.36
Soybean	Coraline	gauss	^{plot}	Gaussian	2017.00	745.37	752.32	35.73								67.25	35.76	0.03
Soybean	Coraline	gauss	^{plot}	Gaussian	2018.00	699.86	706.65	34.34								71.94	34.37	0.09
Soybean	Coraline	gauss	^{plot}	Gaussian	2019.00	754.12	761.41	21.82								58.95	21.93	0.03
Soybean	Coraline	gauss	^{plot}	Gaussian	2020.00	692.59	699.34	34.87								71.36	35.04	0.02
Soybean	Coraline	gauss	^{ref}	Gaussian	2017.00	745.37	752.32	35.73								67.25	35.76	0.03
Soybean	Coraline	gauss	^{ref}	Gaussian	2018.00	699.86	706.65	34.34								71.94	34.37	0.09
Soybean	Coraline	gauss	^{ref}	Gaussian	2019.00	754.12	761.41	21.82								58.95	21.93	0.03
Soybean	Coraline	gauss	^{ref}	Gaussian	2020.00	692.59	699.34	34.87								71.36	35.04	0.02
Soybean	Coraline	linear	T[air]^{plot}	Linear	2017.00	619.20	628.47		-45.16	0.01							17.01	0.62
Soybean	Coraline	linear	T[air]^{plot}	Linear	2018.00	583.54	592.59		-45.30	0.01							16.81	0.62
Soybean	Coraline	linear	T[air]^{plot}	Linear	2019.00	630.42	640.15	14.87	-49.78	0.01							10.31	0.72
Soybean	Coraline	linear	T[air]^{plot}	Linear	2020.00	539.99	548.99		-48.27	0.02							12.86	0.39
Soybean	Coraline	linear	T[air]^{ref}	Linear	2017.00	619.20	628.47		-45.16	0.01							17.01	0.62
Soybean	Coraline	linear	T[air]^{ref}	Linear	2018.00	583.54	592.59		-45.30	0.01							16.81	0.62
Soybean	Coraline	linear	T[air]^{ref}	Linear	2019.00	630.42	640.15	14.87	-49.78	0.01							10.31	0.72
Soybean	Coraline	linear	T[air]^{ref}	Linear	2020.00	539.99	548.99		-48.27	0.02							12.86	0.39
Soybean	Coraline	thermal	T[air]^{plot}	Thermal time	2017.00	593.11	600.06											
Soybean	Coraline	thermal	T[air]^{plot}	Thermal time	2018.00	556.48	563.27											
Soybean	Coraline	thermal	T[air]^{plot}	Thermal time	2019.00	625.29	632.58	12.16										
Soybean	Coraline	thermal	T[air]^{plot}	Thermal time	2020.00	539.61	546.36											
Soybean	Coraline	thermal	T[air]^{ref}	Thermal time	2017.00	593.11	600.06											
Soybean	Coraline	thermal	T[air]^{ref}	Thermal time	2018.00	556.48	563.27											
Soybean	Coraline	thermal	T[air]^{ref}	Thermal time	2019.00	625.29	632.58	12.16										
Soybean	Coraline	thermal	T[air]^{ref}	Thermal time	2020.00	539.61	546.36											
Soybean	Coraline	wang	T[air]^{plot}	Wang-Engel	2017.00	592.56	601.83							26.62	1.47		14.02	0.41
Soybean	Coraline	wang	T[air]^{plot}	Wang-Engel	2018.00	551.76	560.81							26.66	1.45		13.19	0.41
Soybean	Coraline	wang	T[air]^{plot}	Wang-Engel	2019.00	617.52	627.25	12.45						23.42	1.09		9.52	0.65
Soybean	Coraline	wang	T[air]^{plot}	Wang-Engel	2020.00	543.89	552.88							24.93	1.36		13.20	0.31
Soybean	Coraline	wang	T[air]^{ref}	Wang-Engel	2017.00	592.56	601.83							26.62	1.47		14.02	0.41
Soybean	Coraline	wang	T[air]^{ref}	Wang-Engel	2018.00	551.76	560.81							26.66	1.45		13.19	0.41
Soybean	Coraline	wang	T[air]^{ref}	Wang-Engel	2019.00	617.52	627.25	12.45						23.42	1.09		9.52	0.65
Soybean	Coraline	wang	T[air]^{ref}	Wang-Engel	2020.00	543.89	552.88							24.93	1.36		13.20	0.31
Soybean	Falbala	asym	T[air]^{plot}	Asymptotic	2017.00	620.78	632.63		7.63			0.00	3.14				13.46	0.49
Soybean	Falbala	asym	T[air]^{plot}	Asymptotic	2018.00	544.61	555.85		7.58			0.39	2.28				13.07	0.45
Soybean	Falbala	asym	T[air]^{plot}	Asymptotic	2019.00	683.03	695.53	14.29	9.36			2.64	0.99				10.62	0.67
Soybean	Falbala	asym	T[air]^{plot}	Asymptotic	2020.00	597.66	609.31		3.15			0.00	2.48				13.55	0.53
Soybean	Falbala	asym	T[air]^{ref}	Asymptotic	2017.00	620.78	632.63		7.63			0.00	3.14				13.46	0.49
Soybean	Falbala	asym	T[air]^{ref}	Asymptotic	2018.00	544.61	555.85		7.58			0.39	2.28				13.07	0.45
Soybean	Falbala	asym	T[air]^{ref}	Asymptotic	2019.00	683.03	695.53	14.29	9.36			2.64	0.99				10.62	0.67

Soybean	Falbala	asym	T[air]^{ref}	Asymptotic	2020.00	597.66	609.31		3.15			0.00	2.48				13.55	0.53
Soybean	Falbala	bilinear	T[air]^{plot}	Bi-linear	2017.00	621.17	633.02		11.44	0.07	0.42						13.49	0.50
Soybean	Falbala	bilinear	T[air]^{plot}	Bi-linear	2018.00	545.86	557.10		16.80	0.07	0.76						13.20	0.48
Soybean	Falbala	bilinear	T[air]^{plot}	Bi-linear	2019.00	686.62	699.12	14.97	16.04	0.01	0.93						10.83	0.69
Soybean	Falbala	bilinear	T[air]^{plot}	Bi-linear	2020.00	597.07	608.72		12.51	0.05	0.71						13.49	0.54
Soybean	Falbala	bilinear	T[air]^{ref}	Bi-linear	2017.00	621.17	633.02		11.44	0.07	0.42						13.49	0.50
Soybean	Falbala	bilinear	T[air]^{ref}	Bi-linear	2018.00	545.86	557.10		16.80	0.07	0.76						13.20	0.48
Soybean	Falbala	bilinear	T[air]^{ref}	Bi-linear	2019.00	686.62	699.12	14.97	16.04	0.01	0.93						10.83	0.69
Soybean	Falbala	bilinear	T[air]^{ref}	Bi-linear	2020.00	597.07	608.72		12.51	0.05	0.71						13.49	0.54
Soybean	Falbala	gauss	^{plot}	Gaussian	2017.00	804.25	811.36	40.74								66.09	40.37	0.15
Soybean	Falbala	gauss	^{plot}	Gaussian	2018.00	704.44	711.18	38.12								74.87	38.18	0.10
Soybean	Falbala	gauss	^{plot}	Gaussian	2019.00	828.50	836.00	24.60								57.60	24.58	0.11
Soybean	Falbala	gauss	^{plot}	Gaussian	2020.00	769.48	776.48	39.74								69.38	39.29	0.19
Soybean	Falbala	gauss	^{ref}	Gaussian	2017.00	804.25	811.36	40.74								66.09	40.37	0.15
Soybean	Falbala	gauss	^{ref}	Gaussian	2018.00	704.44	711.18	38.12								74.87	38.18	0.10
Soybean	Falbala	gauss	^{ref}	Gaussian	2019.00	828.50	836.00	24.60								57.60	24.58	0.11
Soybean	Falbala	gauss	^{ref}	Gaussian	2020.00	769.48	776.48	39.74								69.38	39.29	0.19
Soybean	Falbala	linear	T[air]^{plot}	Linear	2017.00	655.43	664.90		-44.97	0.02							17.29	0.66
Soybean	Falbala	linear	T[air]^{plot}	Linear	2018.00	573.42	582.41		-45.43	0.02							16.63	0.65
Soybean	Falbala	linear	T[air]^{plot}	Linear	2019.00	690.98	700.98	16.40	-49.14	0.01							11.24	0.73
Soybean	Falbala	linear	T[air]^{plot}	Linear	2020.00	606.76	616.08		-47.04	0.02							14.69	0.57
Soybean	Falbala	linear	T[air]^{ref}	Linear	2017.00	655.43	664.90		-44.97	0.02							17.29	0.66
Soybean	Falbala	linear	T[air]^{ref}	Linear	2018.00	573.42	582.41		-45.43	0.02							16.63	0.65
Soybean	Falbala	linear	T[air]^{ref}	Linear	2019.00	690.98	700.98	16.40	-49.14	0.01							11.24	0.73
Soybean	Falbala	linear	T[air]^{ref}	Linear	2020.00	606.76	616.08		-47.04	0.02							14.69	0.57
Soybean	Falbala	thermal	T[air]^{plot}	Thermal time	2017.00	622.48	629.59											
Soybean	Falbala	thermal	T[air]^{plot}	Thermal time	2018.00	543.74	550.49											
Soybean	Falbala	thermal	T[air]^{plot}	Thermal time	2019.00	692.28	699.78	13.84										
Soybean	Falbala	thermal	T[air]^{plot}	Thermal time	2020.00	593.60	600.59											
Soybean	Falbala	thermal	T[air]^{ref}	Thermal time	2017.00	622.48	629.59											
Soybean	Falbala	thermal	T[air]^{ref}	Thermal time	2018.00	543.74	550.49											
Soybean	Falbala	thermal	T[air]^{ref}	Thermal time	2019.00	692.28	699.78	13.84										
Soybean	Falbala	thermal	T[air]^{ref}	Thermal time	2020.00	593.60	600.59											
Soybean	Falbala	wang	T[air]^{plot}	Wang-Engel	2017.00	624.21	633.69							26.92	1.60		13.94	0.47
Soybean	Falbala	wang	T[air]^{plot}	Wang-Engel	2018.00	544.76	553.76							26.69	1.54		13.29	0.44
Soybean	Falbala	wang	T[air]^{plot}	Wang-Engel	2019.00	686.00	695.99	14.44						22.65	1.10		10.91	0.65
Soybean	Falbala	wang	T[air]^{plot}	Wang-Engel	2020.00	601.92	611.25							26.09	1.49		14.14	0.49
Soybean	Falbala	wang	T[air]^{ref}	Wang-Engel	2017.00	624.21	633.69							26.92	1.60		13.94	0.47
Soybean	Falbala	wang	T[air]^{ref}	Wang-Engel	2018.00	544.76	553.76							26.69	1.54		13.29	0.44
Soybean	Falbala	wang	T[air]^{ref}	Wang-Engel	2019.00	686.00	695.99	14.44						22.65	1.10		10.91	0.65
Soybean	Falbala	wang	T[air]^{ref}	Wang-Engel	2020.00	601.92	611.25							26.09	1.49		14.14	0.49
Soybean	Galice	asym	T[air]^{plot}	Asymptotic	2017.00	542.11	553.28		9.91			1.57	1.37				15.46	0.51
Soybean	Galice	asym	T[air]^{plot}	Asymptotic	2018.00	515.11	526.35		9.91			1.63	1.27				11.78	0.56
Soybean	Galice	asym	T[air]^{plot}	Asymptotic	2019.00	621.30	633.08	15.98	9.96			2.56	1.00				12.81	0.60
Soybean	Galice	asym	T[air]^{plot}	Asymptotic	2020.00	497.59	508.46		0.00			1.00	1.32				14.07	0.51
Soybean	Galice	asym	T[air]^{ref}	Asymptotic	2017.00	542.11	553.28		9.91			1.57	1.37				15.46	0.51
Soybean	Galice	asym	T[air]^{ref}	Asymptotic	2018.00	515.11	526.35		9.91			1.63	1.27				11.78	0.56
Soybean	Galice	asym	T[air]^{ref}	Asymptotic	2019.00	621.30	633.08	15.98	9.96			2.56	1.00				12.81	0.60
Soybean	Galice	asym	T[air]^{ref}	Asymptotic	2020.00	497.59	508.46		0.00			1.00	1.32				14.07	0.51
Soybean	Galice	bilinear	T[air]^{plot}	Bi-linear	2017.00	545.45	556.62		8.00	0.06	0.30						15.87	0.55
Soybean	Galice	bilinear	T[air]^{plot}	Bi-linear	2018.00	521.91	533.16		14.88	0.04	0.70						12.42	0.61
Soybean	Galice	bilinear	T[air]^{plot}	Bi-linear	2019.00	623.79	635.58	17.16	19.95	-0.02	1.05						13.02	0.66

Soybean	Galice	bilinear	T[air]^{plot}	Bi-linear	2020.00	497.30	508.17		12.69	0.02	0.95						14.04	0.52
Soybean	Galice	bilinear	T[air]^{ref}	Bi-linear	2017.00	545.45	556.62		8.00	0.06	0.30						15.87	0.55
Soybean	Galice	bilinear	T[air]^{ref}	Bi-linear	2018.00	521.91	533.16		14.88	0.04	0.70						12.42	0.61
Soybean	Galice	bilinear	T[air]^{ref}	Bi-linear	2019.00	623.79	635.58	17.16	19.95	-0.02	1.05						13.02	0.66
Soybean	Galice	bilinear	T[air]^{ref}	Bi-linear	2020.00	497.30	508.17		12.69	0.02	0.95						14.04	0.52
Soybean	Galice	gauss	^{plot}	Gaussian	2017.00	692.85	699.55	37.74								70.11	37.77	0.03
Soybean	Galice	gauss	^{plot}	Gaussian	2018.00	686.70	693.44	33.40								72.52	33.57	0.01
Soybean	Galice	gauss	^{plot}	Gaussian	2019.00	713.08	720.15	23.97								60.93	23.86	0.04
Soybean	Galice	gauss	^{plot}	Gaussian	2020.00	648.60	655.13	36.43								74.18	36.65	0.02
Soybean	Galice	gauss	^{ref}	Gaussian	2017.00	692.85	699.55	37.74								70.11	37.77	0.03
Soybean	Galice	gauss	^{ref}	Gaussian	2018.00	686.70	693.44	33.40								72.52	33.57	0.01
Soybean	Galice	gauss	^{ref}	Gaussian	2019.00	713.08	720.15	23.97								60.93	23.86	0.04
Soybean	Galice	gauss	^{ref}	Gaussian	2020.00	648.60	655.13	36.43								74.18	36.65	0.02
Soybean	Galice	linear	T[air]^{plot}	Linear	2017.00	559.56	568.49		-44.39	0.02							18.14	0.68
Soybean	Galice	linear	T[air]^{plot}	Linear	2018.00	541.08	550.07		-46.73	0.01							14.73	0.71
Soybean	Galice	linear	T[air]^{plot}	Linear	2019.00	627.78	637.20	18.58	-47.54	0.01							13.56	0.69
Soybean	Galice	linear	T[air]^{plot}	Linear	2020.00	495.63	504.33		-48.00	0.02							14.09	0.53
Soybean	Galice	linear	T[air]^{ref}	Linear	2017.00	559.56	568.49		-44.39	0.02							18.14	0.68
Soybean	Galice	linear	T[air]^{ref}	Linear	2018.00	541.08	550.07		-46.73	0.01							14.73	0.71
Soybean	Galice	linear	T[air]^{ref}	Linear	2019.00	627.78	637.20	18.58	-47.54	0.01							13.56	0.69
Soybean	Galice	linear	T[air]^{ref}	Linear	2020.00	495.63	504.33		-48.00	0.02							14.09	0.53
Soybean	Galice	thermal	T[air]^{plot}	Thermal time	2017.00	542.04	548.74											
Soybean	Galice	thermal	T[air]^{plot}	Thermal time	2018.00	517.94	524.68											
Soybean	Galice	thermal	T[air]^{plot}	Thermal time	2019.00	627.36	634.43	16.38										
Soybean	Galice	thermal	T[air]^{plot}	Thermal time	2020.00	504.78	511.30											
Soybean	Galice	thermal	T[air]^{ref}	Thermal time	2017.00	542.04	548.74											
Soybean	Galice	thermal	T[air]^{ref}	Thermal time	2018.00	517.94	524.68											
Soybean	Galice	thermal	T[air]^{ref}	Thermal time	2019.00	627.36	634.43	16.38										
Soybean	Galice	thermal	T[air]^{ref}	Thermal time	2020.00	504.78	511.30											
Soybean	Galice	wang	T[air]^{plot}	Wang-Engel	2017.00	541.58	550.52							26.02	1.41		15.64	0.50
Soybean	Galice	wang	T[air]^{plot}	Wang-Engel	2018.00	514.16	523.16							25.95	1.34		11.88	0.58
Soybean	Galice	wang	T[air]^{plot}	Wang-Engel	2019.00	620.44	629.87	16.49						23.00	1.09		12.91	0.63
Soybean	Galice	wang	T[air]^{plot}	Wang-Engel	2020.00	498.65	507.35							23.55	1.33		14.44	0.41
Soybean	Galice	wang	T[air]^{ref}	Wang-Engel	2017.00	541.58	550.52							26.02	1.41		15.64	0.50
Soybean	Galice	wang	T[air]^{ref}	Wang-Engel	2018.00	514.16	523.16							25.95	1.34		11.88	0.58
Soybean	Galice	wang	T[air]^{ref}	Wang-Engel	2019.00	620.44	629.87	16.49						23.00	1.09		12.91	0.63
Soybean	Galice	wang	T[air]^{ref}	Wang-Engel	2020.00	498.65	507.35							23.55	1.33		14.44	0.41
Soybean	Gallec	asym	T[air]^{plot}	Asymptotic	2017.00	576.44	588.03		6.60			0.00	2.88				12.36	0.34
Soybean	Gallec	asym	T[air]^{plot}	Asymptotic	2018.00	539.08	550.32		8.05			0.89	1.68				12.55	0.31
Soybean	Gallec	asym	T[air]^{plot}	Asymptotic	2019.00	602.93	615.14	10.68	9.72			2.66	0.98				8.25	0.65
Soybean	Gallec	asym	T[air]^{plot}	Asymptotic	2020.00	533.77	545.01		0.00			0.03	2.14				12.06	0.31
Soybean	Gallec	asym	T[air]^{ref}	Asymptotic	2017.00	576.44	588.03		6.60			0.00	2.88				12.36	0.34
Soybean	Gallec	asym	T[air]^{ref}	Asymptotic	2018.00	539.08	550.32		8.05			0.89	1.68				12.55	0.31
Soybean	Gallec	asym	T[air]^{ref}	Asymptotic	2019.00	602.93	615.14	10.68	9.72			2.66	0.98				8.25	0.65
Soybean	Gallec	asym	T[air]^{ref}	Asymptotic	2020.00	533.77	545.01		0.00			0.03	2.14				12.06	0.31
Soybean	Gallec	bilinear	T[air]^{plot}	Bi-linear	2017.00	578.84	590.43		17.85	0.07	0.83						12.57	0.37
Soybean	Gallec	bilinear	T[air]^{plot}	Bi-linear	2018.00	540.93	552.18		8.27	0.06	0.31						12.78	0.31
Soybean	Gallec	bilinear	T[air]^{plot}	Bi-linear	2019.00	609.46	621.67	11.34	19.63	-0.01	1.04						8.58	0.67
Soybean	Gallec	bilinear	T[air]^{plot}	Bi-linear	2020.00	533.47	544.71		12.52	0.03	0.79						12.03	0.32
Soybean	Gallec	bilinear	T[air]^{ref}	Bi-linear	2017.00	578.84	590.43		17.85	0.07	0.83						12.57	0.37
Soybean	Gallec	bilinear	T[air]^{ref}	Bi-linear	2018.00	540.93	552.18		8.27	0.06	0.31						12.78	0.31
Soybean	Gallec	bilinear	T[air]^{ref}	Bi-linear	2019.00	609.46	621.67	11.34	19.63	-0.01	1.04						8.58	0.67

Soybean	Gallec	bilinear	T[air]^{ref}	Bi-linear	2020.00	533.47	544.71		12.52	0.03	0.79						12.03	0.32
Soybean	Gallec	gauss	^{plot}	Gaussian	2017.00	747.74	754.69	36.27								68.93	36.34	0.05
Soybean	Gallec	gauss	^{plot}	Gaussian	2018.00	691.66	698.41	34.62								73.84	34.80	0.04
Soybean	Gallec	gauss	^{plot}	Gaussian	2019.00	758.03	765.35	21.41								60.74	21.27	-0.07
Soybean	Gallec	gauss	^{plot}	Gaussian	2020.00	694.58	701.32	35.40								72.91	35.54	0.06
Soybean	Gallec	gauss	^{ref}	Gaussian	2017.00	747.74	754.69	36.27								68.93	36.34	0.05
Soybean	Gallec	gauss	^{ref}	Gaussian	2018.00	691.66	698.41	34.62								73.84	34.80	0.04
Soybean	Gallec	gauss	^{ref}	Gaussian	2019.00	758.03	765.35	21.41								60.74	21.27	-0.07
Soybean	Gallec	gauss	^{ref}	Gaussian	2020.00	694.58	701.32	35.40								72.91	35.54	0.06
Soybean	Gallec	linear	T[air]^{plot}	Linear	2017.00	608.49	617.76		-46.00	0.01							15.80	0.55
Soybean	Gallec	linear	T[air]^{plot}	Linear	2018.00	566.60	575.60		-46.01	0.01							15.78	0.55
Soybean	Gallec	linear	T[air]^{plot}	Linear	2019.00	619.48	629.25	13.30	-50.52	0.01							9.24	0.74
Soybean	Gallec	linear	T[air]^{plot}	Linear	2020.00	537.78	546.77		-48.55	0.02							12.65	0.32
Soybean	Gallec	linear	T[air]^{ref}	Linear	2017.00	608.49	617.76		-46.00	0.01							15.80	0.55
Soybean	Gallec	linear	T[air]^{ref}	Linear	2018.00	566.60	575.60		-46.01	0.01							15.78	0.55
Soybean	Gallec	linear	T[air]^{ref}	Linear	2019.00	619.48	629.25	13.30	-50.52	0.01							9.24	0.74
Soybean	Gallec	linear	T[air]^{ref}	Linear	2020.00	537.78	546.77		-48.55	0.02							12.65	0.32
Soybean	Gallec	thermal	T[air]^{plot}	Thermal time	2017.00	576.55	583.51											
Soybean	Gallec	thermal	T[air]^{plot}	Thermal time	2018.00	538.25	545.00											
Soybean	Gallec	thermal	T[air]^{plot}	Thermal time	2019.00	618.48	625.81	10.63										
Soybean	Gallec	thermal	T[air]^{plot}	Thermal time	2020.00	533.64	540.38											
Soybean	Gallec	thermal	T[air]^{ref}	Thermal time	2017.00	576.55	583.51											
Soybean	Gallec	thermal	T[air]^{ref}	Thermal time	2018.00	538.25	545.00											
Soybean	Gallec	thermal	T[air]^{ref}	Thermal time	2019.00	618.48	625.81	10.63										
Soybean	Gallec	thermal	T[air]^{ref}	Thermal time	2020.00	533.64	540.38											
Soybean	Gallec	wang	T[air]^{plot}	Wang-Engel	2017.00	577.36	586.63							26.29	1.49		12.62	0.31
Soybean	Gallec	wang	T[air]^{plot}	Wang-Engel	2018.00	539.89	548.88							26.10	1.44		12.81	0.32
Soybean	Gallec	wang	T[air]^{plot}	Wang-Engel	2019.00	606.10	615.87	11.03						22.95	1.11		8.51	0.65
Soybean	Gallec	wang	T[air]^{plot}	Wang-Engel	2020.00	535.24	544.23							25.13	1.42		12.38	0.20
Soybean	Gallec	wang	T[air]^{ref}	Wang-Engel	2017.00	577.36	586.63							26.29	1.49		12.62	0.31
Soybean	Gallec	wang	T[air]^{ref}	Wang-Engel	2018.00	539.89	548.88							26.10	1.44		12.81	0.32
Soybean	Gallec	wang	T[air]^{ref}	Wang-Engel	2019.00	606.10	615.87	11.03						22.95	1.11		8.51	0.65
Soybean	Gallec	wang	T[air]^{ref}	Wang-Engel	2020.00	535.24	544.23							25.13	1.42		12.38	0.20
Soybean	Idefix	asym	T[air]^{plot}	Asymptotic	2017.00	505.08	516.10		6.12			0.00	2.99				11.58	0.47
Soybean	Idefix	asym	T[air]^{plot}	Asymptotic	2018.00	508.09	519.04		8.72			1.33	1.47				12.60	0.52
Soybean	Idefix	asym	T[air]^{plot}	Asymptotic	2019.00	569.36	581.21	12.06	9.91			3.01	1.02				8.73	0.70
Soybean	Idefix	asym	T[air]^{plot}	Asymptotic	2020.00	518.33	529.35		4.01			3.89	1.10				12.84	0.51
Soybean	Idefix	asym	T[air]^{ref}	Asymptotic	2017.00	505.08	516.10		6.12			0.00	2.99				11.58	0.47
Soybean	Idefix	asym	T[air]^{ref}	Asymptotic	2018.00	508.09	519.04		8.72			1.33	1.47				12.60	0.52
Soybean	Idefix	asym	T[air]^{ref}	Asymptotic	2019.00	569.36	581.21	12.06	9.91			3.01	1.02				8.73	0.70
Soybean	Idefix	asym	T[air]^{ref}	Asymptotic	2020.00	518.33	529.35		4.01			3.89	1.10				12.84	0.51
Soybean	Idefix	bilinear	T[air]^{plot}	Bi-linear	2017.00	505.74	516.77		11.41	0.06	0.53						11.64	0.49
Soybean	Idefix	bilinear	T[air]^{plot}	Bi-linear	2018.00	511.41	522.36		13.69	0.05	0.72						12.95	0.53
Soybean	Idefix	bilinear	T[air]^{plot}	Bi-linear	2019.00	576.84	588.68	12.76	17.87	-0.01	1.10						9.16	0.70
Soybean	Idefix	bilinear	T[air]^{plot}	Bi-linear	2020.00	502.74	513.76		11.90	0.03	0.83						11.36	0.56
Soybean	Idefix	bilinear	T[air]^{ref}	Bi-linear	2017.00	505.74	516.77		11.41	0.06	0.53						11.64	0.49
Soybean	Idefix	bilinear	T[air]^{ref}	Bi-linear	2018.00	511.41	522.36		13.69	0.05	0.72						12.95	0.53
Soybean	Idefix	bilinear	T[air]^{ref}	Bi-linear	2019.00	576.84	588.68	12.76	17.87	-0.01	1.10						9.16	0.70
Soybean	Idefix	bilinear	T[air]^{ref}	Bi-linear	2020.00	502.74	513.76		11.90	0.03	0.83						11.36	0.56
Soybean	Idefix	gauss	^{plot}	Gaussian	2017.00	680.27	686.88	39.99								73.92	40.01	0.02
Soybean	Idefix	gauss	^{plot}	Gaussian	2018.00	663.44	670.01	38.01								76.07	38.03	0.05
Soybean	Idefix	gauss	^{plot}	Gaussian	2019.00	717.19	724.30	23.28								62.85	23.10	-0.13

Soybean	Idefix	gauss	^{plot}	Gaussian	2020.00	671.99	678.60	37.65								74.03	37.58	0.09
Soybean	Idefix	gauss	^{ref}	Gaussian	2017.00	680.27	686.88	39.99								73.92	40.01	0.02
Soybean	Idefix	gauss	^{ref}	Gaussian	2018.00	663.44	670.01	38.01								76.07	38.03	0.05
Soybean	Idefix	gauss	^{ref}	Gaussian	2019.00	717.19	724.30	23.28								62.85	23.10	-0.13
Soybean	Idefix	gauss	^{ref}	Gaussian	2020.00	671.99	678.60	37.65								74.03	37.58	0.09
Soybean	Idefix	linear	T[air]^{plot}	Linear	2017.00	537.77	546.59		-46.31	0.02							15.35	0.65
Soybean	Idefix	linear	T[air]^{plot}	Linear	2018.00	509.51	518.27		0.19	0.05							12.95	0.52
Soybean	Idefix	linear	T[air]^{plot}	Linear	2019.00	586.69	596.17	14.21	-50.06	0.01							9.90	0.72
Soybean	Idefix	linear	T[air]^{plot}	Linear	2020.00	504.83	513.65		-49.05	0.02							11.77	0.55
Soybean	Idefix	linear	T[air]^{ref}	Linear	2017.00	537.77	546.59		-46.31	0.02							15.35	0.65
Soybean	Idefix	linear	T[air]^{ref}	Linear	2018.00	509.51	518.27		0.19	0.05							12.95	0.52
Soybean	Idefix	linear	T[air]^{ref}	Linear	2019.00	586.69	596.17	14.21	-50.06	0.01							9.90	0.72
Soybean	Idefix	linear	T[air]^{ref}	Linear	2020.00	504.83	513.65		-49.05	0.02							11.77	0.55
Soybean	Idefix	thermal	T[air]^{plot}	Thermal time	2017.00	503.53	510.15											
Soybean	Idefix	thermal	T[air]^{plot}	Thermal time	2018.00	507.51	514.08											
Soybean	Idefix	thermal	T[air]^{plot}	Thermal time	2019.00	596.42	603.53	12.88										
Soybean	Idefix	thermal	T[air]^{plot}	Thermal time	2020.00	506.82	513.44											
Soybean	Idefix	thermal	T[air]^{ref}	Thermal time	2017.00	503.53	510.15											
Soybean	Idefix	thermal	T[air]^{ref}	Thermal time	2018.00	507.51	514.08											
Soybean	Idefix	thermal	T[air]^{ref}	Thermal time	2019.00	596.42	603.53	12.88										
Soybean	Idefix	thermal	T[air]^{ref}	Thermal time	2020.00	506.82	513.44											
Soybean	Idefix	wang	T[air]^{plot}	Wang-Engel	2017.00	507.86	516.68							26.25	1.58		12.02	0.40
Soybean	Idefix	wang	T[air]^{plot}	Wang-Engel	2018.00	507.42	516.18							25.76	1.46		12.74	0.50
Soybean	Idefix	wang	T[air]^{plot}	Wang-Engel	2019.00	572.34	581.82	11.83						22.16	1.14		9.01	0.66
Soybean	Idefix	wang	T[air]^{plot}	Wang-Engel	2020.00	512.18	521.00							24.89	1.42		12.43	0.46
Soybean	Idefix	wang	T[air]^{ref}	Wang-Engel	2017.00	507.86	516.68							26.25	1.58		12.02	0.40
Soybean	Idefix	wang	T[air]^{ref}	Wang-Engel	2018.00	507.42	516.18							25.76	1.46		12.74	0.50
Soybean	Idefix	wang	T[air]^{ref}	Wang-Engel	2019.00	572.34	581.82	11.83						22.16	1.14		9.01	0.66
Soybean	Idefix	wang	T[air]^{ref}	Wang-Engel	2020.00	512.18	521.00							24.89	1.42		12.43	0.46
Soybean	Merlin	asym	T[air]^{plot}	Asymptotic	2017.00	605.82	617.54		8.45			0.89	1.68				13.55	0.45
Soybean	Merlin	asym	T[air]^{plot}	Asymptotic	2018.00	551.52	562.76		9.00			1.23	1.39				13.77	0.47
Soybean	Merlin	asym	T[air]^{plot}	Asymptotic	2019.00	640.19	652.46	13.14	9.19			2.64	0.89				9.86	0.67
Soybean	Merlin	asym	T[air]^{plot}	Asymptotic	2020.00	572.83	584.28		0.00			0.35	1.66				13.48	0.46
Soybean	Merlin	asym	T[air]^{ref}	Asymptotic	2017.00	605.82	617.54		8.45			0.89	1.68				13.55	0.45
Soybean	Merlin	asym	T[air]^{ref}	Asymptotic	2018.00	551.52	562.76		9.00			1.23	1.39				13.77	0.47
Soybean	Merlin	asym	T[air]^{ref}	Asymptotic	2019.00	640.19	652.46	13.14	9.19			2.64	0.89				9.86	0.67
Soybean	Merlin	asym	T[air]^{ref}	Asymptotic	2020.00	572.83	584.28		0.00			0.35	1.66				13.48	0.46
Soybean	Merlin	bilinear	T[air]^{plot}	Bi-linear	2017.00	607.34	619.06		8.69	0.06	0.30						13.70	0.47
Soybean	Merlin	bilinear	T[air]^{plot}	Bi-linear	2018.00	554.31	565.56		17.36	0.05	0.77						14.05	0.51
Soybean	Merlin	bilinear	T[air]^{plot}	Bi-linear	2019.00	642.09	654.37	13.67	16.05	-0.00	0.87						9.97	0.69
Soybean	Merlin	bilinear	T[air]^{plot}	Bi-linear	2020.00	572.47	583.92		12.51	0.03	0.78						13.45	0.47
Soybean	Merlin	bilinear	T[air]^{ref}	Bi-linear	2017.00	607.34	619.06		8.69	0.06	0.30						13.70	0.47
Soybean	Merlin	bilinear	T[air]^{ref}	Bi-linear	2018.00	554.31	565.56		17.36	0.05	0.77						14.05	0.51
Soybean	Merlin	bilinear	T[air]^{ref}	Bi-linear	2019.00	642.09	654.37	13.67	16.05	-0.00	0.87						9.97	0.69
Soybean	Merlin	bilinear	T[air]^{ref}	Bi-linear	2020.00	572.47	583.92		12.51	0.03	0.78						13.45	0.47
Soybean	Merlin	gauss	^{plot}	Gaussian	2017.00	765.79	772.83	36.01								65.26	35.86	0.13
Soybean	Merlin	gauss	^{plot}	Gaussian	2018.00	687.31	694.06	33.60								71.15	33.72	0.07
Soybean	Merlin	gauss	^{plot}	Gaussian	2019.00	761.65	769.01	20.69								56.30	20.62	-0.00
Soybean	Merlin	gauss	^{plot}	Gaussian	2020.00	721.05	727.92	34.97								68.43	34.70	0.17
Soybean	Merlin	gauss	^{ref}	Gaussian	2017.00	765.79	772.83	36.01								65.26	35.86	0.13
Soybean	Merlin	gauss	^{ref}	Gaussian	2018.00	687.31	694.06	33.60								71.15	33.72	0.07
Soybean	Merlin	gauss	^{ref}	Gaussian	2019.00	761.65	769.01	20.69								56.30	20.62	-0.00

Soybean	Merlin	gauss	^{ref}	Gaussian	2020.00	721.05	727.92	34.97								68.43	34.70	0.17
Soybean	Merlin	linear	T[air]^{plot}	Linear	2017.00	605.31	614.69		3.56	0.06							13.69	0.47
Soybean	Merlin	linear	T[air]^{plot}	Linear	2018.00	552.01	561.01		1.93	0.05							14.03	0.50
Soybean	Merlin	linear	T[air]^{plot}	Linear	2019.00	644.95	654.77	14.47	-49.81	0.01							10.26	0.71
Soybean	Merlin	linear	T[air]^{plot}	Linear	2020.00	573.17	582.34		-47.56	0.01							13.74	0.47
Soybean	Merlin	linear	T[air]^{ref}	Linear	2017.00	605.31	614.69		3.56	0.06							13.69	0.47
Soybean	Merlin	linear	T[air]^{ref}	Linear	2018.00	552.01	561.01		1.93	0.05							14.03	0.50
Soybean	Merlin	linear	T[air]^{ref}	Linear	2019.00	644.95	654.77	14.47	-49.81	0.01							10.26	0.71
Soybean	Merlin	linear	T[air]^{ref}	Linear	2020.00	573.17	582.34		-47.56	0.01							13.74	0.47
Soybean	Merlin	thermal	T[air]^{plot}	Thermal time	2017.00	604.95	611.98											
Soybean	Merlin	thermal	T[air]^{plot}	Thermal time	2018.00	550.39	557.14											
Soybean	Merlin	thermal	T[air]^{plot}	Thermal time	2019.00	652.88	660.24	12.94										
Soybean	Merlin	thermal	T[air]^{plot}	Thermal time	2020.00	573.59	580.46											
Soybean	Merlin	thermal	T[air]^{ref}	Thermal time	2017.00	604.95	611.98											
Soybean	Merlin	thermal	T[air]^{ref}	Thermal time	2018.00	550.39	557.14											
Soybean	Merlin	thermal	T[air]^{ref}	Thermal time	2019.00	652.88	660.24	12.94										
Soybean	Merlin	thermal	T[air]^{ref}	Thermal time	2020.00	573.59	580.46											
Soybean	Merlin	wang	T[air]^{plot}	Wang-Engel	2017.00	606.72	616.10							26.22	1.39		13.83	0.43
Soybean	Merlin	wang	T[air]^{plot}	Wang-Engel	2018.00	549.16	558.15							26.14	1.35		13.73	0.47
Soybean	Merlin	wang	T[air]^{plot}	Wang-Engel	2019.00	640.31	650.13	13.48						21.88	0.97		9.98	0.68
Soybean	Merlin	wang	T[air]^{plot}	Wang-Engel	2020.00	575.25	584.42							24.68	1.30		13.91	0.40
Soybean	Merlin	wang	T[air]^{ref}	Wang-Engel	2017.00	606.72	616.10							26.22	1.39		13.83	0.43
Soybean	Merlin	wang	T[air]^{ref}	Wang-Engel	2018.00	549.16	558.15							26.14	1.35		13.73	0.47
Soybean	Merlin	wang	T[air]^{ref}	Wang-Engel	2019.00	640.31	650.13	13.48						21.88	0.97		9.98	0.68
Soybean	Merlin	wang	T[air]^{ref}	Wang-Engel	2020.00	575.25	584.42							24.68	1.30		13.91	0.40
Soybean	Obelix	asym	T[air]^{plot}	Asymptotic	2017.00	418.66	429.13		8.40	0.07	0.30						11.43	0.41
Soybean	Obelix	asym	T[air]^{plot}	Asymptotic	2017.00	416.35	426.82		8.49			0.94	1.86				11.18	0.46
Soybean	Obelix	asym	T[air]^{plot}	Asymptotic	2018.00	479.15	490.24		8.43			1.27	1.47				11.32	0.54
Soybean	Obelix	asym	T[air]^{plot}	Asymptotic	2019.00	530.85	542.16	12.06	9.96			2.82	0.98				10.00	0.57
Soybean	Obelix	asym	T[air]^{plot}	Asymptotic	2020.00	417.69	428.08		22.23	-0.01	1.36						12.20	0.59
Soybean	Obelix	asym	T[air]^{plot}	Asymptotic	2020.00	428.73	439.12		4.00			3.93	1.18				13.56	0.63
Soybean	Obelix	asym	T[air]^{ref}	Asymptotic	2017.00	418.66	429.13		8.40	0.07	0.30						11.43	0.41
Soybean	Obelix	asym	T[air]^{ref}	Asymptotic	2017.00	416.35	426.82		8.49			0.94	1.86				11.18	0.46
Soybean	Obelix	asym	T[air]^{ref}	Asymptotic	2018.00	479.15	490.24		8.43			1.27	1.47				11.32	0.54
Soybean	Obelix	asym	T[air]^{ref}	Asymptotic	2019.00	530.85	542.16	12.06	9.96			2.82	0.98				10.00	0.57
Soybean	Obelix	asym	T[air]^{ref}	Asymptotic	2020.00	417.69	428.08		22.23	-0.01	1.36						12.20	0.59
Soybean	Obelix	asym	T[air]^{ref}	Asymptotic	2020.00	428.73	439.12		4.00			3.93	1.18				13.56	0.63
Soybean	Obelix	bilinear	T[air]^{plot}	Bi-linear	2018.00	482.26	493.36		13.87	0.05	0.70						11.61	0.53
Soybean	Obelix	bilinear	T[air]^{plot}	Bi-linear	2019.00	532.97	544.29	12.41	22.23	-0.05	1.17						10.14	0.59
Soybean	Obelix	bilinear	T[air]^{ref}	Bi-linear	2018.00	482.26	493.36		13.87	0.05	0.70						11.61	0.53
Soybean	Obelix	bilinear	T[air]^{ref}	Bi-linear	2019.00	532.97	544.29	12.41	22.23	-0.05	1.17						10.14	0.59
Soybean	Obelix	gauss	^{plot}	Gaussian	2017.00	617.97	624.25	43.22								75.60	43.26	0.09
Soybean	Obelix	gauss	^{plot}	Gaussian	2018.00	685.33	691.98	38.49								75.71	38.50	0.11
Soybean	Obelix	gauss	^{plot}	Gaussian	2019.00	635.00	641.79	21.81								61.60	21.63	-0.12
Soybean	Obelix	gauss	^{plot}	Gaussian	2020.00	601.87	608.10	41.10								76.95	41.18	0.11
Soybean	Obelix	gauss	^{ref}	Gaussian	2017.00	617.97	624.25	43.22								75.60	43.26	0.09
Soybean	Obelix	gauss	^{ref}	Gaussian	2018.00	685.33	691.98	38.49								75.71	38.50	0.11
Soybean	Obelix	gauss	^{ref}	Gaussian	2019.00	635.00	641.79	21.81								61.60	21.63	-0.12
Soybean	Obelix	gauss	^{ref}	Gaussian	2020.00	601.87	608.10	41.10								76.95	41.18	0.11
Soybean	Obelix	linear	T[air]^{plot}	Linear	2017.00	449.30	457.68		-46.31	0.02							15.74	0.72
Soybean	Obelix	linear	T[air]^{plot}	Linear	2018.00	503.93	512.81		-47.07	0.02							14.24	0.71
Soybean	Obelix	linear	T[air]^{plot}	Linear	2019.00	541.79	550.85	13.99	-49.32	0.01							10.97	0.63

Soybean	Obelix	linear	T[air]^{plot}	Linear	2020.00	417.75	426.07		-48.77	0.02							12.47	0.65
Soybean	Obelix	linear	T[air]^{ref}	Linear	2017.00	449.30	457.68		-46.31	0.02							15.74	0.72
Soybean	Obelix	linear	T[air]^{ref}	Linear	2018.00	503.93	512.81		-47.07	0.02							14.24	0.71
Soybean	Obelix	linear	T[air]^{ref}	Linear	2019.00	541.79	550.85	13.99	-49.32	0.01							10.97	0.63
Soybean	Obelix	linear	T[air]^{ref}	Linear	2020.00	417.75	426.07		-48.77	0.02							12.47	0.65
Soybean	Obelix	thermal	T[air]^{plot}	Thermal time	2017.00	417.30	423.59											
Soybean	Obelix	thermal	T[air]^{plot}	Thermal time	2018.00	478.29	484.94											
Soybean	Obelix	thermal	T[air]^{plot}	Thermal time	2019.00	544.76	551.55	12.67										
Soybean	Obelix	thermal	T[air]^{plot}	Thermal time	2020.00	421.97	428.21											
Soybean	Obelix	thermal	T[air]^{ref}	Thermal time	2017.00	417.30	423.59											
Soybean	Obelix	thermal	T[air]^{ref}	Thermal time	2018.00	478.29	484.94											
Soybean	Obelix	thermal	T[air]^{ref}	Thermal time	2019.00	544.76	551.55	12.67										
Soybean	Obelix	thermal	T[air]^{ref}	Thermal time	2020.00	421.97	428.21											
Soybean	Obelix	wang	T[air]^{plot}	Wang-Engel	2017.00	418.46	426.84							26.21	1.60		11.63	0.44
Soybean	Obelix	wang	T[air]^{plot}	Wang-Engel	2018.00	479.76	488.64							25.71	1.44		11.56	0.51
Soybean	Obelix	wang	T[air]^{plot}	Wang-Engel	2019.00	529.04	538.09	12.03						22.64	1.10		10.00	0.57
Soybean	Obelix	wang	T[air]^{plot}	Wang-Engel	2020.00	419.12	427.43							24.63	1.46		12.61	0.52
Soybean	Obelix	wang	T[air]^{ref}	Wang-Engel	2017.00	418.46	426.84							26.21	1.60		11.63	0.44
Soybean	Obelix	wang	T[air]^{ref}	Wang-Engel	2018.00	479.76	488.64							25.71	1.44		11.56	0.51
Soybean	Obelix	wang	T[air]^{ref}	Wang-Engel	2019.00	529.04	538.09	12.03						22.64	1.10		10.00	0.57
Soybean	Obelix	wang	T[air]^{ref}	Wang-Engel	2020.00	419.12	427.43							24.63	1.46		12.61	0.52
Soybean	Opaline	asym	T[air]^{plot}	Asymptotic	2017.00	474.98	486.15		7.67			0.00	3.41				10.29	0.46
Soybean	Opaline	asym	T[air]^{plot}	Asymptotic	2018.00	458.93	469.80		9.38	0.07	0.30						11.60	0.38
Soybean	Opaline	asym	T[air]^{plot}	Asymptotic	2018.00	456.01	466.89		9.19			0.95	1.91				11.31	0.47
Soybean	Opaline	asym	T[air]^{plot}	Asymptotic	2019.00	562.92	574.83	10.21	9.38			2.22	1.10				8.01	0.62
Soybean	Opaline	asym	T[air]^{plot}	Asymptotic	2020.00	490.04	501.14		4.01			3.88	1.20				12.38	0.57
Soybean	Opaline	asym	T[air]^{ref}	Asymptotic	2017.00	474.98	486.15		7.67			0.00	3.41				10.29	0.46
Soybean	Opaline	asym	T[air]^{ref}	Asymptotic	2018.00	458.93	469.80		9.38	0.07	0.30						11.60	0.38
Soybean	Opaline	asym	T[air]^{ref}	Asymptotic	2018.00	456.01	466.89		9.19			0.95	1.91				11.31	0.47
Soybean	Opaline	asym	T[air]^{ref}	Asymptotic	2019.00	562.92	574.83	10.21	9.38			2.22	1.10				8.01	0.62
Soybean	Opaline	asym	T[air]^{ref}	Asymptotic	2020.00	490.04	501.14		4.01			3.88	1.20				12.38	0.57
Soybean	Opaline	bilinear	T[air]^{plot}	Bi-linear	2017.00	475.55	486.72		11.45	0.08	0.44						10.34	0.46
Soybean	Opaline	bilinear	T[air]^{plot}	Bi-linear	2019.00	572.92	584.83	10.83	9.43	0.04	0.59						8.54	0.62
Soybean	Opaline	bilinear	T[air]^{plot}	Bi-linear	2020.00	452.33	463.43		30.00	-0.13	1.76						9.09	0.52
Soybean	Opaline	bilinear	T[air]^{ref}	Bi-linear	2017.00	475.55	486.72		11.45	0.08	0.44						10.34	0.46
Soybean	Opaline	bilinear	T[air]^{ref}	Bi-linear	2019.00	572.92	584.83	10.83	9.43	0.04	0.59						8.54	0.62
Soybean	Opaline	bilinear	T[air]^{ref}	Bi-linear	2020.00	452.33	463.43		30.00	-0.13	1.76						9.09	0.52
Soybean	Opaline	gauss	^{plot}	Gaussian	2017.00	707.11	713.82	42.13								72.93	41.94	0.11
Soybean	Opaline	gauss	^{plot}	Gaussian	2018.00	662.74	669.27	40.94								78.12	40.93	0.11
Soybean	Opaline	gauss	^{plot}	Gaussian	2019.00	722.43	729.57	22.93								62.10	22.54	-0.12
Soybean	Opaline	gauss	^{plot}	Gaussian	2020.00	691.97	698.62	40.71								74.83	40.46	0.15
Soybean	Opaline	gauss	^{ref}	Gaussian	2017.00	707.11	713.82	42.13								72.93	41.94	0.11
Soybean	Opaline	gauss	^{ref}	Gaussian	2018.00	662.74	669.27	40.94								78.12	40.93	0.11
Soybean	Opaline	gauss	^{ref}	Gaussian	2019.00	722.43	729.57	22.93								62.10	22.54	-0.12
Soybean	Opaline	gauss	^{ref}	Gaussian	2020.00	691.97	698.62	40.71								74.83	40.46	0.15
Soybean	Opaline	linear	T[air]^{plot}	Linear	2017.00	524.02	532.95		-46.04	0.02							15.73	0.75
Soybean	Opaline	linear	T[air]^{plot}	Linear	2018.00	496.97	505.67		-45.43	0.02							16.61	0.74
Soybean	Opaline	linear	T[air]^{plot}	Linear	2019.00	584.08	593.61	13.30	-50.47	0.01							9.31	0.72
Soybean	Opaline	linear	T[air]^{plot}	Linear	2020.00	447.93	456.80		-6.57	0.04							8.91	0.59
Soybean	Opaline	linear	T[air]^{ref}	Linear	2017.00	524.02	532.95		-46.04	0.02							15.73	0.75
Soybean	Opaline	linear	T[air]^{ref}	Linear	2018.00	496.97	505.67		-45.43	0.02							16.61	0.74
Soybean	Opaline	linear	T[air]^{ref}	Linear	2019.00	584.08	593.61	13.30	-50.47	0.01							9.31	0.72

Soybean	Opaline	linear	T[air]^{ref}	Linear	2020.00	447.93	456.80		-6.57	0.04							8.91	0.59
Soybean	Opaline	thermal	T[air]^{plot}	Thermal time	2017.00	479.42	486.12											
Soybean	Opaline	thermal	T[air]^{plot}	Thermal time	2018.00	459.94	466.47											
Soybean	Opaline	thermal	T[air]^{plot}	Thermal time	2019.00	572.25	579.40	10.14										
Soybean	Opaline	thermal	T[air]^{plot}	Thermal time	2020.00	450.53	457.19											
Soybean	Opaline	thermal	T[air]^{ref}	Thermal time	2017.00	479.42	486.12											
Soybean	Opaline	thermal	T[air]^{ref}	Thermal time	2018.00	459.94	466.47											
Soybean	Opaline	thermal	T[air]^{ref}	Thermal time	2019.00	572.25	579.40	10.14										
Soybean	Opaline	thermal	T[air]^{ref}	Thermal time	2020.00	450.53	457.19											
Soybean	Opaline	wang	T[air]^{plot}	Wang-Engel	2017.00	483.58	492.51							26.96	1.74		11.20	0.39
Soybean	Opaline	wang	T[air]^{plot}	Wang-Engel	2018.00	457.50	466.20							26.82	1.67		11.66	0.42
Soybean	Opaline	wang	T[air]^{plot}	Wang-Engel	2019.00	569.39	578.92	10.41						23.75	1.20		8.45	0.59
Soybean	Opaline	wang	T[air]^{plot}	Wang-Engel	2020.00	467.68	476.56							25.80	1.61		10.47	0.35
Soybean	Opaline	wang	T[air]^{ref}	Wang-Engel	2017.00	483.58	492.51							26.96	1.74		11.20	0.39
Soybean	Opaline	wang	T[air]^{ref}	Wang-Engel	2018.00	457.50	466.20							26.82	1.67		11.66	0.42
Soybean	Opaline	wang	T[air]^{ref}	Wang-Engel	2019.00	569.39	578.92	10.41						23.75	1.20		8.45	0.59
Soybean	Opaline	wang	T[air]^{ref}	Wang-Engel	2020.00	467.68	476.56							25.80	1.61		10.47	0.35
Soybean	Protéix	asym	T[air]^{plot}	Asymptotic	2017.00	563.38	575.10		9.62			1.41	1.46				12.60	0.60
Soybean	Protéix	asym	T[air]^{plot}	Asymptotic	2018.00	501.63	512.94		9.62			1.40	1.43				11.27	0.58
Soybean	Protéix	asym	T[air]^{plot}	Asymptotic	2019.00	643.28	655.55	13.49	9.91			2.59	0.97				10.04	0.67
Soybean	Protéix	asym	T[air]^{plot}	Asymptotic	2020.00	498.99	510.38		0.00			0.55	1.65				10.41	0.62
Soybean	Protéix	asym	T[air]^{ref}	Asymptotic	2017.00	563.38	575.10		9.62			1.41	1.46				12.60	0.60
Soybean	Protéix	asym	T[air]^{ref}	Asymptotic	2018.00	501.63	512.94		9.62			1.40	1.43				11.27	0.58
Soybean	Protéix	asym	T[air]^{ref}	Asymptotic	2019.00	643.28	655.55	13.49	9.91			2.59	0.97				10.04	0.67
Soybean	Protéix	asym	T[air]^{ref}	Asymptotic	2020.00	498.99	510.38		0.00			0.55	1.65				10.41	0.62
Soybean	Protéix	bilinear	T[air]^{plot}	Bi-linear	2017.00	567.70	579.42		8.51	0.06	0.30						13.00	0.60
Soybean	Protéix	bilinear	T[air]^{plot}	Bi-linear	2018.00	509.05	520.36		29.90	-0.03	1.46						11.94	0.67
Soybean	Protéix	bilinear	T[air]^{plot}	Bi-linear	2019.00	650.61	662.88	14.54	17.34	0.00	0.93						10.48	0.70
Soybean	Protéix	bilinear	T[air]^{plot}	Bi-linear	2020.00	504.09	515.47		29.81	-0.29	1.67						10.82	0.55
Soybean	Protéix	bilinear	T[air]^{ref}	Bi-linear	2017.00	567.70	579.42		8.51	0.06	0.30						13.00	0.60
Soybean	Protéix	bilinear	T[air]^{ref}	Bi-linear	2018.00	509.05	520.36		29.90	-0.03	1.46						11.94	0.67
Soybean	Protéix	bilinear	T[air]^{ref}	Bi-linear	2019.00	650.61	662.88	14.54	17.34	0.00	0.93						10.48	0.70
Soybean	Protéix	bilinear	T[air]^{ref}	Bi-linear	2020.00	504.09	515.47		29.81	-0.29	1.67						10.82	0.55
Soybean	Protéix	gauss	^{plot}	Gaussian	2017.00	771.64	778.68	37.43								66.60	37.27	0.14
Soybean	Protéix	gauss	^{plot}	Gaussian	2018.00	704.51	711.30	35.82								72.37	35.53	0.16
Soybean	Protéix	gauss	^{plot}	Gaussian	2019.00	777.74	785.10	22.61								58.58	22.66	0.08
Soybean	Protéix	gauss	^{plot}	Gaussian	2020.00	719.25	726.08	36.86								71.37	36.74	0.13
Soybean	Protéix	gauss	^{ref}	Gaussian	2017.00	771.64	778.68	37.43								66.60	37.27	0.14
Soybean	Protéix	gauss	^{ref}	Gaussian	2018.00	704.51	711.30	35.82								72.37	35.53	0.16
Soybean	Protéix	gauss	^{ref}	Gaussian	2019.00	777.74	785.10	22.61								58.58	22.66	0.08
Soybean	Protéix	gauss	^{ref}	Gaussian	2020.00	719.25	726.08	36.86								71.37	36.74	0.13
Soybean	Protéix	linear	T[air]^{plot}	Linear	2017.00	593.21	602.58		-45.88	0.01							15.96	0.73
Soybean	Protéix	linear	T[air]^{plot}	Linear	2018.00	535.39	544.44		-46.51	0.01							15.05	0.75
Soybean	Protéix	linear	T[air]^{plot}	Linear	2019.00	660.48	670.29	16.02	-49.13	0.01							11.26	0.72
Soybean	Protéix	linear	T[air]^{plot}	Linear	2020.00	500.43	509.54		-49.63	0.02							10.72	0.63
Soybean	Protéix	linear	T[air]^{ref}	Linear	2017.00	593.21	602.58		-45.88	0.01							15.96	0.73
Soybean	Protéix	linear	T[air]^{ref}	Linear	2018.00	535.39	544.44		-46.51	0.01							15.05	0.75
Soybean	Protéix	linear	T[air]^{ref}	Linear	2019.00	660.48	670.29	16.02	-49.13	0.01							11.26	0.72
Soybean	Protéix	linear	T[air]^{ref}	Linear	2020.00	500.43	509.54		-49.63	0.02							10.72	0.63
Soybean	Protéix	thermal	T[air]^{plot}	Thermal time	2017.00	565.47	572.50											
Soybean	Protéix	thermal	T[air]^{plot}	Thermal time	2018.00	505.31	512.10											
Soybean	Protéix	thermal	T[air]^{plot}	Thermal time	2019.00	656.04	663.40	13.49										

Soybean	Protéix	thermal	T[air]^{plot}	Thermal time	2020.00	506.67	513.50											
Soybean	Protéix	thermal	T[air]^{ref}	Thermal time	2017.00	565.47	572.50											
Soybean	Protéix	thermal	T[air]^{ref}	Thermal time	2018.00	505.31	512.10											
Soybean	Protéix	thermal	T[air]^{ref}	Thermal time	2019.00	656.04	663.40	13.49										
Soybean	Protéix	thermal	T[air]^{ref}	Thermal time	2020.00	506.67	513.50											
Soybean	Protéix	wang	T[air]^{plot}	Wang-Engel	2017.00	568.10	577.47							26.17	1.46		13.22	0.56
Soybean	Protéix	wang	T[air]^{plot}	Wang-Engel	2018.00	503.03	512.08							26.34	1.44		11.57	0.56
Soybean	Protéix	wang	T[air]^{plot}	Wang-Engel	2019.00	648.52	658.33	14.09						23.01	1.06		10.47	0.67
Soybean	Protéix	wang	T[air]^{plot}	Wang-Engel	2020.00	507.81	516.92							24.35	1.40		11.31	0.51
Soybean	Protéix	wang	T[air]^{ref}	Wang-Engel	2017.00	568.10	577.47							26.17	1.46		13.22	0.56
Soybean	Protéix	wang	T[air]^{ref}	Wang-Engel	2018.00	503.03	512.08							26.34	1.44		11.57	0.56
Soybean	Protéix	wang	T[air]^{ref}	Wang-Engel	2019.00	648.52	658.33	14.09						23.01	1.06		10.47	0.67
Soybean	Protéix	wang	T[air]^{ref}	Wang-Engel	2020.00	507.81	516.92							24.35	1.40		11.31	0.51
Soybean	Tequila	asym	T[air]^{plot}	Asymptotic	2017.00	615.36	627.08	14.05	7.96			0.00	3.05				12.98	0.39
Soybean	Tequila	asym	T[air]^{plot}	Asymptotic	2018.00	574.27	585.58	14.69	7.18			0.00	2.80				13.62	0.39
Soybean	Tequila	asym	T[air]^{plot}	Asymptotic	2019.00	621.03	633.42	11.52	9.38			2.78	0.89				8.12	0.71
Soybean	Tequila	asym	T[air]^{plot}	Asymptotic	2020.00	592.23	603.68	14.66	2.52			0.00	2.31				13.80	0.35
Soybean	Tequila	asym	T[air]^{ref}	Asymptotic	2017.00	615.36	627.08	14.05	7.96			0.00	3.05				12.98	0.39
Soybean	Tequila	asym	T[air]^{ref}	Asymptotic	2018.00	574.27	585.58	14.69	7.18			0.00	2.80				13.62	0.39
Soybean	Tequila	asym	T[air]^{ref}	Asymptotic	2019.00	621.03	633.42	11.52	9.38			2.78	0.89				8.12	0.71
Soybean	Tequila	asym	T[air]^{ref}	Asymptotic	2020.00	592.23	603.68	14.66	2.52			0.00	2.31				13.80	0.35
Soybean	Tequila	bilinear	T[air]^{plot}	Bi-linear	2017.00	613.20	624.92	13.97	14.77	0.08	0.56						12.81	0.41
Soybean	Tequila	bilinear	T[air]^{plot}	Bi-linear	2018.00	577.52	588.84	15.40	30.00	0.20	1.38						13.94	0.44
Soybean	Tequila	bilinear	T[air]^{plot}	Bi-linear	2019.00	629.06	641.45	12.65	8.67	0.01	0.68						8.49	0.74
Soybean	Tequila	bilinear	T[air]^{plot}	Bi-linear	2020.00	591.87	603.32	14.62	29.81	-0.02	1.49						13.76	0.35
Soybean	Tequila	bilinear	T[air]^{ref}	Bi-linear	2017.00	613.20	624.92	13.97	14.77	0.08	0.56						12.81	0.41
Soybean	Tequila	bilinear	T[air]^{ref}	Bi-linear	2018.00	577.52	588.84	15.40	30.00	0.20	1.38						13.94	0.44
Soybean	Tequila	bilinear	T[air]^{ref}	Bi-linear	2019.00	629.06	641.45	12.65	8.67	0.01	0.68						8.49	0.74
Soybean	Tequila	bilinear	T[air]^{ref}	Bi-linear	2020.00	591.87	603.32	14.62	29.81	-0.02	1.49						13.76	0.35
Soybean	Tequila	gauss	^{plot}	Gaussian	2017.00	769.49	776.52	36.83								64.48	36.74	0.09
Soybean	Tequila	gauss	^{plot}	Gaussian	2018.00	702.00	708.79	34.83								71.04	34.90	0.07
Soybean	Tequila	gauss	^{plot}	Gaussian	2019.00	775.13	782.56	20.01								56.39	20.11	-0.01
Soybean	Tequila	gauss	^{plot}	Gaussian	2020.00	726.29	733.16	36.03								67.94	35.98	0.11
Soybean	Tequila	gauss	^{ref}	Gaussian	2017.00	769.49	776.52	36.83								64.48	36.74	0.09
Soybean	Tequila	gauss	^{ref}	Gaussian	2018.00	702.00	708.79	34.83								71.04	34.90	0.07
Soybean	Tequila	gauss	^{ref}	Gaussian	2019.00	775.13	782.56	20.01								56.39	20.11	-0.01
Soybean	Tequila	gauss	^{ref}	Gaussian	2020.00	726.29	733.16	36.03								67.94	35.98	0.11
Soybean	Tequila	linear	T[air]^{plot}	Linear	2017.00	652.51	661.88	20.27	-45.20	0.01							16.96	0.57
Soybean	Tequila	linear	T[air]^{plot}	Linear	2018.00	603.32	612.37	20.45	-45.06	0.01							17.16	0.57
Soybean	Tequila	linear	T[air]^{plot}	Linear	2019.00	627.21	637.12	12.91	-51.02	0.01							8.50	0.76
Soybean	Tequila	linear	T[air]^{plot}	Linear	2020.00	598.08	607.24	15.56	-46.80	0.02							14.64	0.37
Soybean	Tequila	linear	T[air]^{ref}	Linear	2017.00	652.51	661.88	20.27	-45.20	0.01							16.96	0.57
Soybean	Tequila	linear	T[air]^{ref}	Linear	2018.00	603.32	612.37	20.45	-45.06	0.01							17.16	0.57
Soybean	Tequila	linear	T[air]^{ref}	Linear	2019.00	627.21	637.12	12.91	-51.02	0.01							8.50	0.76
Soybean	Tequila	linear	T[air]^{ref}	Linear	2020.00	598.08	607.24	15.56	-46.80	0.02							14.64	0.37
Soybean	Tequila	thermal	T[air]^{plot}	Thermal time	2017.00	617.94	624.98	15.42										
Soybean	Tequila	thermal	T[air]^{plot}	Thermal time	2018.00	574.24	581.03	15.62										
Soybean	Tequila	thermal	T[air]^{plot}	Thermal time	2019.00	642.98	650.41	11.11										
Soybean	Tequila	thermal	T[air]^{plot}	Thermal time	2020.00	588.07	594.94	14.66										
Soybean	Tequila	thermal	T[air]^{ref}	Thermal time	2017.00	617.94	624.98	15.42										
Soybean	Tequila	thermal	T[air]^{ref}	Thermal time	2018.00	574.24	581.03	15.62										
Soybean	Tequila	thermal	T[air]^{ref}	Thermal time	2019.00	642.98	650.41	11.11										

Soybean	Tequila	thermal	T[air]^{ref}	Thermal time	2020.00	588.07	594.94	14.66										
Soybean	Tequila	wang	T[air]^{plot}	Wang-Engel	2017.00	619.95	629.33	14.45						27.32	1.59		13.56	0.36
Soybean	Tequila	wang	T[air]^{plot}	Wang-Engel	2018.00	574.38	583.43	14.88						26.86	1.48		13.83	0.38
Soybean	Tequila	wang	T[air]^{plot}	Wang-Engel	2019.00	618.35	628.26	12.02						21.52	0.97		8.08	0.74
Soybean	Tequila	wang	T[air]^{plot}	Wang-Engel	2020.00	596.49	605.65	15.11						26.04	1.43		14.41	0.32
Soybean	Tequila	wang	T[air]^{ref}	Wang-Engel	2017.00	619.95	629.33	14.45						27.32	1.59		13.56	0.36
Soybean	Tequila	wang	T[air]^{ref}	Wang-Engel	2018.00	574.38	583.43	14.88						26.86	1.48		13.83	0.38
Soybean	Tequila	wang	T[air]^{ref}	Wang-Engel	2019.00	618.35	628.26	12.02						21.52	0.97		8.08	0.74
Soybean	Tequila	wang	T[air]^{ref}	Wang-Engel	2020.00	596.49	605.65	15.11						26.04	1.43		14.41	0.32
Soybean	Toutatis	asym	T[air]^{plot}	Asymptotic	2017.00	590.66	602.38		9.22			1.05	1.69				13.65	0.52
Soybean	Toutatis	asym	T[air]^{plot}	Asymptotic	2018.00	538.98	550.29		9.62			1.31	1.45				13.31	0.47
Soybean	Toutatis	asym	T[air]^{plot}	Asymptotic	2019.00	649.01	661.29	13.80	10.13			2.73	0.96				10.38	0.66
Soybean	Toutatis	asym	T[air]^{plot}	Asymptotic	2020.00	543.89	555.27		0.00			0.44	1.68				13.00	0.51
Soybean	Toutatis	asym	T[air]^{ref}	Asymptotic	2017.00	590.66	602.38		9.22			1.05	1.69				13.65	0.52
Soybean	Toutatis	asym	T[air]^{ref}	Asymptotic	2018.00	538.98	550.29		9.62			1.31	1.45				13.31	0.47
Soybean	Toutatis	asym	T[air]^{ref}	Asymptotic	2019.00	649.01	661.29	13.80	10.13			2.73	0.96				10.38	0.66
Soybean	Toutatis	asym	T[air]^{ref}	Asymptotic	2020.00	543.89	555.27		0.00			0.44	1.68				13.00	0.51
Soybean	Toutatis	bilinear	T[air]^{plot}	Bi-linear	2017.00	592.55	604.27		9.13	0.06	0.30						13.82	0.53
Soybean	Toutatis	bilinear	T[air]^{plot}	Bi-linear	2018.00	544.38	555.69		30.00	0.00	1.42						13.87	0.51
Soybean	Toutatis	bilinear	T[air]^{plot}	Bi-linear	2019.00	654.31	666.58	14.58	17.57	-0.00	0.94						10.71	0.68
Soybean	Toutatis	bilinear	T[air]^{plot}	Bi-linear	2020.00	545.91	557.30		29.91	-0.30	1.62						13.20	0.48
Soybean	Toutatis	bilinear	T[air]^{ref}	Bi-linear	2017.00	592.55	604.27		9.13	0.06	0.30						13.82	0.53
Soybean	Toutatis	bilinear	T[air]^{ref}	Bi-linear	2018.00	544.38	555.69		30.00	0.00	1.42						13.87	0.51
Soybean	Toutatis	bilinear	T[air]^{ref}	Bi-linear	2019.00	654.31	666.58	14.58	17.57	-0.00	0.94						10.71	0.68
Soybean	Toutatis	bilinear	T[air]^{ref}	Bi-linear	2020.00	545.91	557.30		29.91	-0.30	1.62						13.20	0.48
Soybean	Toutatis	gauss	^{plot}	Gaussian	2017.00	770.66	777.69	37.09								66.85	37.03	0.11
Soybean	Toutatis	gauss	^{plot}	Gaussian	2018.00	700.48	707.26	34.52								72.32	34.52	0.09
Soybean	Toutatis	gauss	^{plot}	Gaussian	2019.00	770.44	777.80	21.61								58.54	21.71	0.04
Soybean	Toutatis	gauss	^{plot}	Gaussian	2020.00	716.89	723.72	36.21								71.22	36.14	0.12
Soybean	Toutatis	gauss	^{ref}	Gaussian	2017.00	770.66	777.69	37.09								66.85	37.03	0.11
Soybean	Toutatis	gauss	^{ref}	Gaussian	2018.00	700.48	707.26	34.52								72.32	34.52	0.09
Soybean	Toutatis	gauss	^{ref}	Gaussian	2019.00	770.44	777.80	21.61								58.54	21.71	0.04
Soybean	Toutatis	gauss	^{ref}	Gaussian	2020.00	716.89	723.72	36.21								71.22	36.14	0.12
Soybean	Toutatis	linear	T[air]^{plot}	Linear	2017.00	619.06	628.44		-45.17	0.01							17.00	0.63
Soybean	Toutatis	linear	T[air]^{plot}	Linear	2018.00	565.73	574.78		-45.38	0.01							16.70	0.61
Soybean	Toutatis	linear	T[air]^{plot}	Linear	2019.00	661.24	671.06	15.73	-49.09	0.01							11.31	0.70
Soybean	Toutatis	linear	T[air]^{plot}	Linear	2020.00	543.92	553.02		-48.22	0.02							13.23	0.52
Soybean	Toutatis	linear	T[air]^{ref}	Linear	2017.00	619.06	628.44		-45.17	0.01							17.00	0.63
Soybean	Toutatis	linear	T[air]^{ref}	Linear	2018.00	565.73	574.78		-45.38	0.01							16.70	0.61
Soybean	Toutatis	linear	T[air]^{ref}	Linear	2019.00	661.24	671.06	15.73	-49.09	0.01							11.31	0.70
Soybean	Toutatis	linear	T[air]^{ref}	Linear	2020.00	543.92	553.02		-48.22	0.02							13.23	0.52
Soybean	Toutatis	thermal	T[air]^{plot}	Thermal time	2017.00	591.58	598.62											
Soybean	Toutatis	thermal	T[air]^{plot}	Thermal time	2018.00	540.44	547.23											
Soybean	Toutatis	thermal	T[air]^{plot}	Thermal time	2019.00	661.74	669.10	14.10										
Soybean	Toutatis	thermal	T[air]^{plot}	Thermal time	2020.00	546.23	553.06											
Soybean	Toutatis	thermal	T[air]^{ref}	Thermal time	2017.00	591.58	598.62											
Soybean	Toutatis	thermal	T[air]^{ref}	Thermal time	2018.00	540.44	547.23											
Soybean	Toutatis	thermal	T[air]^{ref}	Thermal time	2019.00	661.74	669.10	14.10										
Soybean	Toutatis	thermal	T[air]^{ref}	Thermal time	2020.00	546.23	553.06											
Soybean	Toutatis	wang	T[air]^{plot}	Wang-Engel	2017.00	592.09	601.47							26.48	1.49		13.97	0.50
Soybean	Toutatis	wang	T[air]^{plot}	Wang-Engel	2018.00	537.85	546.90							26.46	1.44		13.40	0.48
Soybean	Toutatis	wang	T[air]^{plot}	Wang-Engel	2019.00	651.83	661.64	14.20						22.91	1.05		10.68	0.66

Soybean	Toutatis	wang	T[air]^{plot}	Wang-Engel	2020.00	549.34	558.45							24.56	1.36		13.75	0.44
Soybean	Toutatis	wang	T[air]^{ref}	Wang-Engel	2017.00	592.09	601.47							26.48	1.49		13.97	0.50
Soybean	Toutatis	wang	T[air]^{ref}	Wang-Engel	2018.00	537.85	546.90							26.46	1.44		13.40	0.48
Soybean	Toutatis	wang	T[air]^{ref}	Wang-Engel	2019.00	651.83	661.64	14.20						22.91	1.05		10.68	0.66
Soybean	Toutatis	wang	T[air]^{ref}	Wang-Engel	2020.00	549.34	558.45							24.56	1.36		13.75	0.44
Wheat	ALL	asym	T[air]^{plot}	Asymptotic	2015.00	9930.90	9956.75		4.30			2.28	0.68				12.76	0.54
Wheat	ALL	asym	T[air]^{plot}	Asymptotic	2016.00	9484.15	9509.76		5.60			4.09	0.58				12.96	0.54
Wheat	ALL	asym	T[air]^{plot}	Asymptotic	2017.00	9662.51	9688.28	12.50	5.54			5.68	0.57				10.57	0.54
Wheat	ALL	asym	T[air]^{plot}	Asymptotic	2018.00	9651.99	9677.61		6.12			9.84	0.60				13.73	0.51
Wheat	ALL	asym	T[air]^{plot}	Asymptotic	2019.00	8819.94	8845.14		3.30			1.35	0.85				13.67	0.52
Wheat	ALL	asym	T[air]^{plot}	Asymptotic	2021.00	9739.97	9765.63		5.70			6.32	0.58				13.76	0.52
Wheat	ALL	asym	T[air]^{ref}	Asymptotic	2015.00	10267.37	10293.21		3.30			1.91	0.72				12.70	0.53
Wheat	ALL	asym	T[air]^{ref}	Asymptotic	2016.00	9867.19	9892.80		5.24			8.27	0.56				13.13	0.55
Wheat	ALL	asym	T[air]^{ref}	Asymptotic	2017.00	9650.23	9676.00		5.30			6.05	0.57				10.62	0.52
Wheat	ALL	asym	T[air]^{ref}	Asymptotic	2018.00	10031.97	10057.59	16.05	5.70			10.15	0.59				13.72	0.52
Wheat	ALL	asym	T[air]^{ref}	Asymptotic	2019.00	9237.88	9263.08		6.80			4.86	0.62				14.04	0.52
Wheat	ALL	asym	T[air]^{ref}	Asymptotic	2021.00	10058.32	10083.98		2.84			1.65	0.73				13.56	0.51
Wheat	ALL	bilinear	T[air]^{plot}	Bi-linear	2015.00	9929.92	9955.76		10.21	0.02	0.55						12.76	0.57
Wheat	ALL	bilinear	T[air]^{plot}	Bi-linear	2016.00	9490.10	9515.71		9.96	0.02	0.51						12.99	0.56
Wheat	ALL	bilinear	T[air]^{plot}	Bi-linear	2017.00	9619.08	9644.85	12.17	7.84	0.01	0.50						10.39	0.52
Wheat	ALL	bilinear	T[air]^{plot}	Bi-linear	2018.00	9657.31	9682.93		9.16	0.02	0.50						13.75	0.53
Wheat	ALL	bilinear	T[air]^{plot}	Bi-linear	2019.00	8839.70	8864.90		9.10	0.03	0.46						13.80	0.53
Wheat	ALL	bilinear	T[air]^{plot}	Bi-linear	2021.00	9702.40	9728.06		10.28	0.02	0.52						13.52	0.55
Wheat	ALL	bilinear	T[air]^{ref}	Bi-linear	2015.00	10284.04	10309.89		12.26	0.01	0.65						12.78	0.54
Wheat	ALL	bilinear	T[air]^{ref}	Bi-linear	2016.00	9856.10	9881.71		10.73	0.01	0.56						13.08	0.54
Wheat	ALL	bilinear	T[air]^{ref}	Bi-linear	2017.00	9610.38	9636.15		7.70	0.01	0.50						10.44	0.52
Wheat	ALL	bilinear	T[air]^{ref}	Bi-linear	2018.00	10040.61	10066.23	16.01	11.21	0.01	0.60						13.78	0.51
Wheat	ALL	bilinear	T[air]^{ref}	Bi-linear	2019.00	9188.95	9214.15		12.80	0.02	0.62						13.75	0.50
Wheat	ALL	bilinear	T[air]^{ref}	Bi-linear	2021.00	10066.30	10091.96		11.10	0.01	0.57						13.60	0.52
Wheat	ALL	gauss	^{plot}	Gaussian	2015.00	12239.83	12255.34	28.91								44.27	26.94	0.36
Wheat	ALL	gauss	^{plot}	Gaussian	2016.00	11647.19	11662.56	27.98								45.21	26.75	0.30
Wheat	ALL	gauss	^{plot}	Gaussian	2017.00	11996.25	12011.71	27.41								45.68	26.37	0.28
Wheat	ALL	gauss	^{plot}	Gaussian	2018.00	11901.97	11917.34	30.87								48.00	29.19	0.33
Wheat	ALL	gauss	^{plot}	Gaussian	2019.00	10781.22	10796.34	29.01								47.74	27.42	0.33
Wheat	ALL	gauss	^{plot}	Gaussian	2021.00	11971.80	11987.20	30.44								48.03	28.89	0.32
Wheat	ALL	gauss	^{ref}	Gaussian	2015.00	12239.83	12255.34	28.91								44.27	26.94	0.36
Wheat	ALL	gauss	^{ref}	Gaussian	2016.00	11647.19	11662.56	27.98								45.21	26.75	0.30
Wheat	ALL	gauss	^{ref}	Gaussian	2017.00	11996.25	12011.71	27.41								45.68	26.37	0.28
Wheat	ALL	gauss	^{ref}	Gaussian	2018.00	11901.97	11917.34	30.87								48.00	29.19	0.33
Wheat	ALL	gauss	^{ref}	Gaussian	2019.00	10781.22	10796.34	29.01								47.74	27.42	0.33
Wheat	ALL	gauss	^{ref}	Gaussian	2021.00	11971.80	11987.20	30.44								48.03	28.89	0.32
Wheat	ALL	linear	T[air]^{plot}	Linear	2015.00	10283.97	10304.64		-46.68	0.01							14.81	0.59
Wheat	ALL	linear	T[air]^{plot}	Linear	2016.00	9768.30	9788.78		-46.76	0.01							14.70	0.60
Wheat	ALL	linear	T[air]^{plot}	Linear	2017.00	9684.95	9705.56	12.84	-49.51	0.01							10.69	0.56
Wheat	ALL	linear	T[air]^{plot}	Linear	2018.00	9961.79	9982.29		-46.04	0.01							15.74	0.57
Wheat	ALL	linear	T[air]^{plot}	Linear	2019.00	9171.79	9191.95		-45.73	0.01							16.19	0.56
Wheat	ALL	linear	T[air]^{plot}	Linear	2021.00	10012.74	10033.27		-46.18	0.01							15.53	0.57
Wheat	ALL	linear	T[air]^{ref}	Linear	2015.00	10650.31	10670.99		-46.66	0.01							14.83	0.58
Wheat	ALL	linear	T[air]^{ref}	Linear	2016.00	10140.44	10160.93		-46.71	0.01							14.76	0.58
Wheat	ALL	linear	T[air]^{ref}	Linear	2017.00	9664.89	9685.51		-49.51	0.01							10.71	0.56
Wheat	ALL	linear	T[air]^{ref}	Linear	2018.00	10353.76	10374.26	18.90	-46.04	0.01							15.74	0.56
Wheat	ALL	linear	T[air]^{ref}	Linear	2019.00	9538.10	9558.25		-45.75	0.01							16.16	0.55

Wheat	ALL	linear	T[air]^{ref}	Linear	2021.00	10380.91	10401.44		-46.17	0.01							15.54	0.57
Wheat	ALL	thermal	T[air]^{plot}	Thermal time	2015.00	10030.19	10045.70											
Wheat	ALL	thermal	T[air]^{plot}	Thermal time	2016.00	9604.03	9619.40											
Wheat	ALL	thermal	T[air]^{plot}	Thermal time	2017.00	9930.44	9945.91	13.59										
Wheat	ALL	thermal	T[air]^{plot}	Thermal time	2018.00	9743.47	9758.84											
Wheat	ALL	thermal	T[air]^{plot}	Thermal time	2019.00	8871.13	8886.25											
Wheat	ALL	thermal	T[air]^{plot}	Thermal time	2021.00	9782.61	9798.01											
Wheat	ALL	thermal	T[air]^{ref}	Thermal time	2015.00	10448.21	10463.71											
Wheat	ALL	thermal	T[air]^{ref}	Thermal time	2016.00	10019.99	10035.36											
Wheat	ALL	thermal	T[air]^{ref}	Thermal time	2017.00	9956.52	9971.98											
Wheat	ALL	thermal	T[air]^{ref}	Thermal time	2018.00	10176.97	10192.35	16.49										
Wheat	ALL	thermal	T[air]^{ref}	Thermal time	2019.00	9243.84	9258.95											
Wheat	ALL	thermal	T[air]^{ref}	Thermal time	2021.00	10190.36	10205.76											
Wheat	ALL	wang	T[air]^{plot}	Wang-Engel	2015.00	10106.91	10127.59							16.79	0.81		13.70	0.50
Wheat	ALL	wang	T[air]^{plot}	Wang-Engel	2016.00	9696.76	9717.25							16.82	0.74		14.18	0.53
Wheat	ALL	wang	T[air]^{plot}	Wang-Engel	2017.00	9932.34	9952.96	13.56						15.36	0.68		11.75	0.50
Wheat	ALL	wang	T[air]^{plot}	Wang-Engel	2018.00	9809.89	9830.39							16.83	0.78		14.67	0.50
Wheat	ALL	wang	T[air]^{plot}	Wang-Engel	2019.00	9007.62	9027.78							17.98	0.86		14.91	0.50
Wheat	ALL	wang	T[air]^{plot}	Wang-Engel	2021.00	9904.98	9925.51							17.11	0.78		14.75	0.51
Wheat	ALL	wang	T[air]^{ref}	Wang-Engel	2015.00	10381.41	10402.08							16.71	0.81		13.28	0.52
Wheat	ALL	wang	T[air]^{ref}	Wang-Engel	2016.00	9986.93	10007.42							16.72	0.74		13.80	0.55
Wheat	ALL	wang	T[air]^{ref}	Wang-Engel	2017.00	9905.51	9926.12							15.15	0.68		11.73	0.50
Wheat	ALL	wang	T[air]^{ref}	Wang-Engel	2018.00	10139.81	10160.31	16.74						16.74	0.78		14.34	0.52
Wheat	ALL	wang	T[air]^{ref}	Wang-Engel	2019.00	9278.99	9299.15							17.85	0.85		14.32	0.50
Wheat	ALL	wang	T[air]^{ref}	Wang-Engel	2021.00	10198.94	10219.47							16.99	0.78		14.35	0.52
Wheat	CH CLARO	asym	T[air]^{plot}	Asymptotic	2015.00	902.72	916.61		5.20			4.71	0.54				12.15	0.54
Wheat	CH CLARO	asym	T[air]^{plot}	Asymptotic	2016.00	873.76	887.48		5.40			5.70	0.55				12.27	0.52
Wheat	CH CLARO	asym	T[air]^{plot}	Asymptotic	2017.00	849.30	863.11	9.98	5.33			11.57	0.56				9.01	0.45
Wheat	CH CLARO	asym	T[air]^{plot}	Asymptotic	2018.00	883.95	897.71		6.13			11.26	0.59				12.40	0.44
Wheat	CH CLARO	asym	T[air]^{plot}	Asymptotic	2019.00	779.22	792.34		6.36			8.69	0.58				12.76	0.52
Wheat	CH CLARO	asym	T[air]^{plot}	Asymptotic	2021.00	885.27	899.03		5.61			9.58	0.57				12.47	0.52
Wheat	CH CLARO	asym	T[air]^{ref}	Asymptotic	2015.00	932.37	946.27	14.42	5.12			6.65	0.54				12.06	0.56
Wheat	CH CLARO	asym	T[air]^{ref}	Asymptotic	2016.00	914.86	928.59	14.97	6.79			7.47	0.59				12.84	0.51
Wheat	CH CLARO	asym	T[air]^{ref}	Asymptotic	2017.00	848.46	862.27	9.90	5.20			6.97	0.56				8.99	0.43
Wheat	CH CLARO	asym	T[air]^{ref}	Asymptotic	2018.00	910.33	924.10	14.01	5.10			4.82	0.58				12.13	0.51
Wheat	CH CLARO	asym	T[air]^{ref}	Asymptotic	2019.00	805.94	819.07	15.07	5.40			4.65	0.58				12.44	0.58
Wheat	CH CLARO	asym	T[air]^{ref}	Asymptotic	2021.00	915.19	928.96	14.69	5.25			6.99	0.57				12.39	0.55
Wheat	CH CLARO	bilinear	T[air]^{plot}	Bi-linear	2015.00	905.44	919.33		8.23	0.01	0.49						12.29	0.55
Wheat	CH CLARO	bilinear	T[air]^{plot}	Bi-linear	2016.00	872.50	886.22		7.31	0.02	0.45						12.20	0.54
Wheat	CH CLARO	bilinear	T[air]^{plot}	Bi-linear	2017.00	834.64	848.45	9.39	6.60	0.00	0.52						8.46	0.45
Wheat	CH CLARO	bilinear	T[air]^{plot}	Bi-linear	2018.00	885.33	899.10		9.10	0.01	0.54						12.48	0.48
Wheat	CH CLARO	bilinear	T[air]^{plot}	Bi-linear	2019.00	780.80	793.92		8.80	0.02	0.49						12.86	0.54
Wheat	CH CLARO	bilinear	T[air]^{plot}	Bi-linear	2021.00	887.31	901.08		9.00	0.01	0.52						12.59	0.55
Wheat	CH CLARO	bilinear	T[air]^{ref}	Bi-linear	2015.00	935.61	949.51	14.75	8.63	0.01	0.51						12.22	0.56
Wheat	CH CLARO	bilinear	T[air]^{ref}	Bi-linear	2016.00	902.67	916.39	14.62	7.35	0.01	0.46						12.14	0.55
Wheat	CH CLARO	bilinear	T[air]^{ref}	Bi-linear	2017.00	835.87	849.68	9.40	6.50	0.00	0.52						8.51	0.44
Wheat	CH CLARO	bilinear	T[air]^{ref}	Bi-linear	2018.00	914.75	928.51	14.30	9.03	0.01	0.54						12.36	0.51
Wheat	CH CLARO	bilinear	T[air]^{ref}	Bi-linear	2019.00	808.61	821.73	14.88	10.51	0.01	0.55						12.61	0.54
Wheat	CH CLARO	bilinear	T[air]^{ref}	Bi-linear	2021.00	916.99	930.76	14.90	9.00	0.01	0.53						12.48	0.56
Wheat	CH CLARO	gauss	^{plot}	Gaussian	2015.00	1100.09	1108.43	27.65								40.66	24.95	0.44
Wheat	CH CLARO	gauss	^{plot}	Gaussian	2016.00	1080.43	1088.67	28.49								42.22	26.94	0.33
Wheat	CH CLARO	gauss	^{plot}	Gaussian	2017.00	1096.87	1105.16	27.54								42.61	26.66	0.27

Wheat	CH CLARO	gauss	^{plot}	Gaussian	2018.00	1108.66	1116.92	30.88							45.10	29.23	0.33
Wheat	CH CLARO	gauss	^{plot}	Gaussian	2019.00	967.50	975.37	29.65							45.65	28.25	0.32
Wheat	CH CLARO	gauss	^{plot}	Gaussian	2021.00	1109.13	1117.39	30.83							45.28	29.29	0.32
Wheat	CH CLARO	gauss	^{ref}	Gaussian	2015.00	1100.09	1108.43	27.65							40.66	24.95	0.44
Wheat	CH CLARO	gauss	^{ref}	Gaussian	2016.00	1080.43	1088.67	28.49							42.22	26.94	0.33
Wheat	CH CLARO	gauss	^{ref}	Gaussian	2017.00	1096.87	1105.16	27.54							42.61	26.66	0.27
Wheat	CH CLARO	gauss	^{ref}	Gaussian	2018.00	1108.66	1116.92	30.88							45.10	29.23	0.33
Wheat	CH CLARO	gauss	^{ref}	Gaussian	2019.00	967.50	975.37	29.65							45.65	28.25	0.32
Wheat	CH CLARO	gauss	^{ref}	Gaussian	2021.00	1109.13	1117.39	30.83							45.28	29.29	0.32
Wheat	CH CLARO	linear	T[air]^{plot}	Linear	2015.00	908.74	919.86		-7.82	0.02						12.57	0.58
Wheat	CH CLARO	linear	T[air]^{plot}	Linear	2016.00	889.91	900.89		-47.66	0.01						13.39	0.60
Wheat	CH CLARO	linear	T[air]^{plot}	Linear	2017.00	830.30	841.35	9.80	-51.11	0.01						8.38	0.53
Wheat	CH CLARO	linear	T[air]^{plot}	Linear	2018.00	909.67	920.69		-47.15	0.01						14.13	0.56
Wheat	CH CLARO	linear	T[air]^{plot}	Linear	2019.00	804.00	814.50		-46.73	0.01						14.73	0.59
Wheat	CH CLARO	linear	T[air]^{plot}	Linear	2021.00	907.64	918.66		-47.24	0.01						14.00	0.60
Wheat	CH CLARO	linear	T[air]^{ref}	Linear	2015.00	940.27	951.38	15.19	-8.62	0.02						12.57	0.57
Wheat	CH CLARO	linear	T[air]^{ref}	Linear	2016.00	920.73	931.71	16.70	-47.71	0.01						13.31	0.61
Wheat	CH CLARO	linear	T[air]^{ref}	Linear	2017.00	830.01	841.06	9.78	-51.11	0.01						8.37	0.53
Wheat	CH CLARO	linear	T[air]^{ref}	Linear	2018.00	940.31	951.32	16.91	-47.23	0.01						14.01	0.57
Wheat	CH CLARO	linear	T[air]^{ref}	Linear	2019.00	814.07	824.57	15.07	-3.81	0.03						13.09	0.50
Wheat	CH CLARO	linear	T[air]^{ref}	Linear	2021.00	938.14	949.15	17.26	-47.32	0.01						13.88	0.61
Wheat	CH CLARO	thermal	T[air]^{plot}	Thermal time	2015.00	920.06	928.39										
Wheat	CH CLARO	thermal	T[air]^{plot}	Thermal time	2016.00	884.47	892.70										
Wheat	CH CLARO	thermal	T[air]^{plot}	Thermal time	2017.00	894.71	903.00	12.24									
Wheat	CH CLARO	thermal	T[air]^{plot}	Thermal time	2018.00	899.92	908.18										
Wheat	CH CLARO	thermal	T[air]^{plot}	Thermal time	2019.00	785.94	793.82										
Wheat	CH CLARO	thermal	T[air]^{plot}	Thermal time	2021.00	899.17	907.43										
Wheat	CH CLARO	thermal	T[air]^{ref}	Thermal time	2015.00	954.33	962.67	15.49									
Wheat	CH CLARO	thermal	T[air]^{ref}	Thermal time	2016.00	918.14	926.37	15.01									
Wheat	CH CLARO	thermal	T[air]^{ref}	Thermal time	2017.00	898.59	906.88	12.37									
Wheat	CH CLARO	thermal	T[air]^{ref}	Thermal time	2018.00	933.70	941.96	14.99									
Wheat	CH CLARO	thermal	T[air]^{ref}	Thermal time	2019.00	815.46	823.34	14.72									
Wheat	CH CLARO	thermal	T[air]^{ref}	Thermal time	2021.00	932.70	940.96	15.55									
Wheat	CH CLARO	wang	T[air]^{plot}	Wang-Engel	2015.00	917.21	928.32						15.02	0.64		13.05	0.50
Wheat	CH CLARO	wang	T[air]^{plot}	Wang-Engel	2016.00	888.77	899.75						15.70	0.66		13.26	0.48
Wheat	CH CLARO	wang	T[air]^{plot}	Wang-Engel	2017.00	871.18	882.23	10.68					12.86	0.61		9.99	0.37
Wheat	CH CLARO	wang	T[air]^{plot}	Wang-Engel	2018.00	891.66	902.67						15.77	0.71		12.96	0.41
Wheat	CH CLARO	wang	T[air]^{plot}	Wang-Engel	2019.00	790.59	801.09						16.90	0.75		13.67	0.46
Wheat	CH CLARO	wang	T[air]^{plot}	Wang-Engel	2021.00	899.65	910.66						15.83	0.70		13.43	0.47
Wheat	CH CLARO	wang	T[air]^{ref}	Wang-Engel	2015.00	943.02	954.13	15.31					15.22	0.65		12.72	0.56
Wheat	CH CLARO	wang	T[air]^{ref}	Wang-Engel	2016.00	914.66	925.64	15.48					15.81	0.67		12.91	0.55
Wheat	CH CLARO	wang	T[air]^{ref}	Wang-Engel	2017.00	870.50	881.55	10.66					12.87	0.61		9.96	0.37
Wheat	CH CLARO	wang	T[air]^{ref}	Wang-Engel	2018.00	919.33	930.34	14.60					15.71	0.70		12.72	0.50
Wheat	CH CLARO	wang	T[air]^{ref}	Wang-Engel	2019.00	812.82	823.32	15.09					17.02	0.76		13.01	0.52
Wheat	CH CLARO	wang	T[air]^{ref}	Wang-Engel	2021.00	925.50	936.52	15.31					15.94	0.71		13.07	0.53
Wheat	CH NARA	asym	T[air]^{plot}	Asymptotic	2015.00	713.10	726.02		5.61		9.33	0.48				10.60	0.59
Wheat	CH NARA	asym	T[air]^{plot}	Asymptotic	2016.00	713.10	726.02		5.61		9.33	0.48				10.60	0.59
Wheat	CH NARA	asym	T[air]^{plot}	Asymptotic	2017.00	531.81	543.47	9.25	4.49		4.20	0.47				7.84	0.56
Wheat	CH NARA	asym	T[air]^{plot}	Asymptotic	2018.00	571.73	583.51		5.61		9.58	0.50				11.34	0.56
Wheat	CH NARA	asym	T[air]^{plot}	Asymptotic	2019.00	477.02	487.89		5.61		6.44	0.47				11.86	0.62
Wheat	CH NARA	asym	T[air]^{plot}	Asymptotic	2021.00	544.54	556.13		5.60		9.59	0.45				11.01	0.64
Wheat	CH NARA	asym	T[air]^{ref}	Asymptotic	2015.00	743.94	756.87	13.02	5.12		4.79	0.48				10.64	0.56

Wheat	CH NARA	asym	T[air]^{ref}	Asymptotic	2016.00	743.94	756.87	13.02	5.12			4.79	0.48		10.64	0.56
Wheat	CH NARA	asym	T[air]^{ref}	Asymptotic	2017.00	530.39	542.04	9.26	0.00			2.43	0.47		7.77	0.58
Wheat	CH NARA	asym	T[air]^{ref}	Asymptotic	2018.00	600.79	612.58	13.59	5.71			10.40	0.52		11.22	0.53
Wheat	CH NARA	asym	T[air]^{ref}	Asymptotic	2019.00	506.17	517.04	14.21	4.90			2.64	0.54		11.59	0.54
Wheat	CH NARA	asym	T[air]^{ref}	Asymptotic	2021.00	575.82	587.41	13.80	5.12			4.68	0.46		11.07	0.58
Wheat	CH NARA	bilinear	T[air]^{plot}	Bi-linear	2015.00	721.10	734.02		8.80	0.00	0.45				11.07	0.61
Wheat	CH NARA	bilinear	T[air]^{plot}	Bi-linear	2016.00	721.10	734.02		8.80	0.00	0.45				11.07	0.61
Wheat	CH NARA	bilinear	T[air]^{plot}	Bi-linear	2017.00	525.84	537.49	9.01	7.37	-0.01	0.50				7.54	0.59
Wheat	CH NARA	bilinear	T[air]^{plot}	Bi-linear	2018.00	579.77	591.55		10.03	0.00	0.50				11.98	0.58
Wheat	CH NARA	bilinear	T[air]^{plot}	Bi-linear	2019.00	480.91	491.79		8.80	0.02	0.38				12.25	0.63
Wheat	CH NARA	bilinear	T[air]^{plot}	Bi-linear	2021.00	547.06	558.65		10.47	0.00	0.46				11.21	0.66
Wheat	CH NARA	bilinear	T[air]^{ref}	Bi-linear	2015.00	750.55	763.48	13.67	10.51	-0.00	0.51				11.01	0.58
Wheat	CH NARA	bilinear	T[air]^{ref}	Bi-linear	2016.00	750.55	763.48	13.67	10.51	-0.00	0.51				11.01	0.58
Wheat	CH NARA	bilinear	T[air]^{ref}	Bi-linear	2017.00	525.64	537.30	8.99	7.18	-0.01	0.51				7.53	0.59
Wheat	CH NARA	bilinear	T[air]^{ref}	Bi-linear	2018.00	607.96	619.74	14.59	12.22	-0.01	0.59				11.75	0.57
Wheat	CH NARA	bilinear	T[air]^{ref}	Bi-linear	2019.00	510.08	520.95	14.97	12.60	0.00	0.54				12.04	0.58
Wheat	CH NARA	bilinear	T[air]^{ref}	Bi-linear	2021.00	576.72	588.30	14.31	11.13	-0.01	0.50				11.14	0.62
Wheat	CH NARA	gauss	^{plot}	Gaussian	2015.00	872.09	879.84	22.48						35.59	21.02	0.37
Wheat	CH NARA	gauss	^{plot}	Gaussian	2016.00	872.09	879.84	22.48						35.59	21.02	0.37
Wheat	CH NARA	gauss	^{plot}	Gaussian	2017.00	664.66	671.65	20.16						34.42	19.53	0.27
Wheat	CH NARA	gauss	^{plot}	Gaussian	2018.00	705.39	712.46	24.42						36.28	22.70	0.38
Wheat	CH NARA	gauss	^{plot}	Gaussian	2019.00	568.80	575.32	21.16						36.11	19.65	0.39
Wheat	CH NARA	gauss	^{plot}	Gaussian	2021.00	672.36	679.32	23.64						35.41	21.84	0.40
Wheat	CH NARA	gauss	^{ref}	Gaussian	2015.00	872.09	879.84	22.48						35.59	21.02	0.37
Wheat	CH NARA	gauss	^{ref}	Gaussian	2016.00	872.09	879.84	22.48						35.59	21.02	0.37
Wheat	CH NARA	gauss	^{ref}	Gaussian	2017.00	664.66	671.65	20.16						34.42	19.53	0.27
Wheat	CH NARA	gauss	^{ref}	Gaussian	2018.00	705.39	712.46	24.42						36.28	22.70	0.38
Wheat	CH NARA	gauss	^{ref}	Gaussian	2019.00	568.80	575.32	21.16						36.11	19.65	0.39
Wheat	CH NARA	gauss	^{ref}	Gaussian	2021.00	672.36	679.32	23.64						35.41	21.84	0.40
Wheat	CH NARA	linear	T[air]^{plot}	Linear	2015.00	736.42	746.76		-48.48	0.01					12.19	0.66
Wheat	CH NARA	linear	T[air]^{plot}	Linear	2016.00	736.42	746.76		-48.48	0.01					12.19	0.66
Wheat	CH NARA	linear	T[air]^{plot}	Linear	2017.00	527.39	536.71	9.58	-51.56	0.01					7.72	0.62
Wheat	CH NARA	linear	T[air]^{plot}	Linear	2018.00	595.17	604.60		-47.54	0.01					13.57	0.65
Wheat	CH NARA	linear	T[air]^{plot}	Linear	2019.00	481.37	490.06		-2.09	0.03					12.50	0.63
Wheat	CH NARA	linear	T[air]^{plot}	Linear	2021.00	560.38	569.65		-48.22	0.01					12.57	0.69
Wheat	CH NARA	linear	T[air]^{ref}	Linear	2015.00	767.46	777.80	15.62	-48.49	0.01					12.17	0.63
Wheat	CH NARA	linear	T[air]^{ref}	Linear	2016.00	767.46	777.80	15.62	-48.49	0.01					12.17	0.63
Wheat	CH NARA	linear	T[air]^{ref}	Linear	2017.00	527.51	536.83	9.59	-51.56	0.01					7.72	0.62
Wheat	CH NARA	linear	T[air]^{ref}	Linear	2018.00	626.13	635.56	17.12	-47.61	0.01					13.46	0.62
Wheat	CH NARA	linear	T[air]^{ref}	Linear	2019.00	526.34	535.04	18.19	-47.27	0.01					13.95	0.66
Wheat	CH NARA	linear	T[air]^{ref}	Linear	2021.00	592.17	601.44	16.39	-48.20	0.01					12.58	0.64
Wheat	CH NARA	thermal	T[air]^{plot}	Thermal time	2015.00	734.14	741.89									
Wheat	CH NARA	thermal	T[air]^{plot}	Thermal time	2016.00	734.14	741.89									
Wheat	CH NARA	thermal	T[air]^{plot}	Thermal time	2017.00	573.09	580.08	12.44								
Wheat	CH NARA	thermal	T[air]^{plot}	Thermal time	2018.00	586.56	593.63									
Wheat	CH NARA	thermal	T[air]^{plot}	Thermal time	2019.00	479.83	486.35									
Wheat	CH NARA	thermal	T[air]^{plot}	Thermal time	2021.00	553.97	560.92									
Wheat	CH NARA	thermal	T[air]^{ref}	Thermal time	2015.00	769.48	777.23	14.53								
Wheat	CH NARA	thermal	T[air]^{ref}	Thermal time	2016.00	769.48	777.23	14.53								
Wheat	CH NARA	thermal	T[air]^{ref}	Thermal time	2017.00	577.57	584.56	12.73								
Wheat	CH NARA	thermal	T[air]^{ref}	Thermal time	2018.00	621.68	628.75	15.37								
Wheat	CH NARA	thermal	T[air]^{ref}	Thermal time	2019.00	511.75	518.27	14.75								

Wheat	CH NARA	thermal	T[air]^{ref}	Thermal time	2021.00	589.95	596.91	14.95										
Wheat	CH NARA	wang	T[air]^{plot}	Wang-Engel	2015.00	728.60	738.94							14.79	0.54		11.65	0.60
Wheat	CH NARA	wang	T[air]^{plot}	Wang-Engel	2016.00	728.60	738.94							14.79	0.54		11.65	0.60
Wheat	CH NARA	wang	T[air]^{plot}	Wang-Engel	2017.00	538.75	548.07	9.81						11.70	0.51		8.33	0.57
Wheat	CH NARA	wang	T[air]^{plot}	Wang-Engel	2018.00	581.15	590.58							15.45	0.60		12.27	0.57
Wheat	CH NARA	wang	T[air]^{plot}	Wang-Engel	2019.00	484.56	493.26							16.66	0.58		12.84	0.65
Wheat	CH NARA	wang	T[air]^{plot}	Wang-Engel	2021.00	551.52	560.79							15.24	0.52		11.74	0.65
Wheat	CH NARA	wang	T[air]^{ref}	Wang-Engel	2015.00	753.40	763.74	14.21						14.90	0.55		11.28	0.61
Wheat	CH NARA	wang	T[air]^{ref}	Wang-Engel	2016.00	753.40	763.74	14.21						14.90	0.55		11.28	0.61
Wheat	CH NARA	wang	T[air]^{ref}	Wang-Engel	2017.00	537.99	547.31	9.79						11.53	0.50		8.28	0.58
Wheat	CH NARA	wang	T[air]^{ref}	Wang-Engel	2018.00	606.49	615.91	14.74						15.40	0.61		11.79	0.59
Wheat	CH NARA	wang	T[air]^{ref}	Wang-Engel	2019.00	508.73	517.43	15.32						16.68	0.61		12.10	0.61
Wheat	CH NARA	wang	T[air]^{ref}	Wang-Engel	2021.00	576.89	586.16	14.75						15.30	0.54		11.30	0.64
Wheat	FASTNET	asym	T[air]^{plot}	Asymptotic	2015.00	848.45	862.34		5.21			3.22	0.54				9.57	0.47
Wheat	FASTNET	asym	T[air]^{plot}	Asymptotic	2016.00	805.42	819.06		6.07			6.34	0.50				9.62	0.49
Wheat	FASTNET	asym	T[air]^{plot}	Asymptotic	2017.00	819.39	833.25	8.86	5.59			4.70	0.49				7.69	0.49
Wheat	FASTNET	asym	T[air]^{plot}	Asymptotic	2018.00	808.03	821.63		6.10			6.44	0.51				10.08	0.47
Wheat	FASTNET	asym	T[air]^{plot}	Asymptotic	2019.00	772.29	785.65		5.20			2.68	0.54				10.15	0.50
Wheat	FASTNET	asym	T[air]^{plot}	Asymptotic	2021.00	828.37	842.13		5.97			3.55	0.52				9.65	0.56
Wheat	FASTNET	asym	T[air]^{ref}	Asymptotic	2015.00	857.65	871.55		5.48			4.35	0.52				9.65	0.48
Wheat	FASTNET	asym	T[air]^{ref}	Asymptotic	2016.00	816.38	830.02		5.48			4.18	0.50				9.78	0.50
Wheat	FASTNET	asym	T[air]^{ref}	Asymptotic	2017.00	802.12	815.97		5.30			4.49	0.49				7.81	0.47
Wheat	FASTNET	asym	T[air]^{ref}	Asymptotic	2018.00	838.56	852.15	11.59	5.74			6.73	0.51				10.11	0.48
Wheat	FASTNET	asym	T[air]^{ref}	Asymptotic	2019.00	779.91	793.28		5.10			2.57	0.56				10.17	0.47
Wheat	FASTNET	asym	T[air]^{ref}	Asymptotic	2021.00	839.81	853.58		6.10			7.82	0.50				9.83	0.53
Wheat	FASTNET	bilinear	T[air]^{plot}	Bi-linear	2015.00	854.98	868.87		11.24	0.01	0.53						9.85	0.50
Wheat	FASTNET	bilinear	T[air]^{plot}	Bi-linear	2016.00	813.15	826.79		10.48	0.00	0.49						9.97	0.55
Wheat	FASTNET	bilinear	T[air]^{plot}	Bi-linear	2017.00	826.74	840.60	8.89	9.00	0.00	0.47						7.93	0.45
Wheat	FASTNET	bilinear	T[air]^{plot}	Bi-linear	2018.00	816.84	830.43		10.72	0.01	0.49						10.50	0.49
Wheat	FASTNET	bilinear	T[air]^{plot}	Bi-linear	2019.00	777.10	790.46		10.46	0.02	0.45						10.40	0.50
Wheat	FASTNET	bilinear	T[air]^{plot}	Bi-linear	2021.00	831.53	845.29		10.96	0.01	0.48						9.79	0.59
Wheat	FASTNET	bilinear	T[air]^{ref}	Bi-linear	2015.00	860.64	874.53		14.23	-0.02	0.66						9.77	0.50
Wheat	FASTNET	bilinear	T[air]^{ref}	Bi-linear	2016.00	820.58	834.22		12.60	-0.01	0.57						9.97	0.55
Wheat	FASTNET	bilinear	T[air]^{ref}	Bi-linear	2017.00	807.85	821.71		8.98	0.00	0.48						8.01	0.45
Wheat	FASTNET	bilinear	T[air]^{ref}	Bi-linear	2018.00	844.83	858.42	11.88	13.09	-0.01	0.59						10.40	0.49
Wheat	FASTNET	bilinear	T[air]^{ref}	Bi-linear	2019.00	785.27	798.64		12.80	0.00	0.55						10.43	0.48
Wheat	FASTNET	bilinear	T[air]^{ref}	Bi-linear	2021.00	840.71	854.48		12.60	-0.00	0.55						9.87	0.56
Wheat	FASTNET	gauss	^{plot}	Gaussian	2015.00	1067.38	1075.72	23.13								37.11	21.72	0.35
Wheat	FASTNET	gauss	^{plot}	Gaussian	2016.00	1014.09	1022.27	22.74								39.12	21.79	0.29
Wheat	FASTNET	gauss	^{plot}	Gaussian	2017.00	1054.65	1062.96	22.05								38.74	21.38	0.25
Wheat	FASTNET	gauss	^{plot}	Gaussian	2018.00	1026.09	1034.24	25.13								40.77	23.95	0.31
Wheat	FASTNET	gauss	^{plot}	Gaussian	2019.00	962.72	970.74	23.32								40.38	22.06	0.33
Wheat	FASTNET	gauss	^{plot}	Gaussian	2021.00	1054.23	1062.49	24.07								40.84	23.07	0.29
Wheat	FASTNET	gauss	^{ref}	Gaussian	2015.00	1067.38	1075.72	23.13								37.11	21.72	0.35
Wheat	FASTNET	gauss	^{ref}	Gaussian	2016.00	1014.09	1022.27	22.74								39.12	21.79	0.29
Wheat	FASTNET	gauss	^{ref}	Gaussian	2017.00	1054.65	1062.96	22.05								38.74	21.38	0.25
Wheat	FASTNET	gauss	^{ref}	Gaussian	2018.00	1026.09	1034.24	25.13								40.77	23.95	0.31
Wheat	FASTNET	gauss	^{ref}	Gaussian	2019.00	962.72	970.74	23.32								40.38	22.06	0.33
Wheat	FASTNET	gauss	^{ref}	Gaussian	2021.00	1054.23	1062.49	24.07								40.84	23.07	0.29
Wheat	FASTNET	linear	T[air]^{plot}	Linear	2015.00	891.23	902.34		-48.82	0.01							11.71	0.57
Wheat	FASTNET	linear	T[air]^{plot}	Linear	2016.00	840.05	850.96		-49.00	0.01							11.44	0.62
Wheat	FASTNET	linear	T[air]^{plot}	Linear	2017.00	839.46	850.54	9.85	-51.05	0.01							8.47	0.51

Wheat	FASTNET	linear	T[air]^{plot}	Linear	2018.00	848.11	858.98		-48.39	0.01						12.33	0.56
Wheat	FASTNET	linear	T[air]^{plot}	Linear	2019.00	808.76	819.46		-48.39	0.01						12.33	0.58
Wheat	FASTNET	linear	T[air]^{plot}	Linear	2021.00	866.67	877.68		-48.86	0.01						11.64	0.62
Wheat	FASTNET	linear	T[air]^{ref}	Linear	2015.00	900.68	911.80		-48.76	0.01						11.79	0.56
Wheat	FASTNET	linear	T[air]^{ref}	Linear	2016.00	850.25	861.16		-48.91	0.01						11.57	0.60
Wheat	FASTNET	linear	T[air]^{ref}	Linear	2017.00	819.63	830.71		-51.01	0.01						8.52	0.51
Wheat	FASTNET	linear	T[air]^{ref}	Linear	2018.00	855.91	866.79	12.35	-5.21	0.03						11.03	0.45
Wheat	FASTNET	linear	T[air]^{ref}	Linear	2019.00	791.25	801.94		-2.64	0.03						10.84	0.42
Wheat	FASTNET	linear	T[air]^{ref}	Linear	2021.00	876.51	887.53		-48.79	0.01						11.75	0.60
Wheat	FASTNET	thermal	T[air]^{plot}	Thermal time	2015.00	868.02	876.36										
Wheat	FASTNET	thermal	T[air]^{plot}	Thermal time	2016.00	830.79	838.97										
Wheat	FASTNET	thermal	T[air]^{plot}	Thermal time	2017.00	862.22	870.53	10.27									
Wheat	FASTNET	thermal	T[air]^{plot}	Thermal time	2018.00	826.88	835.04										
Wheat	FASTNET	thermal	T[air]^{plot}	Thermal time	2019.00	779.87	787.89										
Wheat	FASTNET	thermal	T[air]^{plot}	Thermal time	2021.00	841.85	850.11										
Wheat	FASTNET	thermal	T[air]^{ref}	Thermal time	2015.00	884.27	892.61										
Wheat	FASTNET	thermal	T[air]^{ref}	Thermal time	2016.00	846.22	854.41										
Wheat	FASTNET	thermal	T[air]^{ref}	Thermal time	2017.00	849.18	857.49										
Wheat	FASTNET	thermal	T[air]^{ref}	Thermal time	2018.00	862.90	871.05	12.43									
Wheat	FASTNET	thermal	T[air]^{ref}	Thermal time	2019.00	791.43	799.45										
Wheat	FASTNET	thermal	T[air]^{ref}	Thermal time	2021.00	858.59	866.85										
Wheat	FASTNET	wang	T[air]^{plot}	Wang-Engel	2015.00	859.45	870.56							16.50	0.69	10.13	0.46
Wheat	FASTNET	wang	T[air]^{plot}	Wang-Engel	2016.00	821.00	831.91							16.06	0.59	10.43	0.59
Wheat	FASTNET	wang	T[air]^{plot}	Wang-Engel	2017.00	848.98	860.06	9.99						15.24	0.57	8.80	0.47
Wheat	FASTNET	wang	T[air]^{plot}	Wang-Engel	2018.00	821.84	832.71							16.58	0.65	10.85	0.49
Wheat	FASTNET	wang	T[air]^{plot}	Wang-Engel	2019.00	786.51	797.20							17.37	0.67	10.99	0.53
Wheat	FASTNET	wang	T[air]^{plot}	Wang-Engel	2021.00	843.67	854.69							16.83	0.64	10.44	0.59
Wheat	FASTNET	wang	T[air]^{ref}	Wang-Engel	2015.00	860.26	871.38							16.29	0.67	9.84	0.48
Wheat	FASTNET	wang	T[air]^{ref}	Wang-Engel	2016.00	822.07	832.98							15.77	0.58	10.13	0.59
Wheat	FASTNET	wang	T[air]^{ref}	Wang-Engel	2017.00	824.74	835.83							14.89	0.56	8.70	0.47
Wheat	FASTNET	wang	T[air]^{ref}	Wang-Engel	2018.00	847.42	858.30	12.27						16.39	0.64	10.61	0.50
Wheat	FASTNET	wang	T[air]^{ref}	Wang-Engel	2019.00	787.96	798.65							17.10	0.66	10.67	0.52
Wheat	FASTNET	wang	T[air]^{ref}	Wang-Engel	2021.00	846.09	857.10							16.51	0.62	10.20	0.57
Wheat	MARKSMAN	asym	T[air]^{plot}	Asymptotic	2015.00	745.14	758.26		5.31			4.44	0.54			10.70	0.47
Wheat	MARKSMAN	asym	T[air]^{plot}	Asymptotic	2016.00	699.95	712.77		5.61			10.76	0.51			10.29	0.50
Wheat	MARKSMAN	asym	T[air]^{plot}	Asymptotic	2017.00	754.40	767.62	9.86	5.30			5.17	0.51			8.99	0.41
Wheat	MARKSMAN	asym	T[air]^{plot}	Asymptotic	2018.00	730.37	743.34		5.61			9.27	0.52			11.17	0.45
Wheat	MARKSMAN	asym	T[air]^{plot}	Asymptotic	2019.00	621.23	633.39		5.30			4.62	0.53			11.59	0.41
Wheat	MARKSMAN	asym	T[air]^{plot}	Asymptotic	2021.00	727.79	740.81		5.30			3.94	0.51			10.58	0.52
Wheat	MARKSMAN	asym	T[air]^{ref}	Asymptotic	2015.00	777.94	791.06	12.26	3.57			2.51	0.58			10.83	0.47
Wheat	MARKSMAN	asym	T[air]^{ref}	Asymptotic	2016.00	723.05	735.87	11.98	5.30			4.86	0.52			10.32	0.48
Wheat	MARKSMAN	asym	T[air]^{ref}	Asymptotic	2017.00	762.88	776.10	10.14	0.00			1.87	0.54			9.35	0.39
Wheat	MARKSMAN	asym	T[air]^{ref}	Asymptotic	2018.00	761.16	774.13	12.50	5.34			10.81	0.52			11.17	0.45
Wheat	MARKSMAN	asym	T[air]^{ref}	Asymptotic	2019.00	650.91	663.07	12.64	5.34			8.57	0.53			11.50	0.42
Wheat	MARKSMAN	asym	T[air]^{ref}	Asymptotic	2021.00	760.77	773.80	12.32	3.90			2.62	0.54			10.73	0.50
Wheat	MARKSMAN	bilinear	T[air]^{plot}	Bi-linear	2015.00	750.96	764.08		9.33	0.01	0.51					11.03	0.51
Wheat	MARKSMAN	bilinear	T[air]^{plot}	Bi-linear	2016.00	710.21	723.03		8.77	0.00	0.50					10.88	0.50
Wheat	MARKSMAN	bilinear	T[air]^{plot}	Bi-linear	2017.00	758.19	771.42	9.93	7.70	0.00	0.49					9.15	0.39
Wheat	MARKSMAN	bilinear	T[air]^{plot}	Bi-linear	2018.00	737.17	750.14		7.99	0.01	0.45					11.58	0.47
Wheat	MARKSMAN	bilinear	T[air]^{plot}	Bi-linear	2019.00	626.26	638.41		8.16	0.01	0.45					11.96	0.42
Wheat	MARKSMAN	bilinear	T[air]^{plot}	Bi-linear	2021.00	731.37	744.40		10.08	0.00	0.50					10.78	0.53
Wheat	MARKSMAN	bilinear	T[air]^{ref}	Bi-linear	2015.00	780.36	793.48	12.56	11.60	-0.00	0.60					10.97	0.49

Wheat	MARKSMAN	bilinear	T[air]^{ref}	Bi-linear	2016.00	731.44	744.26	12.61	8.73	0.00	0.51				10.78	0.50
Wheat	MARKSMAN	bilinear	T[air]^{ref}	Bi-linear	2017.00	758.70	771.92	9.95	7.70	0.00	0.50				9.17	0.39
Wheat	MARKSMAN	bilinear	T[air]^{ref}	Bi-linear	2018.00	767.72	780.69	13.01	7.98	0.01	0.46				11.56	0.47
Wheat	MARKSMAN	bilinear	T[air]^{ref}	Bi-linear	2019.00	655.55	667.71	12.94	8.56	0.01	0.47				11.82	0.41
Wheat	MARKSMAN	bilinear	T[air]^{ref}	Bi-linear	2021.00	761.89	774.91	12.53	10.84	0.00	0.53				10.79	0.52
Wheat	MARKSMAN	gauss	^{plot}	Gaussian	2015.00	938.93	946.80	26.12						39.13	24.52	0.35
Wheat	MARKSMAN	gauss	^{plot}	Gaussian	2016.00	872.48	880.17	24.28						39.45	23.14	0.31
Wheat	MARKSMAN	gauss	^{plot}	Gaussian	2017.00	946.12	954.06	24.37						39.98	23.21	0.31
Wheat	MARKSMAN	gauss	^{plot}	Gaussian	2018.00	914.29	922.07	26.58						41.83	24.91	0.36
Wheat	MARKSMAN	gauss	^{plot}	Gaussian	2019.00	766.24	773.54	24.81						43.02	23.59	0.32
Wheat	MARKSMAN	gauss	^{plot}	Gaussian	2021.00	916.45	924.26	25.56						41.91	24.03	0.35
Wheat	MARKSMAN	gauss	^{ref}	Gaussian	2015.00	938.93	946.80	26.12						39.13	24.52	0.35
Wheat	MARKSMAN	gauss	^{ref}	Gaussian	2016.00	872.48	880.17	24.28						39.45	23.14	0.31
Wheat	MARKSMAN	gauss	^{ref}	Gaussian	2017.00	946.12	954.06	24.37						39.98	23.21	0.31
Wheat	MARKSMAN	gauss	^{ref}	Gaussian	2018.00	914.29	922.07	26.58						41.83	24.91	0.36
Wheat	MARKSMAN	gauss	^{ref}	Gaussian	2019.00	766.24	773.54	24.81						43.02	23.59	0.32
Wheat	MARKSMAN	gauss	^{ref}	Gaussian	2021.00	916.45	924.26	25.56						41.91	24.03	0.35
Wheat	MARKSMAN	linear	T[air]^{plot}	Linear	2015.00	755.26	765.76		-8.26	0.02					11.39	0.56
Wheat	MARKSMAN	linear	T[air]^{plot}	Linear	2016.00	724.45	734.70		-48.68	0.01					11.91	0.56
Wheat	MARKSMAN	linear	T[air]^{plot}	Linear	2017.00	765.90	776.48	10.63	-50.27	0.01					9.60	0.44
Wheat	MARKSMAN	linear	T[air]^{plot}	Linear	2018.00	752.15	762.53		-48.12	0.01					12.72	0.52
Wheat	MARKSMAN	linear	T[air]^{plot}	Linear	2019.00	641.02	650.74		-47.68	0.01					13.35	0.48
Wheat	MARKSMAN	linear	T[air]^{plot}	Linear	2021.00	748.68	759.10		-48.63	0.01					11.98	0.56
Wheat	MARKSMAN	linear	T[air]^{ref}	Linear	2015.00	786.69	797.19	13.42	-9.38	0.02					11.43	0.53
Wheat	MARKSMAN	linear	T[air]^{ref}	Linear	2016.00	746.99	757.24	14.23	-48.71	0.01					11.86	0.54
Wheat	MARKSMAN	linear	T[air]^{ref}	Linear	2017.00	763.77	774.34	10.44	-22.33	0.01					9.47	0.42
Wheat	MARKSMAN	linear	T[air]^{ref}	Linear	2018.00	783.31	793.70	14.69	-48.14	0.01					12.69	0.52
Wheat	MARKSMAN	linear	T[air]^{ref}	Linear	2019.00	672.02	681.75	15.03	-47.74	0.01					13.27	0.48
Wheat	MARKSMAN	linear	T[air]^{ref}	Linear	2021.00	780.08	790.50	14.23	-48.62	0.01					12.00	0.55
Wheat	MARKSMAN	thermal	T[air]^{plot}	Thermal time	2015.00	767.29	775.17									
Wheat	MARKSMAN	thermal	T[air]^{plot}	Thermal time	2016.00	735.56	743.25									
Wheat	MARKSMAN	thermal	T[air]^{plot}	Thermal time	2017.00	792.96	800.89	12.30								
Wheat	MARKSMAN	thermal	T[air]^{plot}	Thermal time	2018.00	752.08	759.86									
Wheat	MARKSMAN	thermal	T[air]^{plot}	Thermal time	2019.00	634.23	641.52									
Wheat	MARKSMAN	thermal	T[air]^{plot}	Thermal time	2021.00	750.32	758.13									
Wheat	MARKSMAN	thermal	T[air]^{ref}	Thermal time	2015.00	802.55	810.42	14.33								
Wheat	MARKSMAN	thermal	T[air]^{ref}	Thermal time	2016.00	763.50	771.20	14.68								
Wheat	MARKSMAN	thermal	T[air]^{ref}	Thermal time	2017.00	797.86	805.79	12.61								
Wheat	MARKSMAN	thermal	T[air]^{ref}	Thermal time	2018.00	787.19	794.97	14.65								
Wheat	MARKSMAN	thermal	T[air]^{ref}	Thermal time	2019.00	665.33	672.62	13.76								
Wheat	MARKSMAN	thermal	T[air]^{ref}	Thermal time	2021.00	785.44	793.26	14.05								
Wheat	MARKSMAN	wang	T[air]^{plot}	Wang-Engel	2015.00	758.28	768.78					16.02	0.70		11.57	0.41
Wheat	MARKSMAN	wang	T[air]^{plot}	Wang-Engel	2016.00	723.49	733.75					15.17	0.61		11.82	0.44
Wheat	MARKSMAN	wang	T[air]^{plot}	Wang-Engel	2017.00	777.43	788.01	10.73				14.05	0.59		10.14	0.34
Wheat	MARKSMAN	wang	T[air]^{plot}	Wang-Engel	2018.00	750.47	760.85					15.86	0.65		12.56	0.38
Wheat	MARKSMAN	wang	T[air]^{plot}	Wang-Engel	2019.00	635.51	645.23					17.03	0.73		12.84	0.34
Wheat	MARKSMAN	wang	T[air]^{plot}	Wang-Engel	2021.00	742.92	753.34					16.08	0.64		11.58	0.47
Wheat	MARKSMAN	wang	T[air]^{ref}	Wang-Engel	2015.00	785.58	796.08	12.65				16.02	0.70		11.36	0.44
Wheat	MARKSMAN	wang	T[air]^{ref}	Wang-Engel	2016.00	741.83	752.08	13.37				15.04	0.60		11.51	0.47
Wheat	MARKSMAN	wang	T[air]^{ref}	Wang-Engel	2017.00	777.76	788.33	10.76				13.71	0.58		10.15	0.34
Wheat	MARKSMAN	wang	T[air]^{ref}	Wang-Engel	2018.00	779.42	789.80	13.64				15.91	0.66		12.39	0.43
Wheat	MARKSMAN	wang	T[air]^{ref}	Wang-Engel	2019.00	662.49	672.21	13.37				17.04	0.73		12.48	0.37

Wheat	MARKSMAN	wang	T[air]^{ref}	Wang-Engel	2021.00	770.62	781.04	13.12						16.06	0.64		11.39	0.51
Wheat	OSTKA STRZELECKA	asym	T[air]^{plot}	Asymptotic	2015.00	792.07	805.53		3.48			1.66	0.99				10.39	0.54
Wheat	OSTKA STRZELECKA	asym	T[air]^{plot}	Asymptotic	2016.00	786.84	800.06		2.80			1.21	1.01				11.77	0.62
Wheat	OSTKA STRZELECKA	asym	T[air]^{plot}	Asymptotic	2017.00	868.98	882.66	12.66	5.70			3.86	0.70				10.82	0.53
Wheat	OSTKA STRZELECKA	asym	T[air]^{plot}	Asymptotic	2018.00	812.48	825.80		4.20			1.78	0.93				12.85	0.52
Wheat	OSTKA STRZELECKA	asym	T[air]^{plot}	Asymptotic	2019.00	792.10	805.32		2.80			0.98	1.18				12.57	0.53
Wheat	OSTKA STRZELECKA	asym	T[air]^{plot}	Asymptotic	2021.00	785.72	798.90		2.80			1.14	1.09				12.66	0.56
Wheat	OSTKA STRZELECKA	asym	T[air]^{ref}	Asymptotic	2015.00	821.27	834.73	11.80	3.44			1.78	0.96				10.35	0.47
Wheat	OSTKA STRZELECKA	asym	T[air]^{ref}	Asymptotic	2016.00	814.07	827.29	13.99	2.80			1.45	0.92				11.99	0.53
Wheat	OSTKA STRZELECKA	asym	T[air]^{ref}	Asymptotic	2017.00	871.19	884.87	12.65	5.70			4.18	0.69				10.93	0.51
Wheat	OSTKA STRZELECKA	asym	T[air]^{ref}	Asymptotic	2018.00	844.87	858.18	14.21	3.30			1.59	0.96				12.89	0.44
Wheat	OSTKA STRZELECKA	asym	T[air]^{ref}	Asymptotic	2019.00	824.62	837.84	14.00	2.92			1.20	1.08				12.62	0.45
Wheat	OSTKA STRZELECKA	asym	T[air]^{ref}	Asymptotic	2021.00	818.73	831.90	14.50	2.80			1.34	1.00				12.75	0.49
Wheat	OSTKA STRZELECKA	bilinear	T[air]^{plot}	Bi-linear	2015.00	797.15	810.60		13.06	0.02	0.83						10.65	0.54
Wheat	OSTKA STRZELECKA	bilinear	T[air]^{plot}	Bi-linear	2016.00	787.66	800.88		9.64	0.03	0.55						11.81	0.59
Wheat	OSTKA STRZELECKA	bilinear	T[air]^{plot}	Bi-linear	2017.00	862.53	876.21	11.73	8.70	0.02	0.57						10.52	0.46
Wheat	OSTKA STRZELECKA	bilinear	T[air]^{plot}	Bi-linear	2018.00	814.78	828.10		12.84	0.03	0.74						13.00	0.49
Wheat	OSTKA STRZELECKA	bilinear	T[air]^{plot}	Bi-linear	2019.00	793.28	806.50		8.95	0.04	0.52						12.64	0.51
Wheat	OSTKA STRZELECKA	bilinear	T[air]^{plot}	Bi-linear	2021.00	786.00	799.17		10.15	0.03	0.59						12.69	0.55
Wheat	OSTKA STRZELECKA	bilinear	T[air]^{ref}	Bi-linear	2015.00	826.93	840.39	12.10	14.10	0.00	0.91						10.62	0.47
Wheat	OSTKA STRZELECKA	bilinear	T[air]^{ref}	Bi-linear	2016.00	816.00	829.23	13.97	9.62	0.03	0.59						12.11	0.51
Wheat	OSTKA STRZELECKA	bilinear	T[air]^{ref}	Bi-linear	2017.00	863.76	877.44	11.76	8.36	0.02	0.56						10.57	0.45
Wheat	OSTKA STRZELECKA	bilinear	T[air]^{ref}	Bi-linear	2018.00	848.54	861.86	14.38	11.52	0.02	0.70						13.12	0.42
Wheat	OSTKA STRZELECKA	bilinear	T[air]^{ref}	Bi-linear	2019.00	827.12	840.35	14.16	13.40	0.02	0.75						12.78	0.45
Wheat	OSTKA STRZELECKA	bilinear	T[air]^{ref}	Bi-linear	2021.00	820.05	833.22	14.49	10.17	0.03	0.62						12.83	0.48
Wheat	OSTKA STRZELECKA	gauss	^{plot}	Gaussian	2015.00	1061.42	1069.49	32.66								55.68	32.05	0.20
Wheat	OSTKA STRZELECKA	gauss	^{plot}	Gaussian	2016.00	997.90	1005.83	29.92								56.35	29.85	0.11
Wheat	OSTKA STRZELECKA	gauss	^{plot}	Gaussian	2017.00	1094.91	1103.12	30.66								56.19	29.94	0.22
Wheat	OSTKA STRZELECKA	gauss	^{plot}	Gaussian	2018.00	1038.50	1046.49	33.90								60.15	33.04	0.23
Wheat	OSTKA STRZELECKA	gauss	^{plot}	Gaussian	2019.00	1005.67	1013.61	31.58								57.99	31.00	0.20
Wheat	OSTKA STRZELECKA	gauss	^{plot}	Gaussian	2021.00	1015.37	1023.27	34.36								59.92	34.09	0.14
Wheat	OSTKA STRZELECKA	gauss	^{ref}	Gaussian	2015.00	1061.42	1069.49	32.66								55.68	32.05	0.20
Wheat	OSTKA STRZELECKA	gauss	^{ref}	Gaussian	2016.00	997.90	1005.83	29.92								56.35	29.85	0.11
Wheat	OSTKA STRZELECKA	gauss	^{ref}	Gaussian	2017.00	1094.91	1103.12	30.66								56.19	29.94	0.22
Wheat	OSTKA STRZELECKA	gauss	^{ref}	Gaussian	2018.00	1038.50	1046.49	33.90								60.15	33.04	0.23
Wheat	OSTKA STRZELECKA	gauss	^{ref}	Gaussian	2019.00	1005.67	1013.61	31.58								57.99	31.00	0.20
Wheat	OSTKA STRZELECKA	gauss	^{ref}	Gaussian	2021.00	1015.37	1023.27	34.36								59.92	34.09	0.14
Wheat	OSTKA STRZELECKA	linear	T[air]^{plot}	Linear	2015.00	806.23	816.99		-3.32	0.04							11.23	0.56
Wheat	OSTKA STRZELECKA	linear	T[air]^{plot}	Linear	2016.00	790.75	801.32		-3.03	0.04							12.12	0.58
Wheat	OSTKA STRZELECKA	linear	T[air]^{plot}	Linear	2017.00													
Wheat	OSTKA STRZELECKA	linear	T[air]^{plot}	Linear	2018.00	861.73	872.39		-45.33	0.01							16.76	0.43
Wheat	OSTKA STRZELECKA	linear	T[air]^{plot}	Linear	2019.00	837.33	847.90		-45.77	0.01							16.13	0.47
Wheat	OSTKA STRZELECKA	linear	T[air]^{plot}	Linear	2021.00	832.11	842.65		-45.58	0.01							16.41	0.47
Wheat	OSTKA STRZELECKA	linear	T[air]^{ref}	Linear	2015.00	893.77	904.54	16.75	-46.69	0.01							14.79	0.49
Wheat	OSTKA STRZELECKA	linear	T[air]^{ref}	Linear	2016.00	821.36	831.93	14.26	-4.35	0.04							12.55	0.49
Wheat	OSTKA STRZELECKA	linear	T[air]^{ref}	Linear	2017.00	888.53	899.48	13.29	-48.63	0.01							11.98	0.45
Wheat	OSTKA STRZELECKA	linear	T[air]^{ref}	Linear	2018.00	894.88	905.54	18.21	-45.37	0.01							16.71	0.43
Wheat	OSTKA STRZELECKA	linear	T[air]^{ref}	Linear	2019.00	829.20	839.78	14.29	-2.59	0.04							13.03	0.42
Wheat	OSTKA STRZELECKA	linear	T[air]^{ref}	Linear	2021.00	823.64	834.18	14.76	-3.51	0.04							13.19	0.46
Wheat	OSTKA STRZELECKA	thermal	T[air]^{plot}	Thermal time	2015.00	812.53	820.60											
Wheat	OSTKA STRZELECKA	thermal	T[air]^{plot}	Thermal time	2016.00	793.60	801.54											
Wheat	OSTKA STRZELECKA	thermal	T[air]^{plot}	Thermal time	2017.00	878.00	886.21	13.15										

Wheat	OSTKA STRZELECKA	thermal	T[air]^{plot}	Thermal time	2018.00	817.47	825.46											
Wheat	OSTKA STRZELECKA	thermal	T[air]^{plot}	Thermal time	2019.00	794.10	802.03											
Wheat	OSTKA STRZELECKA	thermal	T[air]^{plot}	Thermal time	2021.00	789.71	797.61											
Wheat	OSTKA STRZELECKA	thermal	T[air]^{ref}	Thermal time	2015.00	853.13	861.20	13.74										
Wheat	OSTKA STRZELECKA	thermal	T[air]^{ref}	Thermal time	2016.00	828.28	836.21	14.96										
Wheat	OSTKA STRZELECKA	thermal	T[air]^{ref}	Thermal time	2017.00	882.16	890.37	13.39										
Wheat	OSTKA STRZELECKA	thermal	T[air]^{ref}	Thermal time	2018.00	856.67	864.66	15.31										
Wheat	OSTKA STRZELECKA	thermal	T[air]^{ref}	Thermal time	2019.00	830.48	838.41	14.44										
Wheat	OSTKA STRZELECKA	thermal	T[air]^{ref}	Thermal time	2021.00	828.40	836.30	15.27										
Wheat	OSTKA STRZELECKA	wang	T[air]^{plot}	Wang-Engel	2015.00	830.13	840.90							17.62	1.16		12.60	0.40
Wheat	OSTKA STRZELECKA	wang	T[air]^{plot}	Wang-Engel	2016.00	829.67	840.25							18.16	1.04		14.72	0.57
Wheat	OSTKA STRZELECKA	wang	T[air]^{plot}	Wang-Engel	2017.00	898.49	909.43	14.15						17.05	0.92		12.44	0.50
Wheat	OSTKA STRZELECKA	wang	T[air]^{plot}	Wang-Engel	2018.00	844.45	855.10							17.94	1.07		15.21	0.51
Wheat	OSTKA STRZELECKA	wang	T[air]^{plot}	Wang-Engel	2019.00	827.42	838.00							18.78	1.16		15.17	0.50
Wheat	OSTKA STRZELECKA	wang	T[air]^{plot}	Wang-Engel	2021.00	826.33	836.87							18.20	1.09		15.74	0.50
Wheat	OSTKA STRZELECKA	wang	T[air]^{ref}	Wang-Engel	2015.00	844.90	855.66	12.61						17.43	1.13		11.65	0.37
Wheat	OSTKA STRZELECKA	wang	T[air]^{ref}	Wang-Engel	2016.00	843.72	854.30	16.32						17.79	1.00		13.98	0.53
Wheat	OSTKA STRZELECKA	wang	T[air]^{ref}	Wang-Engel	2017.00	898.11	909.05	14.01						16.77	0.91		12.42	0.48
Wheat	OSTKA STRZELECKA	wang	T[air]^{ref}	Wang-Engel	2018.00	868.67	879.32	16.31						17.67	1.05		14.58	0.47
Wheat	OSTKA STRZELECKA	wang	T[air]^{ref}	Wang-Engel	2019.00	847.19	857.76	15.79						18.49	1.12		14.22	0.45
Wheat	OSTKA STRZELECKA	wang	T[air]^{ref}	Wang-Engel	2021.00	848.78	859.32	16.56						17.94	1.06		14.92	0.45
Wheat	ROMANUS	asym	T[air]^{plot}	Asymptotic	2015.00	778.10	791.41		5.13			2.56	0.82				10.84	0.44
Wheat	ROMANUS	asym	T[air]^{plot}	Asymptotic	2016.00	785.43	798.40		6.50			5.11	0.65				14.33	0.47
Wheat	ROMANUS	asym	T[air]^{plot}	Asymptotic	2017.00	881.59	895.14	14.22	6.80			8.99	0.67				12.80	0.43
Wheat	ROMANUS	asym	T[air]^{plot}	Asymptotic	2018.00	816.09	829.22		5.70			3.12	0.72				15.43	0.40
Wheat	ROMANUS	asym	T[air]^{plot}	Asymptotic	2019.00	784.78	797.70		6.70			4.43	0.69				15.59	0.42
Wheat	ROMANUS	asym	T[air]^{plot}	Asymptotic	2021.00	816.38	829.61		6.50			3.97	0.69				14.17	0.49
Wheat	ROMANUS	asym	T[air]^{ref}	Asymptotic	2015.00	818.48	831.80	13.23	4.90			2.45	0.85				11.37	0.51
Wheat	ROMANUS	asym	T[air]^{ref}	Asymptotic	2016.00	815.74	828.72	18.54	6.01			9.81	0.61				14.76	0.58
Wheat	ROMANUS	asym	T[air]^{ref}	Asymptotic	2017.00	882.47	896.02	14.01	5.80			4.58	0.65				12.77	0.41
Wheat	ROMANUS	asym	T[air]^{ref}	Asymptotic	2018.00	857.23	870.36	18.22	6.00			3.74	0.70				16.04	0.47
Wheat	ROMANUS	asym	T[air]^{ref}	Asymptotic	2019.00	823.75	836.67	17.90	5.20			2.15	0.81				16.05	0.45
Wheat	ROMANUS	asym	T[air]^{ref}	Asymptotic	2021.00	857.98	871.20	17.87	6.50			4.12	0.69				14.84	0.56
Wheat	ROMANUS	bilinear	T[air]^{plot}	Bi-linear	2015.00	787.46	800.77		13.12	0.01	0.80						11.36	0.45
Wheat	ROMANUS	bilinear	T[air]^{plot}	Bi-linear	2016.00	790.56	803.53		12.06	0.02	0.62						14.72	0.46
Wheat	ROMANUS	bilinear	T[air]^{plot}	Bi-linear	2017.00	886.98	900.53	13.73	8.90	0.02	0.54						13.03	0.32
Wheat	ROMANUS	bilinear	T[air]^{plot}	Bi-linear	2018.00	819.42	832.55		9.07	0.03	0.52						15.69	0.35
Wheat	ROMANUS	bilinear	T[air]^{plot}	Bi-linear	2019.00	785.56	798.48		8.99	0.03	0.49						15.65	0.35
Wheat	ROMANUS	bilinear	T[air]^{plot}	Bi-linear	2021.00	820.59	833.81		10.81	0.03	0.59						14.51	0.44
Wheat	ROMANUS	bilinear	T[air]^{ref}	Bi-linear	2015.00	826.32	839.64	13.58	13.95	0.00	0.87						11.80	0.49
Wheat	ROMANUS	bilinear	T[air]^{ref}	Bi-linear	2016.00	822.39	835.37	18.39	12.19	0.01	0.65						15.27	0.54
Wheat	ROMANUS	bilinear	T[air]^{ref}	Bi-linear	2017.00	887.98	901.53	13.81	8.97	0.02	0.56						13.09	0.32
Wheat	ROMANUS	bilinear	T[air]^{ref}	Bi-linear	2018.00	860.36	873.49	17.72	12.22	0.02	0.68						16.29	0.40
Wheat	ROMANUS	bilinear	T[air]^{ref}	Bi-linear	2019.00	826.28	839.20	17.64	12.11	0.03	0.65						16.26	0.40
Wheat	ROMANUS	bilinear	T[air]^{ref}	Bi-linear	2021.00	861.50	874.72	17.25	12.23	0.02	0.67						15.10	0.49
Wheat	ROMANUS	gauss	^{plot}	Gaussian	2015.00	1023.59	1031.58	31.88								53.58	30.78	0.27
Wheat	ROMANUS	gauss	^{plot}	Gaussian	2016.00	937.02	944.80	28.63								54.03	27.97	0.22
Wheat	ROMANUS	gauss	^{plot}	Gaussian	2017.00	1049.57	1057.70	28.61								53.98	27.79	0.24
Wheat	ROMANUS	gauss	^{plot}	Gaussian	2018.00	991.54	999.42	32.82								58.05	31.82	0.25
Wheat	ROMANUS	gauss	^{plot}	Gaussian	2019.00	938.71	946.46	30.90								56.69	29.63	0.29
Wheat	ROMANUS	gauss	^{plot}	Gaussian	2021.00	1005.23	1013.17	31.69								57.79	30.93	0.22
Wheat	ROMANUS	gauss	^{ref}	Gaussian	2015.00	1023.59	1031.58	31.88								53.58	30.78	0.27

Wheat	ROMANUS	gauss	^{ref}	Gaussian	2016.00	937.02	944.80	28.63							54.03	27.97	0.22
Wheat	ROMANUS	gauss	^{ref}	Gaussian	2017.00	1049.57	1057.70	28.61							53.98	27.79	0.24
Wheat	ROMANUS	gauss	^{ref}	Gaussian	2018.00	991.54	999.42	32.82							58.05	31.82	0.25
Wheat	ROMANUS	gauss	^{ref}	Gaussian	2019.00	938.71	946.46	30.90							56.69	29.63	0.29
Wheat	ROMANUS	gauss	^{ref}	Gaussian	2021.00	1005.23	1013.17	31.69							57.79	30.93	0.22
Wheat	ROMANUS	linear	T[air]^{plot}	Linear	2015.00	841.36	852.01		-46.46	0.01						15.13	0.48
Wheat	ROMANUS	linear	T[air]^{plot}	Linear	2016.00	813.46	823.84		-45.24	0.01						16.90	0.50
Wheat	ROMANUS	linear	T[air]^{plot}	Linear	2017.00	905.14	915.97	15.62	-47.00	0.01						14.35	0.41
Wheat	ROMANUS	linear	T[air]^{plot}	Linear	2018.00	849.18	859.68		-44.03	0.01						18.65	0.42
Wheat	ROMANUS	linear	T[air]^{plot}	Linear	2019.00	811.53	821.87		-44.25	0.01						18.34	0.42
Wheat	ROMANUS	linear	T[air]^{plot}	Linear	2021.00	852.24	862.82		-44.93	0.01						17.35	0.48
Wheat	ROMANUS	linear	T[air]^{ref}	Linear	2015.00	879.85	890.51	18.16	-46.18	0.01						15.54	0.54
Wheat	ROMANUS	linear	T[air]^{ref}	Linear	2016.00	843.54	853.92	21.14	-44.97	0.01						17.29	0.56
Wheat	ROMANUS	linear	T[air]^{ref}	Linear	2017.00	905.31	916.15	15.63	-46.99	0.01						14.36	0.41
Wheat	ROMANUS	linear	T[air]^{ref}	Linear	2018.00	888.67	899.17	21.54	-43.72	0.01						19.10	0.48
Wheat	ROMANUS	linear	T[air]^{ref}	Linear	2019.00	850.96	861.30	21.23	-43.93	0.01						18.81	0.48
Wheat	ROMANUS	linear	T[air]^{ref}	Linear	2021.00	891.78	902.36	20.86	-44.61	0.01						17.81	0.54
Wheat	ROMANUS	thermal	T[air]^{plot}	Thermal time	2015.00	797.57	805.56										
Wheat	ROMANUS	thermal	T[air]^{plot}	Thermal time	2016.00	792.33	800.12										
Wheat	ROMANUS	thermal	T[air]^{plot}	Thermal time	2017.00	893.69	901.82	14.51									
Wheat	ROMANUS	thermal	T[air]^{plot}	Thermal time	2018.00	818.37	826.25										
Wheat	ROMANUS	thermal	T[air]^{plot}	Thermal time	2019.00	783.49	791.24										
Wheat	ROMANUS	thermal	T[air]^{plot}	Thermal time	2021.00	821.53	829.46										
Wheat	ROMANUS	thermal	T[air]^{ref}	Thermal time	2015.00	842.25	850.24	14.41									
Wheat	ROMANUS	thermal	T[air]^{ref}	Thermal time	2016.00	828.40	836.18	18.08									
Wheat	ROMANUS	thermal	T[air]^{ref}	Thermal time	2017.00	897.08	905.20	14.76									
Wheat	ROMANUS	thermal	T[air]^{ref}	Thermal time	2018.00	860.87	868.75	17.87									
Wheat	ROMANUS	thermal	T[air]^{ref}	Thermal time	2019.00	824.57	832.33	17.50									
Wheat	ROMANUS	thermal	T[air]^{ref}	Thermal time	2021.00	864.59	872.52	17.25									
Wheat	ROMANUS	wang	T[air]^{plot}	Wang-Engel	2015.00	803.30	813.96						17.35	1.06		12.41	0.40
Wheat	ROMANUS	wang	T[air]^{plot}	Wang-Engel	2016.00	808.75	819.13						17.24	0.82		16.37	0.55
Wheat	ROMANUS	wang	T[air]^{plot}	Wang-Engel	2017.00	910.21	921.05	16.06					16.46	0.81		14.61	0.42
Wheat	ROMANUS	wang	T[air]^{plot}	Wang-Engel	2018.00	838.46	848.96						17.59	0.95		17.49	0.41
Wheat	ROMANUS	wang	T[air]^{plot}	Wang-Engel	2019.00	804.02	814.36						18.12	0.97		17.47	0.42
Wheat	ROMANUS	wang	T[air]^{plot}	Wang-Engel	2021.00	840.17	850.75						17.61	0.91		16.18	0.49
Wheat	ROMANUS	wang	T[air]^{ref}	Wang-Engel	2015.00	836.95	847.60	13.92					17.25	1.06		12.53	0.44
Wheat	ROMANUS	wang	T[air]^{ref}	Wang-Engel	2016.00	833.72	844.10	21.12					16.80	0.78		16.35	0.61
Wheat	ROMANUS	wang	T[air]^{ref}	Wang-Engel	2017.00	910.38	921.22	16.05					16.15	0.80		14.63	0.42
Wheat	ROMANUS	wang	T[air]^{ref}	Wang-Engel	2018.00	875.12	885.62	19.64					17.52	0.95		17.70	0.44
Wheat	ROMANUS	wang	T[air]^{ref}	Wang-Engel	2019.00	839.80	850.14	19.54					18.06	0.98		17.61	0.44
Wheat	ROMANUS	wang	T[air]^{ref}	Wang-Engel	2021.00	876.66	887.23	19.21					17.48	0.91		16.41	0.53
Wheat	RUNAL	asym	T[air]^{plot}	Asymptotic	2015.00	853.02	866.57		2.80		2.03	0.66				12.90	0.55
Wheat	RUNAL	asym	T[air]^{plot}	Asymptotic	2016.00	809.47	822.69		5.20		7.06	0.55				13.72	0.61
Wheat	RUNAL	asym	T[air]^{plot}	Asymptotic	2017.00	762.11	775.38	10.74	3.59		6.39	0.54				9.05	0.57
Wheat	RUNAL	asym	T[air]^{plot}	Asymptotic	2018.00	851.64	865.00		7.90		10.86	0.66				15.00	0.51
Wheat	RUNAL	asym	T[air]^{plot}	Asymptotic	2019.00	764.10	777.03		2.60		1.20	0.86				13.94	0.57
Wheat	RUNAL	asym	T[air]^{plot}	Asymptotic	2021.00	820.89	834.16		5.40		5.70	0.58				13.95	0.59
Wheat	RUNAL	asym	T[air]^{ref}	Asymptotic	2015.00	881.71	895.26	15.37	5.12		7.94	0.58				12.88	0.55
Wheat	RUNAL	asym	T[air]^{ref}	Asymptotic	2016.00	839.55	852.77	17.07	5.12		11.36	0.56				13.57	0.61
Wheat	RUNAL	asym	T[air]^{ref}	Asymptotic	2017.00	762.53	775.80	10.77	3.34		8.29	0.54				9.12	0.56
Wheat	RUNAL	asym	T[air]^{ref}	Asymptotic	2018.00	862.86	876.22	16.42	5.10		6.36	0.58				13.52	0.58
Wheat	RUNAL	asym	T[air]^{ref}	Asymptotic	2019.00	806.88	819.81	17.61	7.86		4.05	0.65				14.71	0.56

Wheat	RUNAL	asym	T[air]^{ref}	Asymptotic	2021.00	852.05	865.32	17.04	5.10			5.14	0.58				13.85	0.59
Wheat	RUNAL	bilinear	T[air]^{plot}	Bi-linear	2015.00	852.09	865.63		8.58	0.01	0.51						12.85	0.57
Wheat	RUNAL	bilinear	T[air]^{plot}	Bi-linear	2016.00	808.34	821.56		7.44	0.02	0.45						13.64	0.59
Wheat	RUNAL	bilinear	T[air]^{plot}	Bi-linear	2017.00	751.46	764.73	10.32	5.99	0.01	0.50						8.55	0.57
Wheat	RUNAL	bilinear	T[air]^{plot}	Bi-linear	2018.00	832.50	845.86		8.75	0.01	0.51						13.64	0.57
Wheat	RUNAL	bilinear	T[air]^{plot}	Bi-linear	2019.00	764.01	776.94		8.30	0.03	0.42						13.94	0.58
Wheat	RUNAL	bilinear	T[air]^{plot}	Bi-linear	2021.00	820.03	833.30		8.62	0.02	0.49						13.89	0.59
Wheat	RUNAL	bilinear	T[air]^{ref}	Bi-linear	2015.00	882.87	896.42	15.54	11.10	0.00	0.63						12.79	0.57
Wheat	RUNAL	bilinear	T[air]^{ref}	Bi-linear	2016.00	840.94	854.16	16.99	10.86	0.01	0.58						13.66	0.59
Wheat	RUNAL	bilinear	T[air]^{ref}	Bi-linear	2017.00	753.91	767.18	10.40	5.89	0.01	0.50						8.65	0.57
Wheat	RUNAL	bilinear	T[air]^{ref}	Bi-linear	2018.00	864.22	877.59	16.58	11.07	0.00	0.61						13.60	0.58
Wheat	RUNAL	bilinear	T[air]^{ref}	Bi-linear	2019.00	794.71	807.64	16.65	11.10	0.02	0.55						13.81	0.57
Wheat	RUNAL	bilinear	T[air]^{ref}	Bi-linear	2021.00	852.36	865.63	16.94	10.37	0.01	0.57						13.90	0.58
Wheat	RUNAL	gauss	^{plot}	Gaussian	2015.00	1041.17	1049.30	29.23								45.67	26.75	0.41
Wheat	RUNAL	gauss	^{plot}	Gaussian	2016.00	984.87	992.80	29.16								45.90	28.02	0.29
Wheat	RUNAL	gauss	^{plot}	Gaussian	2017.00	985.03	993.00	27.32								46.46	26.79	0.21
Wheat	RUNAL	gauss	^{plot}	Gaussian	2018.00	1025.84	1033.86	31.20								49.17	29.71	0.32
Wheat	RUNAL	gauss	^{plot}	Gaussian	2019.00	927.71	935.46	29.43								47.94	28.00	0.32
Wheat	RUNAL	gauss	^{plot}	Gaussian	2021.00	1011.43	1019.39	31.76								49.29	30.41	0.30
Wheat	RUNAL	gauss	^{ref}	Gaussian	2015.00	1041.17	1049.30	29.23								45.67	26.75	0.41
Wheat	RUNAL	gauss	^{ref}	Gaussian	2016.00	984.87	992.80	29.16								45.90	28.02	0.29
Wheat	RUNAL	gauss	^{ref}	Gaussian	2017.00	985.03	993.00	27.32								46.46	26.79	0.21
Wheat	RUNAL	gauss	^{ref}	Gaussian	2018.00	1025.84	1033.86	31.20								49.17	29.71	0.32
Wheat	RUNAL	gauss	^{ref}	Gaussian	2019.00	927.71	935.46	29.43								47.94	28.00	0.32
Wheat	RUNAL	gauss	^{ref}	Gaussian	2021.00	1011.43	1019.39	31.76								49.29	30.41	0.30
Wheat	RUNAL	linear	T[air]^{plot}	Linear	2015.00	871.27	882.11		-47.05	0.01							14.28	0.63
Wheat	RUNAL	linear	T[air]^{plot}	Linear	2016.00	822.72	833.30		-46.62	0.01							14.89	0.64
Wheat	RUNAL	linear	T[air]^{plot}	Linear	2017.00	742.10	752.71	10.32	-51.19	0.01							8.26	0.62
Wheat	RUNAL	linear	T[air]^{plot}	Linear	2018.00	849.65	860.35		-46.50	0.01							15.07	0.62
Wheat	RUNAL	linear	T[air]^{plot}	Linear	2019.00	786.14	796.48		-45.88	0.01							15.98	0.63
Wheat	RUNAL	linear	T[air]^{plot}	Linear	2021.00	837.86	848.48		-46.25	0.01							15.43	0.62
Wheat	RUNAL	linear	T[air]^{ref}	Linear	2015.00	903.56	914.40	18.00	-47.06	0.01							14.26	0.62
Wheat	RUNAL	linear	T[air]^{ref}	Linear	2016.00	855.39	865.96	19.00	-46.64	0.01							14.87	0.63
Wheat	RUNAL	linear	T[air]^{ref}	Linear	2017.00	742.52	753.13	10.33	-51.18	0.01							8.28	0.61
Wheat	RUNAL	linear	T[air]^{ref}	Linear	2018.00	882.00	892.69	18.84	-46.54	0.01							15.02	0.62
Wheat	RUNAL	linear	T[air]^{ref}	Linear	2019.00	818.62	828.96	20.09	-45.93	0.01							15.89	0.63
Wheat	RUNAL	linear	T[air]^{ref}	Linear	2021.00	870.27	880.89	19.32	-46.29	0.01							15.37	0.62
Wheat	RUNAL	thermal	T[air]^{plot}	Thermal time	2015.00	862.30	870.43											
Wheat	RUNAL	thermal	T[air]^{plot}	Thermal time	2016.00	815.54	823.47											
Wheat	RUNAL	thermal	T[air]^{plot}	Thermal time	2017.00	800.93	808.89	12.58										
Wheat	RUNAL	thermal	T[air]^{plot}	Thermal time	2018.00	841.76	849.78											
Wheat	RUNAL	thermal	T[air]^{plot}	Thermal time	2019.00	762.90	770.66											
Wheat	RUNAL	thermal	T[air]^{plot}	Thermal time	2021.00	824.94	832.90											
Wheat	RUNAL	thermal	T[air]^{ref}	Thermal time	2015.00	898.27	906.40	15.68										
Wheat	RUNAL	thermal	T[air]^{ref}	Thermal time	2016.00	851.78	859.72	16.89										
Wheat	RUNAL	thermal	T[air]^{ref}	Thermal time	2017.00	806.88	814.84	12.81										
Wheat	RUNAL	thermal	T[air]^{ref}	Thermal time	2018.00	877.84	885.85	16.63										
Wheat	RUNAL	thermal	T[air]^{ref}	Thermal time	2019.00	795.43	803.18	16.33										
Wheat	RUNAL	thermal	T[air]^{ref}	Thermal time	2021.00	861.14	869.10	17.00										
Wheat	RUNAL	wang	T[air]^{plot}	Wang-Engel	2015.00	860.41	871.25							15.92	0.74		13.49	0.54
Wheat	RUNAL	wang	T[air]^{plot}	Wang-Engel	2016.00	820.24	830.82							16.08	0.70		14.63	0.60
Wheat	RUNAL	wang	T[air]^{plot}	Wang-Engel	2017.00	791.88	802.50	12.71						13.49	0.63		10.48	0.60

Wheat	RUNAL	wang	T[air]^{plot}	Wang-Engel	2018.00	840.41	851.10							15.91	0.71		14.32	0.58
Wheat	RUNAL	wang	T[air]^{plot}	Wang-Engel	2019.00	776.28	786.62							17.53	0.79		15.05	0.56
Wheat	RUNAL	wang	T[air]^{plot}	Wang-Engel	2021.00	833.73	844.35							16.22	0.72		15.02	0.58
Wheat	RUNAL	wang	T[air]^{ref}	Wang-Engel	2015.00	885.53	896.37	16.01						15.92	0.74		13.06	0.58
Wheat	RUNAL	wang	T[air]^{ref}	Wang-Engel	2016.00	846.82	857.40	18.17						16.00	0.70		14.20	0.63
Wheat	RUNAL	wang	T[air]^{ref}	Wang-Engel	2017.00	791.69	802.31	12.70						13.40	0.63		10.47	0.60
Wheat	RUNAL	wang	T[air]^{ref}	Wang-Engel	2018.00	867.94	878.63	17.56						15.84	0.71		13.98	0.62
Wheat	RUNAL	wang	T[air]^{ref}	Wang-Engel	2019.00	799.92	810.26	17.55						17.47	0.79		14.34	0.59
Wheat	RUNAL	wang	T[air]^{ref}	Wang-Engel	2021.00	860.64	871.26	18.12						16.11	0.72		14.59	0.61
Wheat	RYWALKA	asym	T[air]^{plot}	Asymptotic	2015.00	847.25	860.97		4.38			1.73	0.95				10.88	0.58
Wheat	RYWALKA	asym	T[air]^{plot}	Asymptotic	2016.00	835.98	849.39		6.74			5.21	0.67				12.83	0.50
Wheat	RYWALKA	asym	T[air]^{plot}	Asymptotic	2017.00	942.73	956.71	13.85	6.21			10.91	0.64				11.79	0.54
Wheat	RYWALKA	asym	T[air]^{plot}	Asymptotic	2018.00	862.35	875.94		3.54			1.21	1.05				12.99	0.54
Wheat	RYWALKA	asym	T[air]^{plot}	Asymptotic	2019.00	795.82	809.04		2.80			0.57	1.46				12.80	0.51
Wheat	RYWALKA	asym	T[air]^{plot}	Asymptotic	2021.00	839.54	853.04		3.33			1.03	1.12				12.57	0.58
Wheat	RYWALKA	asym	T[air]^{ref}	Asymptotic	2015.00	875.56	889.28	12.89	4.30			1.89	0.90				10.78	0.55
Wheat	RYWALKA	asym	T[air]^{ref}	Asymptotic	2016.00	853.71	867.12	14.32	3.79			1.56	0.88				12.47	0.50
Wheat	RYWALKA	asym	T[air]^{ref}	Asymptotic	2017.00	942.71	956.69	13.83	6.10			5.42	0.65				11.79	0.53
Wheat	RYWALKA	asym	T[air]^{ref}	Asymptotic	2018.00	894.92	908.51	14.88	3.56			1.47	0.94				13.03	0.50
Wheat	RYWALKA	asym	T[air]^{ref}	Asymptotic	2019.00	829.23	842.45	14.35	2.80			0.84	1.24				12.91	0.45
Wheat	RYWALKA	asym	T[air]^{ref}	Asymptotic	2021.00	871.94	885.44	14.78	3.35			1.30	0.99				12.61	0.54
Wheat	RYWALKA	bilinear	T[air]^{plot}	Bi-linear	2015.00	855.27	868.99		14.30	0.02	0.80						11.28	0.56
Wheat	RYWALKA	bilinear	T[air]^{plot}	Bi-linear	2016.00	831.43	844.84		10.61	0.03	0.53						12.56	0.51
Wheat	RYWALKA	bilinear	T[air]^{plot}	Bi-linear	2017.00	932.41	946.39	12.81	9.64	0.03	0.52						11.29	0.49
Wheat	RYWALKA	bilinear	T[air]^{plot}	Bi-linear	2018.00	866.54	880.13		14.20	0.03	0.72						13.24	0.51
Wheat	RYWALKA	bilinear	T[air]^{plot}	Bi-linear	2019.00	797.69	810.91		10.93	0.04	0.54						12.92	0.50
Wheat	RYWALKA	bilinear	T[air]^{plot}	Bi-linear	2021.00	842.86	856.36		11.39	0.04	0.58						12.77	0.55
Wheat	RYWALKA	bilinear	T[air]^{ref}	Bi-linear	2015.00	884.46	898.18	13.16	15.10	-0.00	0.88						11.20	0.53
Wheat	RYWALKA	bilinear	T[air]^{ref}	Bi-linear	2016.00	859.05	872.46	14.49	11.24	0.03	0.59						12.79	0.48
Wheat	RYWALKA	bilinear	T[air]^{ref}	Bi-linear	2017.00	933.73	947.71	12.92	11.64	0.03	0.61						11.36	0.49
Wheat	RYWALKA	bilinear	T[air]^{ref}	Bi-linear	2018.00	899.89	913.48	15.07	14.56	0.01	0.77						13.33	0.48
Wheat	RYWALKA	bilinear	T[air]^{ref}	Bi-linear	2019.00	831.45	844.67	14.47	13.94	0.03	0.71						13.05	0.45
Wheat	RYWALKA	bilinear	T[air]^{ref}	Bi-linear	2021.00	876.86	890.36	14.87	11.28	0.03	0.60						12.91	0.51
Wheat	RYWALKA	gauss	^{plot}	Gaussian	2015.00	1100.30	1108.53	30.68								51.00	29.39	0.30
Wheat	RYWALKA	gauss	^{plot}	Gaussian	2016.00	1023.60	1031.64	28.60								51.06	28.11	0.20
Wheat	RYWALKA	gauss	^{plot}	Gaussian	2017.00	1149.27	1157.65	29.45								52.25	28.35	0.28
Wheat	RYWALKA	gauss	^{plot}	Gaussian	2018.00	1082.29	1090.44	32.11								54.82	30.85	0.29
Wheat	RYWALKA	gauss	^{plot}	Gaussian	2019.00	997.08	1005.02	30.77								55.28	29.73	0.27
Wheat	RYWALKA	gauss	^{plot}	Gaussian	2021.00	1066.57	1074.67	32.35								54.86	31.38	0.25
Wheat	RYWALKA	gauss	^{ref}	Gaussian	2015.00	1100.30	1108.53	30.68								51.00	29.39	0.30
Wheat	RYWALKA	gauss	^{ref}	Gaussian	2016.00	1023.60	1031.64	28.60								51.06	28.11	0.20
Wheat	RYWALKA	gauss	^{ref}	Gaussian	2017.00	1149.27	1157.65	29.45								52.25	28.35	0.28
Wheat	RYWALKA	gauss	^{ref}	Gaussian	2018.00	1082.29	1090.44	32.11								54.82	30.85	0.29
Wheat	RYWALKA	gauss	^{ref}	Gaussian	2019.00	997.08	1005.02	30.77								55.28	29.73	0.27
Wheat	RYWALKA	gauss	^{ref}	Gaussian	2021.00	1066.57	1074.67	32.35								54.86	31.38	0.25
Wheat	RYWALKA	linear	T[air]^{plot}	Linear	2015.00	919.63	930.61		-46.26	0.01							15.42	0.51
Wheat	RYWALKA	linear	T[air]^{plot}	Linear	2016.00	873.97	884.70		-46.07	0.01							15.69	0.49
Wheat	RYWALKA	linear	T[air]^{plot}	Linear	2017.00	965.62	976.80	14.75	-47.81	0.01							13.16	0.47
Wheat	RYWALKA	linear	T[air]^{plot}	Linear	2018.00	912.99	923.87		-45.22	0.01							16.75	0.49
Wheat	RYWALKA	linear	T[air]^{plot}	Linear	2019.00	843.22	853.80		-45.44	0.01							16.60	0.48
Wheat	RYWALKA	linear	T[air]^{plot}	Linear	2021.00	842.74	853.55		-0.65	0.04							12.88	0.55
Wheat	RYWALKA	linear	T[air]^{ref}	Linear	2015.00	951.78	962.76	17.68	-46.31	0.01							15.35	0.52

Wheat	RYWALKA	linear	T[air]^{ref}	Linear	2016.00	899.64	910.37	17.99	-46.04	0.01						15.74	0.50
Wheat	RYWALKA	linear	T[air]^{ref}	Linear	2017.00	965.73	976.91	14.74	-47.81	0.01						13.17	0.47
Wheat	RYWALKA	linear	T[air]^{ref}	Linear	2018.00	904.08	914.96	15.33	-1.51	0.04						13.70	0.46
Wheat	RYWALKA	linear	T[air]^{ref}	Linear	2019.00	875.98	886.56	18.59	-45.50	0.01						16.52	0.49
Wheat	RYWALKA	linear	T[air]^{ref}	Linear	2021.00	925.62	936.42	18.60	-45.54	0.01						16.46	0.49
Wheat	RYWALKA	thermal	T[air]^{plot}	Thermal time	2015.00	859.92	868.16										
Wheat	RYWALKA	thermal	T[air]^{plot}	Thermal time	2016.00	830.72	838.77										
Wheat	RYWALKA	thermal	T[air]^{plot}	Thermal time	2017.00	933.41	941.80	13.30									
Wheat	RYWALKA	thermal	T[air]^{plot}	Thermal time	2018.00	865.12	873.28										
Wheat	RYWALKA	thermal	T[air]^{plot}	Thermal time	2019.00	794.11	802.05										
Wheat	RYWALKA	thermal	T[air]^{plot}	Thermal time	2021.00	840.95	849.05										
Wheat	RYWALKA	thermal	T[air]^{ref}	Thermal time	2015.00	898.71	906.94	14.09									
Wheat	RYWALKA	thermal	T[air]^{ref}	Thermal time	2016.00	862.49	870.54	14.86									
Wheat	RYWALKA	thermal	T[air]^{ref}	Thermal time	2017.00	936.64	945.03	13.49									
Wheat	RYWALKA	thermal	T[air]^{ref}	Thermal time	2018.00	903.17	911.32	15.49									
Wheat	RYWALKA	thermal	T[air]^{ref}	Thermal time	2019.00	829.60	837.53	14.47									
Wheat	RYWALKA	thermal	T[air]^{ref}	Thermal time	2021.00	878.41	886.51	15.20									
Wheat	RYWALKA	wang	T[air]^{plot}	Wang-Engel	2015.00	878.12	889.10							17.96	1.09	12.63	0.47
Wheat	RYWALKA	wang	T[air]^{plot}	Wang-Engel	2016.00	858.78	869.51							18.60	1.02	14.46	0.52
Wheat	RYWALKA	wang	T[air]^{plot}	Wang-Engel	2017.00	966.44	977.63	15.09						17.95	0.95	13.13	0.51
Wheat	RYWALKA	wang	T[air]^{plot}	Wang-Engel	2018.00	885.10	895.98							18.45	1.04	14.58	0.54
Wheat	RYWALKA	wang	T[air]^{plot}	Wang-Engel	2019.00	822.88	833.46							19.25	1.17	14.83	0.51
Wheat	RYWALKA	wang	T[air]^{plot}	Wang-Engel	2021.00	871.25	882.05							18.65	1.07	14.76	0.54
Wheat	RYWALKA	wang	T[air]^{ref}	Wang-Engel	2015.00	895.88	906.86	13.44						17.72	1.07	11.88	0.47
Wheat	RYWALKA	wang	T[air]^{ref}	Wang-Engel	2016.00	875.48	886.21	16.00						18.15	0.97	13.94	0.50
Wheat	RYWALKA	wang	T[air]^{ref}	Wang-Engel	2017.00	963.40	974.58	14.89						17.72	0.93	12.96	0.51
Wheat	RYWALKA	wang	T[air]^{ref}	Wang-Engel	2018.00	911.66	922.53	16.42						18.08	1.00	14.18	0.52
Wheat	RYWALKA	wang	T[air]^{ref}	Wang-Engel	2019.00	848.13	858.71	16.03						18.84	1.11	14.29	0.47
Wheat	RYWALKA	wang	T[air]^{ref}	Wang-Engel	2021.00	895.86	906.66	16.41						18.27	1.02	14.21	0.52
Wheat	SEMAFOR	asym	T[air]^{plot}	Asymptotic	2015.00	757.75	770.97		5.20			5.21	0.53			10.59	0.61
Wheat	SEMAFOR	asym	T[air]^{plot}	Asymptotic	2016.00	730.12	743.05		5.60			5.81	0.48			11.62	0.72
Wheat	SEMAFOR	asym	T[air]^{plot}	Asymptotic	2017.00	729.25	742.47	8.74	4.50			4.13	0.49			7.94	0.42
Wheat	SEMAFOR	asym	T[air]^{plot}	Asymptotic	2018.00	761.96	775.08		5.40			5.08	0.49			11.68	0.71
Wheat	SEMAFOR	asym	T[air]^{plot}	Asymptotic	2019.00	690.68	703.35		5.30			3.58	0.46			11.57	0.85
Wheat	SEMAFOR	asym	T[air]^{plot}	Asymptotic	2021.00	732.37	745.35		5.35			4.93	0.47			11.29	0.80
Wheat	SEMAFOR	asym	T[air]^{ref}	Asymptotic	2015.00	788.82	802.04	11.94	5.12			5.42	0.54			10.61	0.46
Wheat	SEMAFOR	asym	T[air]^{ref}	Asymptotic	2016.00	776.92	789.84	13.62	7.80			11.74	0.60			12.61	0.39
Wheat	SEMAFOR	asym	T[air]^{ref}	Asymptotic	2017.00	728.76	741.99	8.63	3.14			3.32	0.49			7.93	0.40
Wheat	SEMAFOR	asym	T[air]^{ref}	Asymptotic	2018.00	793.98	807.11	13.28	5.30			8.50	0.51			11.72	0.47
Wheat	SEMAFOR	asym	T[air]^{ref}	Asymptotic	2019.00	722.50	735.17	13.27	4.40			2.27	0.58			11.63	0.49
Wheat	SEMAFOR	asym	T[air]^{ref}	Asymptotic	2021.00	766.14	779.12	13.25	5.30			8.46	0.51			11.47	0.51
Wheat	SEMAFOR	bilinear	T[air]^{plot}	Bi-linear	2015.00	758.99	772.21		10.46	-0.00	0.56					10.63	0.63
Wheat	SEMAFOR	bilinear	T[air]^{plot}	Bi-linear	2016.00	734.98	747.91		9.66	-0.00	0.48					11.93	0.73
Wheat	SEMAFOR	bilinear	T[air]^{plot}	Bi-linear	2017.00	719.85	733.07	8.59	7.26	-0.00	0.49					7.59	0.47
Wheat	SEMAFOR	bilinear	T[air]^{plot}	Bi-linear	2018.00	766.03	779.16		9.05	0.00	0.47					11.92	0.69
Wheat	SEMAFOR	bilinear	T[air]^{plot}	Bi-linear	2019.00	693.99	706.65		8.92	0.01	0.40					11.79	0.83
Wheat	SEMAFOR	bilinear	T[air]^{plot}	Bi-linear	2021.00	732.11	745.09		10.47	-0.00	0.49					11.27	0.82
Wheat	SEMAFOR	bilinear	T[air]^{ref}	Bi-linear	2015.00	789.63	802.85	12.16	12.60	-0.01	0.64					10.65	0.49
Wheat	SEMAFOR	bilinear	T[air]^{ref}	Bi-linear	2016.00	766.98	779.91	13.56	11.30	-0.00	0.55					11.98	0.48
Wheat	SEMAFOR	bilinear	T[air]^{ref}	Bi-linear	2017.00	721.09	734.31	8.63	7.06	-0.00	0.49					7.64	0.47
Wheat	SEMAFOR	bilinear	T[air]^{ref}	Bi-linear	2018.00	798.38	811.51	13.68	11.10	0.00	0.55					11.99	0.49
Wheat	SEMAFOR	bilinear	T[air]^{ref}	Bi-linear	2019.00	725.65	738.32	13.70	12.28	0.01	0.54					11.83	0.51

Wheat	SEMAFOR	bilinear	T[air]^{ref}	Bi-linear	2021.00	766.49	779.47	13.62	11.60	-0.00	0.55						11.49	0.54
Wheat	SEMAFOR	gauss	^{plot}	Gaussian	2015.00	950.63	958.56	25.31								40.66	23.73	0.36
Wheat	SEMAFOR	gauss	^{plot}	Gaussian	2016.00	900.44	908.20	25.75								42.70	24.33	0.31
Wheat	SEMAFOR	gauss	^{plot}	Gaussian	2017.00	937.58	945.51	22.88								42.62	22.27	0.24
Wheat	SEMAFOR	gauss	^{plot}	Gaussian	2018.00	945.85	953.72	26.73								44.45	25.38	0.32
Wheat	SEMAFOR	gauss	^{plot}	Gaussian	2019.00	846.79	854.39	24.85								43.83	23.35	0.35
Wheat	SEMAFOR	gauss	^{plot}	Gaussian	2021.00	916.81	924.59	26.31								45.18	25.23	0.29
Wheat	SEMAFOR	gauss	^{ref}	Gaussian	2015.00	950.63	958.56	25.31								40.66	23.73	0.36
Wheat	SEMAFOR	gauss	^{ref}	Gaussian	2016.00	900.44	908.20	25.75								42.70	24.33	0.31
Wheat	SEMAFOR	gauss	^{ref}	Gaussian	2017.00	937.58	945.51	22.88								42.62	22.27	0.24
Wheat	SEMAFOR	gauss	^{ref}	Gaussian	2018.00	945.85	953.72	26.73								44.45	25.38	0.32
Wheat	SEMAFOR	gauss	^{ref}	Gaussian	2019.00	846.79	854.39	24.85								43.83	23.35	0.35
Wheat	SEMAFOR	gauss	^{ref}	Gaussian	2021.00	916.81	924.59	26.31								45.18	25.23	0.29
Wheat	SEMAFOR	linear	T[air]^{plot}	Linear	2015.00	785.34	795.92		-48.38	0.01							12.33	0.66
Wheat	SEMAFOR	linear	T[air]^{plot}	Linear	2016.00	751.69	762.03		-47.76	0.01							13.24	0.71
Wheat	SEMAFOR	linear	T[air]^{plot}	Linear	2017.00	717.30	727.88	8.58	-51.66	0.01							7.57	0.48
Wheat	SEMAFOR	linear	T[air]^{plot}	Linear	2018.00	786.43	796.93		-47.62	0.01							13.44	0.67
Wheat	SEMAFOR	linear	T[air]^{plot}	Linear	2019.00	698.08	708.21		-3.93	0.03							12.21	0.80
Wheat	SEMAFOR	linear	T[air]^{plot}	Linear	2021.00	753.82	764.20		-48.03	0.01							12.85	0.74
Wheat	SEMAFOR	linear	T[air]^{ref}	Linear	2015.00	820.82	831.39	14.84	-48.22	0.01							12.58	0.55
Wheat	SEMAFOR	linear	T[air]^{ref}	Linear	2016.00	787.04	797.38	16.31	-47.60	0.01							13.47	0.56
Wheat	SEMAFOR	linear	T[air]^{ref}	Linear	2017.00	717.99	728.57	8.59	-51.65	0.01							7.60	0.47
Wheat	SEMAFOR	linear	T[air]^{ref}	Linear	2018.00	804.10	814.60	13.97	-6.14	0.03							12.46	0.46
Wheat	SEMAFOR	linear	T[air]^{ref}	Linear	2019.00	730.04	740.17	13.81	-3.00	0.03							12.25	0.47
Wheat	SEMAFOR	linear	T[air]^{ref}	Linear	2021.00	772.66	783.04	13.74	-6.31	0.03							11.97	0.50
Wheat	SEMAFOR	thermal	T[air]^{plot}	Thermal time	2015.00	775.91	783.84											
Wheat	SEMAFOR	thermal	T[air]^{plot}	Thermal time	2016.00	748.94	756.70											
Wheat	SEMAFOR	thermal	T[air]^{plot}	Thermal time	2017.00	775.42	783.36	10.75										
Wheat	SEMAFOR	thermal	T[air]^{plot}	Thermal time	2018.00	780.07	787.94											
Wheat	SEMAFOR	thermal	T[air]^{plot}	Thermal time	2019.00	699.14	706.73											
Wheat	SEMAFOR	thermal	T[air]^{plot}	Thermal time	2021.00	748.72	756.51											
Wheat	SEMAFOR	thermal	T[air]^{ref}	Thermal time	2015.00	807.39	815.32	12.94										
Wheat	SEMAFOR	thermal	T[air]^{ref}	Thermal time	2016.00	779.48	787.23	13.93										
Wheat	SEMAFOR	thermal	T[air]^{ref}	Thermal time	2017.00	781.04	788.97	10.98										
Wheat	SEMAFOR	thermal	T[air]^{ref}	Thermal time	2018.00	811.85	819.73	14.16										
Wheat	SEMAFOR	thermal	T[air]^{ref}	Thermal time	2019.00	730.34	737.94	13.68										
Wheat	SEMAFOR	thermal	T[air]^{ref}	Thermal time	2021.00	780.83	788.62	13.81										
Wheat	SEMAFOR	wang	T[air]^{plot}	Wang-Engel	2015.00	759.69	770.27							15.48	0.66		10.78	0.59
Wheat	SEMAFOR	wang	T[air]^{plot}	Wang-Engel	2016.00	739.27	749.61							15.06	0.53		12.34	0.79
Wheat	SEMAFOR	wang	T[air]^{plot}	Wang-Engel	2017.00	741.15	751.72	10.72						12.59	0.51		8.50	0.61
Wheat	SEMAFOR	wang	T[air]^{plot}	Wang-Engel	2018.00	772.25	782.75							15.02	0.55		12.44	0.73
Wheat	SEMAFOR	wang	T[air]^{plot}	Wang-Engel	2019.00	699.10	709.23							16.73	0.56		12.28	0.87
Wheat	SEMAFOR	wang	T[air]^{plot}	Wang-Engel	2021.00	737.82	748.20							15.32	0.54		11.74	0.82
Wheat	SEMAFOR	wang	T[air]^{ref}	Wang-Engel	2015.00	788.47	799.05	12.16						15.94	0.69		10.69	0.48
Wheat	SEMAFOR	wang	T[air]^{ref}	Wang-Engel	2016.00	770.54	780.87	14.55						15.82	0.61		12.33	0.54
Wheat	SEMAFOR	wang	T[air]^{ref}	Wang-Engel	2017.00	740.56	751.13	10.67						12.21	0.51		8.48	0.61
Wheat	SEMAFOR	wang	T[air]^{ref}	Wang-Engel	2018.00	803.47	813.97	14.63						15.55	0.61		12.42	0.54
Wheat	SEMAFOR	wang	T[air]^{ref}	Wang-Engel	2019.00	727.93	738.06	15.24						17.04	0.64		12.11	0.62
Wheat	SEMAFOR	wang	T[air]^{ref}	Wang-Engel	2021.00	769.84	780.22	14.54						15.85	0.61		11.80	0.59
Wheat	TAMARO	asym	T[air]^{plot}	Asymptotic	2015.00	922.39	936.37		4.60			2.17	0.75				12.35	0.47
Wheat	TAMARO	asym	T[air]^{plot}	Asymptotic	2016.00	872.97	886.74		6.02			12.22	0.61				11.80	0.54
Wheat	TAMARO	asym	T[air]^{plot}	Asymptotic	2017.00	855.72	869.57	10.03	5.50			5.00	0.61				8.98	0.46

Wheat	TAMARO	asym	T[air]^{plot}	Asymptotic	2018.00	905.49	919.34		4.30			1.97	0.77		12.72	0.47
Wheat	TAMARO	asym	T[air]^{plot}	Asymptotic	2019.00	866.72	880.40		4.30			1.76	0.81		12.32	0.49
Wheat	TAMARO	asym	T[air]^{plot}	Asymptotic	2021.00	879.22	892.85		7.60			4.57	0.69		13.53	0.52
Wheat	TAMARO	asym	T[air]^{ref}	Asymptotic	2015.00	953.26	967.24	13.94	4.30			2.11	0.76		12.33	0.47
Wheat	TAMARO	asym	T[air]^{ref}	Asymptotic	2016.00	902.89	916.66	14.66	5.80			9.56	0.61		11.43	0.60
Wheat	TAMARO	asym	T[air]^{ref}	Asymptotic	2017.00	854.19	868.04	9.83	5.22			4.99	0.61		8.92	0.43
Wheat	TAMARO	asym	T[air]^{ref}	Asymptotic	2018.00	936.99	950.84	14.39	4.30			2.07	0.76		12.71	0.48
Wheat	TAMARO	asym	T[air]^{ref}	Asymptotic	2019.00	896.62	910.30	13.98	4.30			1.83	0.80		12.23	0.50
Wheat	TAMARO	asym	T[air]^{ref}	Asymptotic	2021.00	900.16	913.79	14.95	4.30			2.07	0.75		12.87	0.52
Wheat	TAMARO	bilinear	T[air]^{plot}	Bi-linear	2015.00	922.50	936.48		17.20	-0.03	0.88				12.36	0.49
Wheat	TAMARO	bilinear	T[air]^{plot}	Bi-linear	2016.00	875.93	889.70		10.37	0.01	0.58				11.96	0.56
Wheat	TAMARO	bilinear	T[air]^{plot}	Bi-linear	2017.00	844.67	858.52	9.27	8.21	0.01	0.56				8.57	0.40
Wheat	TAMARO	bilinear	T[air]^{plot}	Bi-linear	2018.00	906.29	920.14		18.31	-0.03	0.90				12.77	0.50
Wheat	TAMARO	bilinear	T[air]^{plot}	Bi-linear	2019.00	866.99	880.67		21.20	-0.09	0.99				12.33	0.51
Wheat	TAMARO	bilinear	T[air]^{plot}	Bi-linear	2021.00	868.73	882.37		20.76	-0.08	0.98				12.89	0.53
Wheat	TAMARO	bilinear	T[air]^{ref}	Bi-linear	2015.00	949.97	963.95	14.00	18.27	-0.08	0.95				12.15	0.50
Wheat	TAMARO	bilinear	T[air]^{ref}	Bi-linear	2016.00	907.02	920.79	14.86	12.80	-0.00	0.69				11.96	0.58
Wheat	TAMARO	bilinear	T[air]^{ref}	Bi-linear	2017.00	845.05	858.90	9.27	7.99	0.01	0.56				8.58	0.39
Wheat	TAMARO	bilinear	T[air]^{ref}	Bi-linear	2018.00	935.82	949.68	14.59	18.50	-0.06	0.93				12.65	0.51
Wheat	TAMARO	bilinear	T[air]^{ref}	Bi-linear	2019.00	896.43	910.12	14.13	20.10	-0.09	0.96				12.22	0.51
Wheat	TAMARO	bilinear	T[air]^{ref}	Bi-linear	2021.00	898.62	912.26	15.01	18.60	-0.06	0.92				12.78	0.54
Wheat	TAMARO	gauss	^{plot}	Gaussian	2015.00	1142.27	1150.66	28.92						47.27	27.54	0.32
Wheat	TAMARO	gauss	^{plot}	Gaussian	2016.00	1098.21	1106.47	28.65						48.11	27.93	0.23
Wheat	TAMARO	gauss	^{plot}	Gaussian	2017.00	1108.03	1116.34	27.06						48.52	26.86	0.15
Wheat	TAMARO	gauss	^{plot}	Gaussian	2018.00	1134.03	1142.34	31.01						50.93	30.01	0.26
Wheat	TAMARO	gauss	^{plot}	Gaussian	2019.00	1082.36	1090.56	29.11						49.61	28.32	0.24
Wheat	TAMARO	gauss	^{plot}	Gaussian	2021.00	1091.42	1099.60	31.43						51.86	30.77	0.22
Wheat	TAMARO	gauss	^{ref}	Gaussian	2015.00	1142.27	1150.66	28.92						47.27	27.54	0.32
Wheat	TAMARO	gauss	^{ref}	Gaussian	2016.00	1098.21	1106.47	28.65						48.11	27.93	0.23
Wheat	TAMARO	gauss	^{ref}	Gaussian	2017.00	1108.03	1116.34	27.06						48.52	26.86	0.15
Wheat	TAMARO	gauss	^{ref}	Gaussian	2018.00	1134.03	1142.34	31.01						50.93	30.01	0.26
Wheat	TAMARO	gauss	^{ref}	Gaussian	2019.00	1082.36	1090.56	29.11						49.61	28.32	0.24
Wheat	TAMARO	gauss	^{ref}	Gaussian	2021.00	1091.42	1099.60	31.43						51.86	30.77	0.22
Wheat	TAMARO	linear	T[air]^{plot}	Linear	2015.00	967.74	978.93		-46.36	0.01					15.27	0.53
Wheat	TAMARO	linear	T[air]^{plot}	Linear	2016.00	885.19	896.20		-6.11	0.03					12.58	0.55
Wheat	TAMARO	linear	T[air]^{plot}	Linear	2017.00	850.24	861.32	9.77	-50.77	0.01					8.88	0.44
Wheat	TAMARO	linear	T[air]^{plot}	Linear	2018.00	950.95	962.04		-45.97	0.01					15.84	0.50
Wheat	TAMARO	linear	T[air]^{plot}	Linear	2019.00	911.84	922.79		-46.26	0.01					15.42	0.54
Wheat	TAMARO	linear	T[air]^{plot}	Linear	2021.00	909.59	920.50		-45.96	0.01					15.86	0.53
Wheat	TAMARO	linear	T[air]^{ref}	Linear	2015.00	968.08	979.26	14.88	-4.08	0.04					13.21	0.47
Wheat	TAMARO	linear	T[air]^{ref}	Linear	2016.00	940.22	951.24	17.88	-47.23	0.01					14.02	0.61
Wheat	TAMARO	linear	T[air]^{ref}	Linear	2017.00	850.26	861.34	9.77	-50.77	0.01					8.88	0.44
Wheat	TAMARO	linear	T[air]^{ref}	Linear	2018.00	984.19	995.28	18.29	-45.98	0.01					15.83	0.52
Wheat	TAMARO	linear	T[air]^{ref}	Linear	2019.00	944.76	955.71	18.26	-46.27	0.01					15.40	0.56
Wheat	TAMARO	linear	T[air]^{ref}	Linear	2021.00	942.89	953.80	18.65	-45.96	0.01					15.85	0.55
Wheat	TAMARO	thermal	T[air]^{plot}	Thermal time	2015.00	935.41	943.79									
Wheat	TAMARO	thermal	T[air]^{plot}	Thermal time	2016.00	894.39	902.65									
Wheat	TAMARO	thermal	T[air]^{plot}	Thermal time	2017.00	894.97	903.29	11.77								
Wheat	TAMARO	thermal	T[air]^{plot}	Thermal time	2018.00	914.79	923.10									
Wheat	TAMARO	thermal	T[air]^{plot}	Thermal time	2019.00	875.69	883.90									
Wheat	TAMARO	thermal	T[air]^{plot}	Thermal time	2021.00	879.17	887.35									
Wheat	TAMARO	thermal	T[air]^{ref}	Thermal time	2015.00	973.41	981.80	15.01								

Wheat	TAMARO	thermal	T[air]^{ref}	Thermal time	2016.00	933.07	941.33	15.25										
Wheat	TAMARO	thermal	T[air]^{ref}	Thermal time	2017.00	899.95	908.26	11.97										
Wheat	TAMARO	thermal	T[air]^{ref}	Thermal time	2018.00	952.42	960.74	15.16										
Wheat	TAMARO	thermal	T[air]^{ref}	Thermal time	2019.00	910.22	918.43	14.56										
Wheat	TAMARO	thermal	T[air]^{ref}	Thermal time	2021.00	917.08	925.26	15.68										
Wheat	TAMARO	wang	T[air]^{plot}	Wang-Engel	2015.00	927.40	938.58							17.47	0.93		12.73	0.48
Wheat	TAMARO	wang	T[air]^{plot}	Wang-Engel	2016.00	888.84	899.85							17.10	0.81		12.79	0.59
Wheat	TAMARO	wang	T[air]^{plot}	Wang-Engel	2017.00	869.90	880.98	10.80						15.92	0.76		9.63	0.47
Wheat	TAMARO	wang	T[air]^{plot}	Wang-Engel	2018.00	913.93	925.01							17.66	0.90		13.32	0.53
Wheat	TAMARO	wang	T[air]^{plot}	Wang-Engel	2019.00	877.94	888.88							18.21	0.94		13.09	0.51
Wheat	TAMARO	wang	T[air]^{plot}	Wang-Engel	2021.00	878.97	889.88							17.72	0.90		13.64	0.54
Wheat	TAMARO	wang	T[air]^{ref}	Wang-Engel	2015.00	949.65	960.84	14.12						17.30	0.91		12.24	0.50
Wheat	TAMARO	wang	T[air]^{ref}	Wang-Engel	2016.00	911.39	922.40	15.52						16.90	0.80		12.29	0.61
Wheat	TAMARO	wang	T[air]^{ref}	Wang-Engel	2017.00	868.01	879.09	10.68						15.71	0.75		9.55	0.47
Wheat	TAMARO	wang	T[air]^{ref}	Wang-Engel	2018.00	939.03	950.11	15.33						17.42	0.88		12.93	0.55
Wheat	TAMARO	wang	T[air]^{ref}	Wang-Engel	2019.00	899.79	910.73	14.54						18.01	0.92		12.52	0.52
Wheat	TAMARO	wang	T[air]^{ref}	Wang-Engel	2021.00	903.15	914.06	15.68						17.49	0.88		13.16	0.56
Wheat	TORONTO	asym	T[air]^{plot}	Asymptotic	2015.00	753.45	766.62		5.61			9.21	0.53				10.74	0.60
Wheat	TORONTO	asym	T[air]^{plot}	Asymptotic	2016.00	741.94	755.02		5.21			4.18	0.51				10.95	0.58
Wheat	TORONTO	asym	T[air]^{plot}	Asymptotic	2017.00	728.21	741.38	9.18	4.54			6.60	0.49				8.18	0.45
Wheat	TORONTO	asym	T[air]^{plot}	Asymptotic	2018.00	746.82	759.89		6.12			10.39	0.54				11.23	0.56
Wheat	TORONTO	asym	T[air]^{plot}	Asymptotic	2019.00	631.31	643.59		5.60			4.16	0.53				11.20	0.63
Wheat	TORONTO	asym	T[air]^{plot}	Asymptotic	2021.00	745.10	758.17		5.62			9.72	0.52				11.13	0.59
Wheat	TORONTO	asym	T[air]^{ref}	Asymptotic	2015.00	784.66	797.84	12.50	5.30			8.38	0.52				10.79	0.50
Wheat	TORONTO	asym	T[air]^{ref}	Asymptotic	2016.00	772.48	785.56	12.41	5.70			6.02	0.51				11.00	0.48
Wheat	TORONTO	asym	T[air]^{ref}	Asymptotic	2017.00	727.34	740.51	9.18	3.68			7.15	0.48				8.15	0.46
Wheat	TORONTO	asym	T[air]^{ref}	Asymptotic	2018.00	775.90	788.98	12.81	5.20			5.21	0.51				11.14	0.49
Wheat	TORONTO	asym	T[air]^{ref}	Asymptotic	2019.00	668.59	680.86	13.03	8.10			5.97	0.60				11.64	0.46
Wheat	TORONTO	asym	T[air]^{ref}	Asymptotic	2021.00	777.26	790.34	12.88	3.30			2.17	0.58				11.22	0.49
Wheat	TORONTO	bilinear	T[air]^{plot}	Bi-linear	2015.00	759.65	772.83		8.92	0.01	0.49						11.09	0.61
Wheat	TORONTO	bilinear	T[air]^{plot}	Bi-linear	2016.00	745.25	758.33		8.88	0.01	0.47						11.14	0.59
Wheat	TORONTO	bilinear	T[air]^{plot}	Bi-linear	2017.00	721.12	734.29	9.11	7.33	-0.00	0.49						7.90	0.49
Wheat	TORONTO	bilinear	T[air]^{plot}	Bi-linear	2018.00	750.47	763.55		8.73	0.01	0.47						11.45	0.58
Wheat	TORONTO	bilinear	T[air]^{plot}	Bi-linear	2019.00	635.45	647.72		10.44	0.01	0.50						11.49	0.61
Wheat	TORONTO	bilinear	T[air]^{plot}	Bi-linear	2021.00	747.47	760.54		10.31	0.01	0.51						11.27	0.61
Wheat	TORONTO	bilinear	T[air]^{ref}	Bi-linear	2015.00	790.69	803.86	12.93	10.78	0.00	0.54						11.11	0.51
Wheat	TORONTO	bilinear	T[air]^{ref}	Bi-linear	2016.00	774.84	787.91	12.71	8.80	0.01	0.46						11.08	0.50
Wheat	TORONTO	bilinear	T[air]^{ref}	Bi-linear	2017.00	722.17	735.35	9.14	7.34	-0.00	0.50						7.95	0.49
Wheat	TORONTO	bilinear	T[air]^{ref}	Bi-linear	2018.00	780.41	793.49	13.08	10.68	0.01	0.52						11.40	0.50
Wheat	TORONTO	bilinear	T[air]^{ref}	Bi-linear	2019.00	662.95	675.23	12.97	11.70	0.01	0.53						11.27	0.50
Wheat	TORONTO	bilinear	T[air]^{ref}	Bi-linear	2021.00	777.54	790.61	13.08	11.10	0.01	0.53						11.23	0.52
Wheat	TORONTO	gauss	^{plot}	Gaussian	2015.00	946.65	954.55	26.84								37.59	24.34	0.43
Wheat	TORONTO	gauss	^{plot}	Gaussian	2016.00	931.92	939.77	26.01								38.77	24.80	0.30
Wheat	TORONTO	gauss	^{plot}	Gaussian	2017.00	950.84	958.74	25.87								39.11	24.84	0.29
Wheat	TORONTO	gauss	^{plot}	Gaussian	2018.00	951.19	959.03	29.05								40.49	27.30	0.35
Wheat	TORONTO	gauss	^{plot}	Gaussian	2019.00	803.47	810.84	27.71								41.78	26.37	0.32
Wheat	TORONTO	gauss	^{plot}	Gaussian	2021.00	948.78	956.63	28.35								41.45	26.98	0.32
Wheat	TORONTO	gauss	^{ref}	Gaussian	2015.00	946.65	954.55	26.84								37.59	24.34	0.43
Wheat	TORONTO	gauss	^{ref}	Gaussian	2016.00	931.92	939.77	26.01								38.77	24.80	0.30
Wheat	TORONTO	gauss	^{ref}	Gaussian	2017.00	950.84	958.74	25.87								39.11	24.84	0.29
Wheat	TORONTO	gauss	^{ref}	Gaussian	2018.00	951.19	959.03	29.05								40.49	27.30	0.35
Wheat	TORONTO	gauss	^{ref}	Gaussian	2019.00	803.47	810.84	27.71								41.78	26.37	0.32

Wheat	TORONTO	gauss	^{ref}	Gaussian	2021.00	948.78	956.63	28.35								41.45	26.98	0.32
Wheat	TORONTO	linear	T[air]^{plot}	Linear	2015.00	784.94	795.48		-48.06	0.01							12.81	0.63
Wheat	TORONTO	linear	T[air]^{plot}	Linear	2016.00	763.70	774.16		-48.31	0.01							12.44	0.62
Wheat	TORONTO	linear	T[air]^{plot}	Linear	2017.00	724.05	734.58	9.51	-51.30	0.01							8.10	0.53
Wheat	TORONTO	linear	T[air]^{plot}	Linear	2018.00	772.83	783.29		-47.88	0.01							13.06	0.61
Wheat	TORONTO	linear	T[air]^{plot}	Linear	2019.00	663.44	673.25		-47.29	0.01							13.92	0.63
Wheat	TORONTO	linear	T[air]^{plot}	Linear	2021.00	773.48	783.94		-47.85	0.01							13.11	0.62
Wheat	TORONTO	linear	T[air]^{ref}	Linear	2015.00	815.59	826.13	15.22	-48.10	0.01							12.74	0.56
Wheat	TORONTO	linear	T[air]^{ref}	Linear	2016.00	793.69	804.16	14.72	-48.38	0.01							12.35	0.54
Wheat	TORONTO	linear	T[air]^{ref}	Linear	2017.00	724.28	734.82	9.53	-51.30	0.01							8.11	0.53
Wheat	TORONTO	linear	T[air]^{ref}	Linear	2018.00	805.08	815.54	15.40	-47.87	0.01							13.09	0.54
Wheat	TORONTO	linear	T[air]^{ref}	Linear	2019.00	667.01	676.83	13.14	-1.53	0.03							11.68	0.47
Wheat	TORONTO	linear	T[air]^{ref}	Linear	2021.00	804.03	814.49	15.48	-47.91	0.01							13.03	0.55
Wheat	TORONTO	thermal	T[air]^{plot}	Thermal time	2015.00	771.67	779.58											
Wheat	TORONTO	thermal	T[air]^{plot}	Thermal time	2016.00	760.23	768.08											
Wheat	TORONTO	thermal	T[air]^{plot}	Thermal time	2017.00	773.19	781.09	11.64										
Wheat	TORONTO	thermal	T[air]^{plot}	Thermal time	2018.00	760.74	768.59											
Wheat	TORONTO	thermal	T[air]^{plot}	Thermal time	2019.00	638.64	646.01											
Wheat	TORONTO	thermal	T[air]^{plot}	Thermal time	2021.00	758.23	766.08											
Wheat	TORONTO	thermal	T[air]^{ref}	Thermal time	2015.00	803.36	811.26	13.82										
Wheat	TORONTO	thermal	T[air]^{ref}	Thermal time	2016.00	790.28	798.13	13.72										
Wheat	TORONTO	thermal	T[air]^{ref}	Thermal time	2017.00	777.71	785.61	11.92										
Wheat	TORONTO	thermal	T[air]^{ref}	Thermal time	2018.00	791.92	799.76	13.82										
Wheat	TORONTO	thermal	T[air]^{ref}	Thermal time	2019.00	665.66	673.02	13.02										
Wheat	TORONTO	thermal	T[air]^{ref}	Thermal time	2021.00	789.12	796.96	13.87										
Wheat	TORONTO	wang	T[air]^{plot}	Wang-Engel	2015.00	765.68	776.21							15.42	0.64		11.55	0.52
Wheat	TORONTO	wang	T[air]^{plot}	Wang-Engel	2016.00	754.75	765.21							15.30	0.59		11.83	0.53
Wheat	TORONTO	wang	T[air]^{plot}	Wang-Engel	2017.00	743.18	753.72	9.89						13.13	0.55		8.89	0.44
Wheat	TORONTO	wang	T[air]^{plot}	Wang-Engel	2018.00	758.69	769.15							15.64	0.63		12.07	0.51
Wheat	TORONTO	wang	T[air]^{plot}	Wang-Engel	2019.00	640.56	650.38							17.04	0.70		12.01	0.56
Wheat	TORONTO	wang	T[air]^{plot}	Wang-Engel	2021.00	755.54	766.00							16.02	0.65		11.88	0.54
Wheat	TORONTO	wang	T[air]^{ref}	Wang-Engel	2015.00	795.44	805.97	13.07						15.52	0.64		11.48	0.48
Wheat	TORONTO	wang	T[air]^{ref}	Wang-Engel	2016.00	783.01	793.47	13.28						15.50	0.60		11.66	0.49
Wheat	TORONTO	wang	T[air]^{ref}	Wang-Engel	2017.00	743.42	753.95	9.92						12.93	0.55		8.90	0.44
Wheat	TORONTO	wang	T[air]^{ref}	Wang-Engel	2018.00	787.81	798.27	13.60						15.78	0.63		11.94	0.48
Wheat	TORONTO	wang	T[air]^{ref}	Wang-Engel	2019.00	665.71	675.52	13.22						17.28	0.71		11.59	0.49
Wheat	TORONTO	wang	T[air]^{ref}	Wang-Engel	2021.00	784.92	795.38	13.50						16.09	0.65		11.77	0.49
Wheat	WINNETOU	asym	T[air]^{plot}	Asymptotic	2015.00	727.46	740.07		5.00			2.99	0.64				14.95	0.55
Wheat	WINNETOU	asym	T[air]^{plot}	Asymptotic	2016.00	664.78	677.05		5.20			3.20	0.62				13.78	0.62
Wheat	WINNETOU	asym	T[air]^{plot}	Asymptotic	2017.00	675.35	687.74	11.84	5.33			9.61	0.61				11.08	0.36
Wheat	WINNETOU	asym	T[air]^{plot}	Asymptotic	2018.00	708.06	720.50		5.10			3.36	0.64				15.43	0.50
Wheat	WINNETOU	asym	T[air]^{plot}	Asymptotic	2019.00	680.65	692.87		7.20			10.01	0.63				16.00	0.56
Wheat	WINNETOU	asym	T[air]^{plot}	Asymptotic	2021.00	868.42	881.92		5.10			3.07	0.64				14.42	0.54
Wheat	WINNETOU	asym	T[air]^{ref}	Asymptotic	2015.00	754.92	767.53	16.31	4.30			2.52	0.67				14.50	0.47
Wheat	WINNETOU	asym	T[air]^{ref}	Asymptotic	2016.00	694.42	706.69	16.06	5.60			4.79	0.61				13.55	0.51
Wheat	WINNETOU	asym	T[air]^{ref}	Asymptotic	2017.00	674.46	686.85	11.80	5.34			8.99	0.61				11.03	0.37
Wheat	WINNETOU	asym	T[air]^{ref}	Asymptotic	2018.00	736.76	749.21	16.64	5.20			4.46	0.63				15.02	0.44
Wheat	WINNETOU	asym	T[air]^{ref}	Asymptotic	2019.00	708.43	720.64	17.46	6.70			3.22	0.67				15.46	0.48
Wheat	WINNETOU	asym	T[air]^{ref}	Asymptotic	2021.00	895.55	909.06	15.83	4.60			2.68	0.66				14.06	0.47
Wheat	WINNETOU	bilinear	T[air]^{plot}	Bi-linear	2015.00	729.96	742.57		11.68	0.00	0.64						15.16	0.57
Wheat	WINNETOU	bilinear	T[air]^{plot}	Bi-linear	2016.00	660.20	672.47		21.80	-0.53	0.99						13.39	0.66
Wheat	WINNETOU	bilinear	T[air]^{plot}	Bi-linear	2017.00	671.53	683.91	11.44	8.00	0.01	0.54						10.84	0.34

Wheat	WINNETOU	bilinear	T[air]^{plot}	Bi-linear	2018.00	700.88	713.32		21.91	-0.34	1.05				14.79	0.55
Wheat	WINNETOU	bilinear	T[air]^{plot}	Bi-linear	2019.00	674.30	686.51		22.80	-0.52	1.02				15.38	0.58
Wheat	WINNETOU	bilinear	T[air]^{plot}	Bi-linear	2021.00	870.87	884.38		13.40	-0.01	0.71				14.59	0.56
Wheat	WINNETOU	bilinear	T[air]^{ref}	Bi-linear	2015.00	754.88	767.49	16.65	14.50	-0.03	0.79				14.50	0.50
Wheat	WINNETOU	bilinear	T[air]^{ref}	Bi-linear	2016.00	688.54	700.82	16.13	19.00	-0.12	0.91				13.10	0.56
Wheat	WINNETOU	bilinear	T[air]^{ref}	Bi-linear	2017.00	671.48	683.87	11.42	8.00	0.01	0.54				10.83	0.33
Wheat	WINNETOU	bilinear	T[air]^{ref}	Bi-linear	2018.00	735.88	748.33	16.99	14.28	-0.03	0.79				14.96	0.49
Wheat	WINNETOU	bilinear	T[air]^{ref}	Bi-linear	2019.00	704.01	716.23	17.04	21.50	-0.23	0.98				15.04	0.48
Wheat	WINNETOU	bilinear	T[air]^{ref}	Bi-linear	2021.00	895.99	909.49	16.16	14.10	-0.02	0.76				14.09	0.50
Wheat	WINNETOU	gauss	^{plot}	Gaussian	2015.00	879.35	886.91	31.03						48.34	29.36	0.34
Wheat	WINNETOU	gauss	^{plot}	Gaussian	2016.00	826.94	834.31	30.72						49.55	30.27	0.20
Wheat	WINNETOU	gauss	^{plot}	Gaussian	2017.00	842.08	849.51	29.64						51.59	29.55	0.12
Wheat	WINNETOU	gauss	^{plot}	Gaussian	2018.00	871.11	878.57	33.60						53.77	33.00	0.21
Wheat	WINNETOU	gauss	^{plot}	Gaussian	2019.00	817.53	824.86	31.23						51.60	30.31	0.26
Wheat	WINNETOU	gauss	^{plot}	Gaussian	2021.00	1061.36	1069.46	31.33						51.00	30.64	0.23
Wheat	WINNETOU	gauss	^{ref}	Gaussian	2015.00	879.35	886.91	31.03						48.34	29.36	0.34
Wheat	WINNETOU	gauss	^{ref}	Gaussian	2016.00	826.94	834.31	30.72						49.55	30.27	0.20
Wheat	WINNETOU	gauss	^{ref}	Gaussian	2017.00	842.08	849.51	29.64						51.59	29.55	0.12
Wheat	WINNETOU	gauss	^{ref}	Gaussian	2018.00	871.11	878.57	33.60						53.77	33.00	0.21
Wheat	WINNETOU	gauss	^{ref}	Gaussian	2019.00	817.53	824.86	31.23						51.60	30.31	0.26
Wheat	WINNETOU	gauss	^{ref}	Gaussian	2021.00	1061.36	1069.46	31.33						51.00	30.64	0.23
Wheat	WINNETOU	linear	T[air]^{plot}	Linear	2015.00	750.35	760.44		-44.92	0.01					17.36	0.59
Wheat	WINNETOU	linear	T[air]^{plot}	Linear	2016.00	691.28	701.09		-45.50	0.01					16.52	0.65
Wheat	WINNETOU	linear	T[air]^{plot}	Linear	2017.00	678.87	688.78	12.08	-48.98	0.01					11.48	0.34
Wheat	WINNETOU	linear	T[air]^{plot}	Linear	2018.00	729.94	739.89		-44.54	0.01					17.91	0.57
Wheat	WINNETOU	linear	T[air]^{plot}	Linear	2019.00	700.00	709.78		-44.20	0.01					18.41	0.58
Wheat	WINNETOU	linear	T[air]^{plot}	Linear	2021.00	895.36	906.16		-45.41	0.01					16.65	0.56
Wheat	WINNETOU	linear	T[air]^{ref}	Linear	2015.00	781.51	791.60	19.76	-45.12	0.01					17.08	0.52
Wheat	WINNETOU	linear	T[air]^{ref}	Linear	2016.00	723.07	732.88	19.64	-45.63	0.01					16.33	0.56
Wheat	WINNETOU	linear	T[air]^{ref}	Linear	2017.00	678.86	688.77	12.07	-48.98	0.01					11.48	0.34
Wheat	WINNETOU	linear	T[air]^{ref}	Linear	2018.00	761.11	771.06	20.25	-44.77	0.01					17.59	0.51
Wheat	WINNETOU	linear	T[air]^{ref}	Linear	2019.00	731.36	741.13	20.85	-44.45	0.01					18.05	0.52
Wheat	WINNETOU	linear	T[air]^{ref}	Linear	2021.00	925.98	936.78	18.81	-45.58	0.01					16.41	0.50
Wheat	WINNETOU	thermal	T[air]^{plot}	Thermal time	2015.00	738.02	745.58									
Wheat	WINNETOU	thermal	T[air]^{plot}	Thermal time	2016.00	685.41	692.77									
Wheat	WINNETOU	thermal	T[air]^{plot}	Thermal time	2017.00	690.29	697.72	13.28								
Wheat	WINNETOU	thermal	T[air]^{plot}	Thermal time	2018.00	722.05	729.51									
Wheat	WINNETOU	thermal	T[air]^{plot}	Thermal time	2019.00	687.74	695.07									
Wheat	WINNETOU	thermal	T[air]^{plot}	Thermal time	2021.00	885.30	893.40									
Wheat	WINNETOU	thermal	T[air]^{ref}	Thermal time	2015.00	772.01	779.57	18.34								
Wheat	WINNETOU	thermal	T[air]^{ref}	Thermal time	2016.00	718.27	725.63	17.77								
Wheat	WINNETOU	thermal	T[air]^{ref}	Thermal time	2017.00	693.00	700.44	13.44								
Wheat	WINNETOU	thermal	T[air]^{ref}	Thermal time	2018.00	755.37	762.84	18.58								
Wheat	WINNETOU	thermal	T[air]^{ref}	Thermal time	2019.00	718.47	725.80	18.35								
Wheat	WINNETOU	thermal	T[air]^{ref}	Thermal time	2021.00	918.11	926.21	17.44								
Wheat	WINNETOU	wang	T[air]^{plot}	Wang-Engel	2015.00	726.19	736.28					17.00	0.85		15.01	0.55
Wheat	WINNETOU	wang	T[air]^{plot}	Wang-Engel	2016.00	662.18	672.00					17.14	0.80		13.73	0.68
Wheat	WINNETOU	wang	T[air]^{plot}	Wang-Engel	2017.00	689.23	699.14	13.02				15.46	0.73		12.14	0.38
Wheat	WINNETOU	wang	T[air]^{plot}	Wang-Engel	2018.00	705.76	715.72					16.90	0.83		15.40	0.54
Wheat	WINNETOU	wang	T[air]^{plot}	Wang-Engel	2019.00	679.11	688.88					17.94	0.87		16.05	0.57
Wheat	WINNETOU	wang	T[air]^{plot}	Wang-Engel	2021.00	869.44	880.24					17.02	0.82		14.63	0.57
Wheat	WINNETOU	wang	T[air]^{ref}	Wang-Engel	2015.00	751.33	761.41	16.42				16.59	0.82		14.37	0.49

Wheat	WINNETOU	wang	T[air]^{ref}	Wang-Engel	2016.00	687.77	697.59	16.38	16.66	0.78	13.19	0.57
Wheat	WINNETOU	wang	T[air]^{ref}	Wang-Engel	2017.00	688.09	698.00	12.89	15.23	0.73	12.06	0.37
Wheat	WINNETOU	wang	T[air]^{ref}	Wang-Engel	2018.00	733.20	743.15	16.89	16.47	0.80	14.90	0.48
Wheat	WINNETOU	wang	T[air]^{ref}	Wang-Engel	2019.00	704.76	714.53	17.46	17.45	0.83	15.31	0.49
Wheat	WINNETOU	wang	T[air]^{ref}	Wang-Engel	2021.00	894.38	905.18	16.28	16.63	0.79	14.11	0.51
